# BALDR: A Web-based platform for informed comparison and prioritization of biomarker candidates for type 2 diabetes mellitus

**DOI:** 10.1371/journal.pcbi.1011403

**Published:** 2023-08-17

**Authors:** Agnete T. Lundgaard, Frédéric Burdet, Troels Siggaard, David Westergaard, Danai Vagiaki, Lisa Cantwell, Timo Röder, Dorte Vistisen, Thomas Sparsø, Giuseppe N. Giordano, Mark Ibberson, Karina Banasik, Søren Brunak

**Affiliations:** 1 Novo Nordisk Foundation Center for Protein Research, University of Copenhagen, Blegdamsvej 3B, Copenhagen, Denmark; 2 Vital-IT, Swiss Institute of Bioinformatics (SIB), Lausanne, Switzerland; 3 Clinical Epidemiological Research, Steno Diabetes Center Copenhagen, Herlev, Denmark; 4 Department of Public Health, University of Copenhagen, Copenhagen, Denmark; 5 Bioinformatics and Data Mining, Global Research Technologies, Novo Nordisk A/S, Måløv, Denmark; 6 Genetic and Molecular Epidemiology Unit, Lund University Diabetes Centre, Department of Clinical Sciences, Clinical Research Centre, Lund University, Skåne University Hospital, Malmö, Sweden; Kansas State University, UNITED STATES

## Abstract

Novel biomarkers are key to addressing the ongoing pandemic of type 2 diabetes mellitus. While new technologies have improved the potential of identifying such biomarkers, at the same time there is an increasing need for informed prioritization to ensure efficient downstream verification. We have built BALDR, an automated pipeline for biomarker comparison and prioritization in the context of diabetes. BALDR includes protein, gene, and disease data from major public repositories, text-mining data, and human and mouse experimental data from the IMI2 RHAPSODY consortium. These data are provided as easy-to-read figures and tables enabling direct comparison of up to 20 biomarker candidates for diabetes through the public website https://baldr.cpr.ku.dk.

## Introduction

The advance of high-throughput omics methods has given rise to large-scale data capture for biomarker discovery. However, with ever-increasing data density from these analyses, the ability to prioritize candidates of interest for follow-up experiments, as part of an efficient downstream verification process, has become increasingly necessary. While many online tools exist for the analysis of single molecular entities in the context of human disease, few tools are available for the comparison and prioritization of a large set of molecules as potential biomarkers [1–3]. With the rapid rise in type 2 diabetes mellitus (T2DM) incidence worldwide [4,5], there is an increasing need for new diagnostic and progression biomarkers. However, there are currently no integrative tools available for diabetes biomarker discovery or prioritization. To address this gap, we developed Biomarker AnaLysis for Diabetes Research (BALDR), a tool that automatically produces a comprehensive report that enables informed comparison of up to 20 targets for their relevance as biomarkers for T2DM.

Public databases such as UniProt [6], PHAROS [7], Open Targets [8], DrugBank [9], and the Human Protein Atlas (HPA) [10] contain vast amounts of functional, interactional, and disease-relevant information for the human proteome. In BALDR, we utilize these data to inform on the relevance of proteins as novel, biologically relevant biomarkers for diabetes. Moreover, we include observational and experimental data from the Risk Assessment and ProgreSsiOn of Diabetes (RHAPSODY) consortium, financed by the EU Innovative Medicines Initiative-2. Within RHAPSODY, a large quantity of omics data has been generated based on a federation of clinical cohorts that include patients in varying stages of T2DM [11], as well as human tissue analysis [12,13] and mouse experiments [14] conducted within the consortium. These data include analysis of human blood and pancreatic islets and mouse adipose, skeletal muscle, liver, and pancreatic islet tissue, measuring a total of over 16,000 proteins and protein-coding gene transcripts.

**Fig 1:**
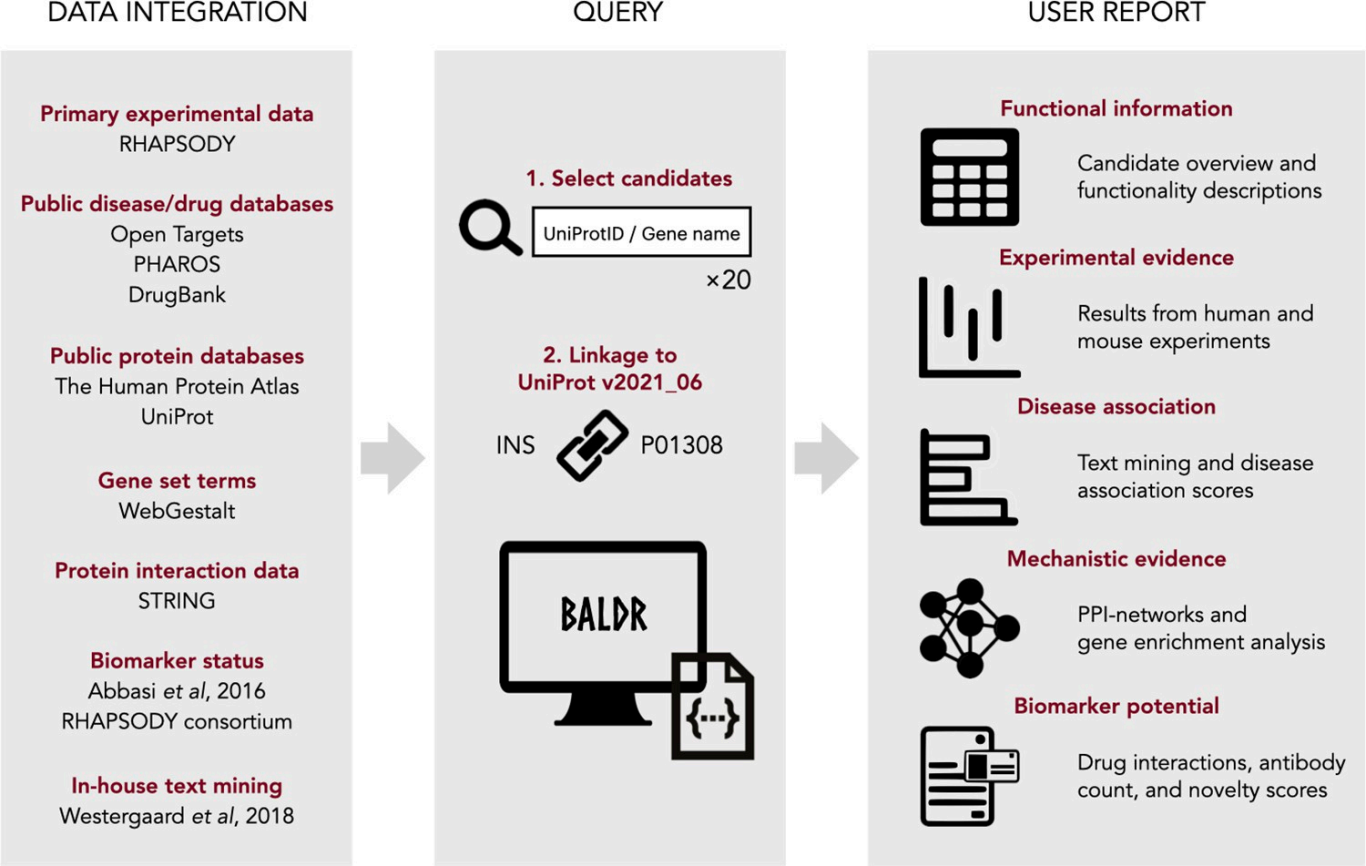
Schematic representation of the BALDR pipeline. The BALDR pipeline consists of three steps: Data integration, Query, and User report. In the Data integration step, data from multiple data sources is captured and consolidated into the BALDR database accessible to the BALDR source code. In the query step, the user selects up to 20 candidates using UniProt IDs or gene names. The targets are linked by UniProt IDs to the BALDR database and the BALDR source code runs using Rmarkdown. Lastly, a User report is produced containing five main sections: Functional information, Experimental evidence, Disease association, Mechanistic evidence, and Biomarker potential. Image credit: Openclipart.org.

BALDR is, to our knowledge, the first publicly available tool that allows for direct comparison between multiple protein biomarker candidates (Fig 1). We facilitate the integration and comparison of user candidates in a set of user-friendly graphics and tables that can readily be used for scientific publications. In the present version of BALDR, we use a combination of experimental data, text mining of full-length papers, and data obtained from major publicly available databases and repositories. This enables the user to make informed comparisons and prioritizations of biomarker candidates for T2DM. The framework is not limited to T2DM but can easily be adapted to other diseases of interest by changing the data capture workflow accordingly. BALDR is provided as open access through the public website (https://baldr.cpr.ku.dk).

## Design and implementation

### Data capture for biomarker prioritization

We extracted all relevant data from five public data sources included in the BALDR framework as a snapshot at the time of compilation. Specifically, we captured data from UniProt [6] (https://www.uniprot.org/), PHAROS [7] (https://pharos.nih.gov/), Open Targets [8] (https://www.opentargets.org/), DrugBank [9] (https://www.drugbank.com/), and the Human Protein Atlas (HPA) [10] (http://www.proteinatlas.org).

The UniProt database contains protein annotation and sequence data for the human proteome, as well as multiple other species. Data from the UniProt database was used for mapping genes and proteins using the UniProt and Ensembl IDs.

The PHAROS database is developed to improve drug discovery by gathering information for poorly annotated potential drug targets, especially G-coupled receptors, ion channels, and kinases. Within PHAROS, scores have been developed to characterize the knowledge level for targets. The PHAROS database was used for protein-specific information in the form of descriptions and aggregated scores for functionality and development level.

The Open Targets database consists of data collected from multiple sources on disease-target associations and summarizes this data as evidence scores for multiple data types, including text mining, genetic associations, and animal models. An overall score across data types is also provided. Diseases and phenotypes can be searched using the Experimental Factor Ontology (EFO) and Mondo Disease Ontology (MONDO). Open Target data was used here to inform on the evidence availability for targets in relation to diabetes mellitus (EFO_000400), as well as T2DM (MONDO_0005148), T1DM (MONDO_0005147), GDM (EFO_0004593), and other diabetes types.

The DrugBank database contains information on drugs and their association with molecular entities. Here, we include information on all drugs with evidence of interaction with a protein target. We include basic information on the drug, including ATC codes (if available), drug group, and pharmacological action. We also include links to evidence for these interactions through the DrugBank platform.

The HPA database contains information on the localization of proteins in cells, tissues, and organs based on omics and imaging data. HPA data was here used to inform on protein class and cellular location. Data from HPA was further used to identify proteins found in circulation for the RHAPSODY meta-analysis (see below).

The semi-automatic data capture pipeline is based on the Snakemake workflow system [15], integrating individual Python scripts for each database. The pipeline can be adapted to any disease with an Experimental Factor Ontology (EFO) label for the capture of disease-specific data from Open Targets and PHAROS. The data from these five major protein, gene, and disease databases resulted in 70 selected unique features for 20,343 of the 20,375 proteins available in UniProt (Table 1 and S1 File).

**Table 1:** Database overview.

Database	Variable count	Protein count
UniProt	5	20,375
PHAROS	12	20,268
Open Targets	42	18,881
DrugBank	11	3,025
The Human Protein Atlas (HPA)	4	19,139
**Total**	**70**	**20,343**

### Suggested biomarkers for type 2 diabetes

A table of known and suggested biomarkers for diabetes was compiled using three sources, i) Abbasi *et al* [16] conducted a systematic review of biomarkers T2DM incidence risk, identifying 167 potential biomarkers for diabetes. Of these, we included 48 proteins with a UniProt ID; ii) A biomarker task force within the RHAPSODY consortium curated a list of 33 biomarker candidates; iii) A network expansion was performed on the 81 biomarkers candidates found in i) and ii) using the Intomics InBio protein-protein interaction data [17]. These were compared to a network based on the 81 diabetes-associated sites previously identified by GWAS [18]. Of these, five genes were found to be overlapping and secreted. Finally, we performed a network expansion and identified 47 interaction partners, which interacted with at least two of the 81 suggested biomarkers with a confidence of at least 0.1. Together, 133 biomarkers were included as known or suggested biomarkers for T2DM. The full list is provided in S2 File.

### RHAPSODY data and meta-analysis

The RHAPSODY project aims to identify novel biomarkers for T2DM susceptibility and progression through analysis of omics data from twelve European cohorts comprising a total of 68,000 individuals with different stages of T2DM. Moreover, the project aimed to determine the link between alterations of insulin secretion and insulin action in the liver, adipose tissue, and skeletal muscle using pre-clinical mouse models. Lastly, β-cell functional study data from human and mouse pancreatic tissue are also available through the consortium. Dependent on species and tissue, multiple omics measurements have been performed, including, but not limited to, targeted metabolomics, lipidomics, genomics, transcriptomics, and proteomics.

Here, we included data from proteomics and transcriptomics experiments performed in human and mouse studies from four main analyses: Cox proportional hazard models for diabetes progression on 1,195 proteins using the SomaLogic SOMAscan platform in 1,188 individuals from the DCS and GoDARTS cohorts as described by Slieker *et al* [11]; differential RNA expression analysis in human islets and pancreatic tissues from 84 non-diabetic (ND) and 19 T2DM organ donors and 103 pancreatic tissue specimens (32 ND individuals, 36 T2DM individuals, 15 individuals with impaired glucose tolerance (IGT), and 20 with type 3c diabetes (T3D)), as described by Solimena *et al* [12] and Wigger *et al* [13]; and differential RNA expression analysis of adipose tissue, pancreatic islets, liver tissue, and skeletal muscle tissue from three mouse strains (C57Bl/6J, DBA/2J, and BALB/cJ) with high fat, high sucrose diet-induced glucose dysregulation (HF) compared to regular diet (RD)-fed animals, as described by Sanchez-Archidona *et al*[14].

We performed a sample size-based p-value meta-analysis, as described by Willer, Li, and Abecasis [19] across all experiments listed above. The resulting meta p-values were adjusted for multiple testing using false discovery rate (FDR) correction [20]. To rank the targets, we created a subset of targets that i) had an adjusted meta p-value under 0.05; ii) have been identified as found in circulation, either by expression or secretion to the bloodstream, using the Human Protein Atlas data; and iii) were included in the full-length text mining data, described below. To identify the most novel leads, the resulting targets were then inversely ranked by the number of co-mentions with diabetes so that the lowest number of co-mentions (zero) resulted in the best score of one. A total of 5035 (25%) proteins were assigned a rank between 1 and 710, with the best rank of 1 assigned to 367 targets with zero (0) co-mentions with diabetes.

### Text mining

Text mining was conducted on 15 million full-text scientific articles and their corresponding abstracts using the method described in Westergaard *et al* [21] with two settings. In short, 1,488,927 articles from the open-access PubMed Central corpus (PMC), 3,335,400 articles from Springer, and 11,697,096 articles from Elsevier spanning the period 1823–2016 were collected. Of these, 902,415 articles were removed as not written in English and additional 1,069,525 articles were removed in quality control, yielding 14,549,483 full-text articles for text mining. Additional 16,544,511 abstracts from MEDLINE were included for text mining. Text mining was performed using Named Entity Recognition (NER) using a dictionary comprised of gene names from the STRING database [36] and disease names from Disease Ontology [22]. A weighted score was calculated based on weighted counts, where co-occurrence within the same sentence or paragraph gives higher counts than co-occurrence within the same document. Weighted scores were calculated for both full-text articles and abstracts in two searches. For one search, all co-mentions between “diabetes” and all proteins were pulled, resulting in 16,366 proteins with at least one co-mention out of the 18,563 proteins (88%) identified in the text corpus. Similarly, we pulled co-mentions between disease terms for all diseases *but* diabetes and proteins and found at least one co-mention for 18,212 proteins (98%). Using full-text articles yielded on average 40 times more co-mentions for diabetes and 22 times more co-mentions for all other diseases compared to text mining of abstracts.

### Protein-protein interaction networks

Public protein-protein interaction (PPI) network data from the STRING database [23] were used to build PPI networks for each target. The networks were built to include physical interactions between candidates and interaction partners (primary interactions), as well as between interaction partners (secondary interactions). We used the recommended confidence threshold of 0.7 for the combined score to differentiate between high-confidence interactions and potential interactions [24]. The current version of BALDR implements the 9606 (human) physical links protein network data v11.5.

### Overrepresentation analysis

WebgestaltR [25] was used to conduct an overrepresentation analysis (ORA) of functional gene set terms on eight databases (see S3 File) in two separate analyses. In the first analysis, the enrichment analysis was conducted on the protein-protein interaction networks identified in the PPI analysis against all transcripts and proteins measured in RHAPSODY. Only terms including the candidates of interest were kept. In the second analysis, the enrichment analysis was conducted on the selected candidates in a shared analysis against all transcripts and proteins measured in RHAPSODY. In both analyses, enrichment is shown as significant (FDR adjusted p-value < 0.05) and not significant (FDR adjusted p-value ≥ 0.05). Based on the PPI networks, we performed gene enrichment analysis for 15,185 networks. Of these, 12,475 (82%) had at least one enriched gene term.

### User report

BALDR is built using R v4.2.1 [26] and R markdown v2.11 [27], a plain text editor that integrates R code and outputs with HTML code to produce formatted documents. The R packages ggplot2 v3.3.5, knitr v1.33 [28], and formattable v0.2.1 [29] were used to produce formatted figures and tables that are provided as stand-alone files in multiple formats. Figures are provided both as standard .png files and in vector format as .pdf files. Tables are likewise provided as .png files, as well as Excel sheets.

The BALDR report contains five sections that cover the different aspects of the input data:

- *Functional information*. To provide a basis for further exploration of the chosen candidates, we give a general overview of the candidates’ protein characteristics in the first table. The variables include subcellular location (HPA), protein family (PHAROS), protein class (HPA), and target development level (PHAROS). A second table presents functional descriptions from PHAROS, as well as their status as diabetes biomarkers.
- *Experimental evidence*. Experimental data from the RHAPSODY consortium is presented through volcano plots, scatter plots, and tables. We provide comparisons between candidates, as well as omics, species, and tissue types. Moreover, we provide a meta-analysis and ranking of all targets according to text-mining novelty for diabetes. This enables the user to compare their own results to those obtained in the RHAPSODY consortium and to assess the strength of the evidence across candidates.
- *Disease association*. To explore existing evidence for candidate-disease associations, we present disease-wide text-mining data (PHAROS and in-house text mining), diabetes-specific text-mining data from 15 million full-text articles (in-house text mining), and association scores for diabetes mellitus as a supergroup, as well as selected diabetes mellitus types such as T2DM, T1DM, and GDM (Open Targets).
- *Mechanistic evidence*. We use public protein-protein interaction data to produce protein-protein interaction networks for each candidate separately. Interaction partner information is made available to enable pathway exploration. These interaction networks also serve as a basis for gene set enrichment analysis for GO-terms, pathways, and disease/drug association. In a separate analysis, the input targets are analyzed together for enriched gene set terms to explore commonalities between candidates.
- *Biomarker potential*. In the last section, we explore the candidates in the context of their potential as biomarker candidates. We provide information on target-drug interaction from DrugBank, counts of commercially available antibodies, and a collective novelty score based on text mining from PHAROS. These data should be seen in the context of the candidates as general biomarkers and may not reflect their potential as diabetes biomarkers.

We showcase BALDR using the six novel biomarkers significantly associated with diabetes progression as measured by time to initiation of insulin treatment in the DCS and GoDARTS cohorts, as identified by Slieker *et al* [11]. The six proteins were identified as having the highest effect size for acceleration to insulin dependency out of 11 proteins significantly associated with the outcome. We compared these to three biomarkers associated with T2DM in the greatest number of incident cases included in the systematic review by Abbasi *et al* [16] (Table 2).

**Table 2:** Showcase targets from Slieker et al, 2021 [11] and Abbasi et al, 2016 [16].

Source	Gene name	Protein name	UniProt ID
[11]	CRELD1	Protein disulfide isomerase CRELD1	Q96HD1
	ENPP7	Ectonucleotide pyrophosphatase/phosphodiesterase family member 7	Q6UWV6
	FAS	Tumor necrosis factor receptor superfamily member 6	P25445
	GDF15	Growth/differentiation factor 15	Q99988
	IL18R1	Interleukin-18 receptor 1	Q13478
	RTN4R	Reticulon-4 receptor	Q9BZR6
[16]	CRP	C-reactive protein	P02741
	GGT1	Glutathione hydrolase 1 proenzyme	P19440
	GPT	Alanine aminotransferase 1	P24298

### Code availability and access

The code for BALDR and the depending data capture is freely available on GitHub at https://github.com/agnetelundgaard/BALDR.

## Results

### Automated data capture workflow combining multiple data sources

Comparing proteins as potential biomarkers, in the context of a specific disease such as T2DM, is challenging as candidate ranking can be based on different features, such as novelty, protein interaction partners, or gene-disease evidence depending on the research objective. To address this challenge, we made a semi-automated pipeline that captures public data from five major protein databases (UniProt, PHAROS, Open Targets, DrugBank, and the Human Protein Atlas (HPA)) that serve as the main data input for BALDR. We further enriched the report with data generated by text-mining of 15 million full-length papers [21], public protein-protein interaction network data [23], functional gene enrichment data [25], T2DM biomarker suggestions from internal and external sources [16], and published data from the RHAPSODY consortium [11–14]. We have gathered and standardized selected features for 20,343 human proteins for which comparative figures and tables can be produced through the BALDR pipeline. The easy-to-read graphics and tables can be used in publications with minimal editing required or imported by expert users to produce new graphics. Presenting these data directly to the user allows flexible decision-making and encourages transparency of the prioritization process.

### Example of a BALDR report

To showcase the value of BALDR, we compared nine potential diabetes biomarkers, six protein biomarkers identified by Slieker *et al* [11] as significantly correlated with T2DM progression in multiple cohorts, and three protein biomarkers identified by Abbasi *et al* [16] to have been positively associated with T2DM in the greatest number of incident cases (Table 2). We use this as a case scenario, where the task was to prioritize promising biomarker candidates identified in primary experimental data and we compare these to three highly studied T2DM biomarkers as a reference point. The example report on the nine biomarkers can be found in the supplement material (S4 File).

In section 1—Basic information, we find the cellular location, protein family, and PHAROS target development level of the candidates, as well as detailed functionality descriptions. ENPP7, GGT1, and GTP are enzymes involved in lipid or amino acid metabolism, while the other candidates belong to the non-IDG family, *i*.*e*., proteins that are not expected to be likely drug targets. The majority of these are receptors or receptor ligands involved in cellular signaling. One target, CRELD1, does not have a functionality description from PHAROS, so here, we need to extend our search to UniProt via the provided link. The section also provides summarized information from Open Targets on the association of candidates with diabetes. All except CRELD1 and ENPP7 have previously been directly or indirectly associated with T2DM, as well as other diabetes types.

In the next two sections, section 2 –Experiment evidence and section 3 –Disease association, we found that FAS, CRELD1, GDF15, CRP, and GPT were all found to be significantly associated with diabetes in a minimum of one RHAPSODY experiment, while only the Slieker *et al* [11] candidates were significant in the RHAPSODY cohort meta-analysis. In this meta-analysis, the highest ranked target is ENPP7, indicating that the target had the lowest count of co-mentions with diabetes, a proxy for the target’s novelty as a diabetes biomarker. Interestingly, when we look at the disease association data, we can see that ENPP7 has the lowest text mining score for all metrics but the highest fraction of diabetes co-mentions to all-disease co-mentions except for CRP, which also has the highest total number of co-mentions. Similarly, in the later section on target novelty (section 5.3), ENPP7 has one of the highest novelty scores. Taken together, ENPP7 appears to be understudied in general but may be relevant as a novel biomarker for diabetes. In contrast to ENPP7, we find that CRP, FAS, and GDF15 are the most studied targets for diabetes. GDF15 was identified by Slieker *et al* [11] to have the greatest effect size in their proteomics analysis, as shown in plot 2.1.2 in the report. Moreover, CRP was identified as the target with the highest association score for T2DM, as shown in section 3.2, followed by GDF15. Conversely, CRELD1 and ENPP7 were not associated with T2DM or any other diabetes type in Open Targets (sections 1.2 and 3.2).

In section 4 –Mechanistic evidence, protein-protein interaction (PPI) networks and gene enrichment are explored. The two largest networks are found for RTN4R and FAS, containing 201 and 117 interactions, respectively. This is explained by their role in large signaling pathways, as RTN4R is a receptor in the Rho signaling pathway responsible for the reorganization of the cytoskeleton, while FAS is a receptor for Caspase signaling mediating apoptosis. In contrast, we find no interaction partners for CRELD1 and no high-confidence interaction partners for ENPP7, GGT1, and GTP. IL18R1 has four high-confidence interaction partners, all related to interleukin signaling, including IL18 identified by Abbasi *et al* [16] as a biomarker for diabetes. CRP has multiple high-confidence interaction partners which have been identified as potential T2DM biomarkers through network expansion (FCN2 and CFH), by Abbasi *et al* [16](C3) or has been suggested by experts in the RHAPSODY consortium as potential novel targets (OLR1). FCN2, CFH, and C3 are involved in innate immunity, while OLR1 is involved in the degradation of oxidized low-density lipoprotein (oxLDL). Lastly, GDF15 has two high-confidence interaction partners, RET and GFRAL, that together mediate GDF15-induced food restriction.

Looking at the gene enrichment analysis we see a relatively limited number of shared terms for the KEGG pathways, GLAD4U diseases, and GLAD4U drugs databases (section 4.2.2). Most notably, FAS, IL18R1, and GDF15 have a non-significant enrichment in inflammatory pathways and anti-infectives. This is similarly seen in the gene enrichment analysis of their networks, where terms related to inflammation and apoptosis are found in most of the searched databases. Moreover, we find CRP, GGT1, GPT, and GDF15 to be highly enriched for multiple disease terms related to cardiovascular diseases and diabetes in the GLAD4U disease database. We also find multiple terms related to these diseases enriched for the individual candidate PPI networks (section 4.2.1). For example, CRP and GGT1 share enrichment for microvascular angina (182-fold compared to all genes in RHAPSODY) and likewise, we see enrichment in the two candidate PPI networks separately, although this enrichment is only significant for CRP.

In the last section, section 5 –Biomarker potential, our comparisons largely overlap with the results from sections 2 –Experimental evidence and 3 –Disease association, with FAS having the highest scores for antibody count and one of the lowest novelty scores. This indicates that FAS may be a suitable biomarker for T2DM, but has little novelty compared to other candidates. Meanwhile, ENPP7 and IL18R1 have some of the highest novelty scores, but importantly, a relatively high number of antibodies, making these targets suitable as biomarker candidates with high novelty. None of the Slieker *et al* [11] candidates have any known drug interactions registered in the DrugBank database, while two drugs for CRP are being investigated, both involved in LDL metabolism. Moreover, one and four drugs are approved or sold as nutraceuticals for GGT1 and GPT, respectively.

Comparing the candidates across the five sections, we find some general trends for the nine candidates included here. CRP, a well-known biomarker for T2DM, shows high scores for text mining, disease association scores, and low novelty, as well as interaction with several identified potential biomarkers and high enrichment for gene terms related to inflammation and metabolic disease. Surprisingly, CRP was not found to be significantly associated with T2DM in the RHAPSODY meta-analysis and only a single transcriptomics experiment in pancreas islets shows a significant association between CRP and T2DM. In contrast, CRELD1 and ENPP7 were significantly associated with T2DM in the RHAPSODY meta-analysis, but they are largely unstudied with no association with diabetes according to Open Targets, low text mining scores, and high novelty scores, as well as few or no interaction partners and enriched gene terms. Between these extremes, we find GDF15 to be greatly enriched in the RHAPSODY proteomics analysis, having high association scores for multiple diabetes types and medium-high text mining scores and novelty. GDF15 is, together with its interaction partners, involved in the regulation of food intake and is found to be associated with diabetes and cardiovascular diseases in gene enrichment analysis. Based on these findings, we find CRP to be a poor candidate as a novel biomarker, CRELD1 and ENPP7 to be interesting novel candidates that have largely unknown roles in diabetes, and GDF15 to be a candidate with a strong association with diabetes, but which have not been identified through systematic reviews such as Abbasi *et al* [16] or by biomarker prioritization frameworks such as network expansion.

### Comparison to other tools

While multiple online tools exist for the feature analysis of molecular entities in the context of human disease, we have not been able to identify a single biomarker tool that would fit the need for biomarker prioritization for T2DM, though several exist for cancer [30–33] or are aimed more broadly for diseases or drug targets [23,34–37]. Some of the tools require the upload of primary data [37,38] or are made as R packages, requiring a specialized skill set to use [35,36]. We were not able to access all tools that we identified in our search, as they either were behind a paywall [39] or were no longer available online [33,40,41].

These publicly available tools overlap with some of the included features in BALDR, such as protein-protein interaction networks [23,30,33,35] and gene set enrichment analysis[23,30,35]. In addition to these functions, BALDR also provides T2DM-specific experimental data from the RHAPSODY consortium, integration of multiple major databases, and text mining results from 15 million full-text articles. We show the utility of BALDR by comparing six biomarkers for T2DM identified by Slieker *et al* [11] with three highly studied T2DM-biomarkers from Abbasi *et al*[16]. Here, we discuss the molecular insights arising from multiple analyses and highlight candidates according to their novelty as diabetes biomarkers.

## Availability and future directions

The BALDR report is provided at https://baldr.cpr.ku.dk/, where the user can request a report on up to 20 biomarker candidates at a time. It includes all published proteomics and transcriptomics data from the RHAPSODY consortium. The report can be downloaded as a .zip-file directly through the website or e-mailed to the user. After compilation, all data processed are deleted immediately. All supplied information, including queried biomarker candidates, is treated as confidential. The website and finished report can be accessed through standard browsers and there are no computational restrictions for running the tool. The report, figures, and tables can be freely used and adapted by the user. Data from the RHAPSODY consortium can be freely used for academic and industrial purposes. All other data included in the BALDR report is publicly available from the primary sources listed above and should be used according to their individual licenses regarding use and redistribution.

The code for BALDR and its data capture scripts are freely available on GitHub at https://github.com/agnetelundgaard/BALDR. The provided source code for compiling the biomarker matrix that can be adapted to most diseases using EFO labels. However, BALDR has been specifically developed for T2DM, which means that some features, such as full-text text mining and experimental data, are not readily available for other diseases. Other sources for these data types will, therefore, be needed for a complete replication of the report, while protein-protein interaction networks, gene enrichment analysis, and functional information on targets are directly transferable and do not need adaptation to other diseases.

Our hope is that BALDR may serve as a framework for future applications enabling researchers to directly compare molecules of interest using public data without a prerequisite for specialized programming skills.

## Supporting information

S1 File Variables downloaded from the five databases. (TSV)

S2 File Known and suggested biomarkers for T2DM. (TSV)

S3 File Functional gene set terms from eight databases used in overrepresentation analysis. (TSV)

S4 File Example of a BALDR report using nine biomarker candidates from Slieker *et al* [11] and Abbasi *et al*. [16]. (HTML)
