# Supplementary material for: BALDR: A Web-based platform for informed comparison and prioritization of biomarker candidates for type 2 diabetes mellitus
Source: PLoS Comput Biol. 2023 Aug 17;19(8):e1011403. doi: 10.1371/journal.pcbi.1011403 (PMC10464978; doi:10.1371/journal.pcbi.1011403)
Supplement: S4 File — (HTML) [file pcbi.1011403.s004.html]

BALDR: Biomarker AnaLysis for Diabetes Research


Code 

- Show All Code
- Hide All Code

# BALDR: Biomarker AnaLysis for Diabetes Research

#### Version 0.8


# Identified targets

**Load list of proteins for prioritization**

Out of your selected genes:

P02741, P19440, P24298, P25445, Q13478, Q6UWV6, Q96HD1, Q99988,
Q9BZR6

the following targets was matched in our database to the gene
names:

CRP, GGT1, GPT, FAS, IL18R1, ENPP7, CRELD1, GDF15, RTN4R

# 1 Functional information

## 1.1 Target overview

### Table

Target overview

|  | Gene synonyms | Protein name | UniProt ID | Subcellular location | Protein family | Protein class | Target development level |
| --- | --- | --- | --- | --- | --- | --- | --- |
| CRELD1 | AVSD2, CIRRIN | Protein disulfide isomerase CRELD1, EC 5.3.4.1 | Q96HD1 | Nucleoli, Cytosol | Non-IDG | Disease related genes, Enzymes, Human disease related genes, Potential drug targets, Predicted intracellular proteins, Predicted membrane proteins | Tbio |
| CRP | PTX1 | C-reactive protein [Cleaved into: C-reactive protein | P02741 | Nucleoplasm, Vesicles, Intermediate filaments | Non-IDG | Cancer-related genes, Candidate cardiovascular disease genes, Human disease related genes, Plasma proteins, Predicted intracellular proteins, Predicted secreted proteins | Tbio |
| ENPP7 | alk-SMase, NPP7 | Ectonucleotide pyrophosphatase/ phosphodiesterase family member 7, E-NPP 7, NPP-7, EC 3.1.4.12 | Q6UWV6 | – | Enzyme | Enzymes, Metabolic proteins, Predicted intracellular proteins | Tbio |
| FAS | APO-1, APT1, CD95, FAS1, TNFRSF6 | Tumor necrosis factor receptor superfamily member 6 | P25445 | Nuclear bodies, Plasma membrane, Cytosol | Non-IDG | Cancer-related genes, Candidate cardiovascular disease genes, CD markers, Disease related genes, Human disease related genes, Metabolic proteins, Predicted membrane proteins, Predicted secreted proteins | Tbio |
| GDF15 | MIC-1, MIC1, NAG-1, PDF, PLAB, PTGFB | Growth/ differentiation factor 15, GDF-15 | Q99988 | Golgi apparatus | Non-IDG | Cancer-related genes, Disease related genes, Plasma proteins, Predicted intracellular proteins, Predicted secreted proteins | Tbio |
| GGT1 | CD224, D22S672, D22S732, GGT | Glutathione hydrolase 1 proenzyme, EC 3.4.19.13 | P19440 | Vesicles | Enzyme | Candidate cardiovascular disease genes, CD markers, Disease related genes, Enzymes, Metabolic proteins, Plasma proteins, Potential drug targets, Predicted intracellular proteins, Predicted membrane proteins | Tbio |
| GPT | ALT1, GPT1 | Alanine aminotransferase 1, ALT1, EC 2.6.1.2 | P24298 | – | Enzyme | Enzymes, Metabolic proteins, Plasma proteins, Predicted intracellular proteins | Tbio |
| IL18R1 | CD218a, IL-1Rrp, IL1RRP | Interleukin-18 receptor 1, IL-18R-1, IL-18R1, EC 3.2.2.6 | Q13478 | Mitochondria | Non-IDG | CD markers, Enzymes, Plasma proteins, Predicted membrane proteins | Tbio |
| RTN4R | NOGOR | Reticulon-4 receptor | Q9BZR6 | Plasma membrane, Actin filaments, Focal adhesion sites | Non-IDG | Disease related genes, Human disease related genes, Predicted membrane proteins | Tbio |

### About

Table content:

- **Protein name (UniProt)**: Name of gene
  product.
- **Gene synonyms (HPA)**: A list of different
  synonyms (in uppercase) for the (official) gene name provided by
  HPA.
- **UniProt ID (UniProt)**: Link to UniProt for
  additional information on the target.
- **Protein class (HPA)**: Classification of the
  protein target based on function and cellular compartment and/or disease
  and drug related information.
- **Subcellular location (HPA)**: HPA-assigned
  subcellular location.
- **Protein family (PHAROS)**: Protein family the
  target is assigned to.  Levels: GPCR, Nuclear Receptor, Kinase, Enzyme,
  Ion Channel, Transcription Factor, Transporter, Epigenetic, oGPCR,
  TF/Epigenetic, Non-IDG.  
  Special emphasis is given on four superfamilies that are central to the
  NIH IDG initiative: GPCRs, kinases, ion channels, and nuclear
  receptors.
- **Target development level (PHAROS)**: Target
  Development Level (TDL) (for more information on TDL see here).  TDL categorizes
  the target based on known information and potential drugability, with
  descending ranking:

  - Tclin: Approved drugs with known mechanism of action.
  - Tchem: If associated with small molecule activities in ChEMBL or
    DrugCentral.
  - Tbio: If not associated with small molecule or drug activities, but
    have a GO Molecular Function or Biological Process leaf term(s) with an
    Experimental Evidence code or/and have a confirmed OMIM phenotype.
  - Tdark: If not known drug or small molecule activities, and PubMed
    text-mining score from Jensen Lab <5 or/and ≥3 Gene RIFs or/and ≥50
    Antibodies available on http://antibodypedia.com.

en-dash (-) indicates that no information was available for the
target.

## 1.2 Functionality descriptions

### Table

Functionality description

|  | Functionality description | Diabetes associations | Suggested biomarker |
| --- | --- | --- | --- |
| CRELD1 | Description missing | – | No |
| CRP | Displays several functions associated with host defense: it promotes agglutination, bacterial capsular swelling, phagocytosis and complement fixation through its calcium-dependent binding to phosphorylcholine. Can interact with DNA and histones and may scavenge nuclear material released from damaged circulating cells. | T1DM, T2DM, GDM, Other | Abbasi et al, 2016 |
| ENPP7 | Converts sphingomyelin to ceramide and phosphocholine (PubMed:12885774, PubMed:12671034, PubMed:15205117, PubMed:28292932). Has also phospholipase C activity and can cleave phosphocholine from palmitoyl lyso-phosphatidylcholine (PubMed:12885774). Does not have nucleotide pyrophosphatase activity (PubMed:12885774). | – | No |
| FAS | Receptor for TNFSF6/FASLG. The adapter molecule FADD recruits caspase-8 to the activated receptor. The resulting death-inducing signaling complex (DISC) performs caspase-8 proteolytic activation which initiates the subsequent cascade of caspases (aspartate-specific cysteine proteases) mediating apoptosis. FAS-mediated apoptosis may have a role in the induction of peripheral tolerance, in the antigen-stimulated suicide of mature T-cells, or both. The secreted isoforms 2 to 6 block apoptosis (in vitro). | T1DM, T2DM, Other | No |
| GDF15 | Regulates food intake, energy expenditure and body weight in response to metabolic and toxin-induced stresses (PubMed:28953886, PubMed:28846097, PubMed:28846098, PubMed:28846099, PubMed:23468844, PubMed:29046435). Binds to its receptor, GFRAL, and activates GFRAL-expressing neurons localized in the area postrema and nucleus tractus solitarius of the brainstem (PubMed:28953886, PubMed:28846097, PubMed:28846098, PubMed:28846099). It then triggers the activation of neurons localized within the parabrachial nucleus and central amygdala, which contitutes part of the ‘emergency circuit’ that shapes feeding responses to stressful conditions (PubMed:28953886). On hepatocytes, inhibits growth hormone signaling (By similarity). | T1DM, T2DM, GDM, Other | No |
| GGT1 | Cleaves the gamma-glutamyl bond of extracellular glutathione (gamma-Glu-Cys-Gly), glutathione conjugates, and other gamma-glutamyl compounds. The metabolism of glutathione releases free glutamate and the dipeptide cysteinyl-glycine, which is hydrolyzed to cysteine and glycine by dipeptidases. In the presence of high concentrations of dipeptides and some amino acids, can also catalyze a transpeptidation reaction, transferring the gamma-glutamyl moiety to an acceptor amino acid to form a new gamma-glutamyl compound. Initiates extracellular glutathione (GSH) breakdown, provides cells with a local cysteine supply and contributes to maintain intracellular GSH level. It is part of the cell antioxidant defense mechanism. Isoform 3 seems to be inactive. | T2DM | Abbasi et al, 2016 |
| GPT | Catalyzes the reversible transamination between alanine and 2-oxoglutarate to form pyruvate and glutamate. Participates in cellular nitrogen metabolism and also in liver gluconeogenesis starting with precursors transported from skeletal muscles (By similarity). | T1DM, T2DM, GDM, Other | Abbasi et al, 2016 |
| IL18R1 | Within the IL18 receptor complex, responsible for the binding of the proinflammatory cytokine IL18, but not IL1A nor IL1B (PubMed:8626725, PubMed:14528293, PubMed:25261253, PubMed:25500532). Involved in IL18-mediated IFNG synthesis from T-helper 1 (Th1) cells (PubMed:10653850). Contributes to IL18-induced cytokine production, either independently of SLC12A3, or as a complex with SLC12A3 (By similarity). | T2DM | No |
| RTN4R | Receptor for RTN4, OMG and MAG (PubMed:12037567, PubMed:12068310, PubMed:12426574, PubMed:12089450, PubMed:16712417, PubMed:18411262, PubMed:12839991, PubMed:19052207). Functions as receptor for the sialylated gangliosides GT1b and GM1 (PubMed:18411262). Besides, functions as receptor for chondroitin sulfate proteoglycans (By similarity). Can also bind heparin (By similarity). Intracellular signaling cascades are triggered via the coreceptor NGFR (PubMed:12426574). Signaling mediates activation of Rho and downstream reorganization of the actin cytoskeleton (PubMed:16712417, PubMed:22325200). Mediates axonal growth inhibition (PubMed:12839991, PubMed:19052207, PubMed:28892071). Plays a role in regulating axon regeneration and neuronal plasticity in the adult central nervous system. Plays a role in postnatal brain development. Required for normal axon migration across the brain midline and normal formation of the corpus callosum. Protects motoneurons against apoptosis; protection against apoptosis is probably mediated via interaction with MAG. Acts in conjunction with RTN4 and LINGO1 in regulating neuronal precursor cell motility during cortical development. Like other family members, plays a role in restricting the number dendritic spines and the number of synapses that are formed during brain development (PubMed:22325200). | T2DM | No |

### About

Table content:

- **Description (PHAROS)**: Full-text description of
  the role/function of the protein from PHAROS.
- **Diabetes associations (Open Targets)**:
  Gene-disease (diabetes mellitus) association through experiments or
  clinical trials. Direct associations with T1DM, T2DM and GDM are shown
  specifically, while all other diabetes mellitus diseases are included in
  “Other”.
- **Suggested biomarker**: Known and suggested
  biomarkers from Abbasi
  et al, 2016, RHAPSODY partners (collected by the RHAPSODY biomarker
  taskforce) and based on PPI-network analysis of known biomarkers
  (in-house analysis).

*“Description missing”* indicates that no description was
available for the target.

# 2 Experimental evidence

## 2.1 Volcano plots exploring p-value and effect size relationships

### 2.1.1 All experimental results for each hit

#### Plot

Volcano plot - all experiments

#### About

Individual volcano plots for each target with results from all
RHAPSODY experiments plotted.

- Shape: Points are shaped according to omics type.
- Color: Points with an adjusted p-value <0.05 are shown in blue,
  while non-significant points are shown in gray.

Empty plots indicates that the specific target were not measured in
RHAPSODY experiments.

### 2.1.2 Compare best experimental result for each target based on p-value

#### Plot

Volcano plot - Best results from each target

#### Table

Best experimental results for each target by omics-type

|  | Experiment | Tissue | Comparison | log10 fold change | Adj. p-value |
| --- | --- | --- | --- | --- | --- |
| **Proteomics** | | | | | |
| GDF15 | WP3 GoDARTS + DCS | Blood | Cox model 1 | 0.424 | 0.030 |
| CRELD1 | WP3 GoDARTS + DCS | Blood | Cox model 3 | 0.382 | 0.159 |
| ENPP7 | WP3 GoDARTS + DCS | Blood | Cox model 3 | 0.357 | 0.159 |
| FAS | WP3 GoDARTS + DCS | Blood | Cox model 3 | 0.315 | 0.159 |
| IL18R1 | WP3 GoDARTS + DCS | Blood | Cox model 1 | 0.401 | 0.190 |
| RTN4R | WP3 GoDARTS + DCS | Blood | Cox model 3 | 0.273 | 0.430 |
| GPT | WP3 GoDARTS + DCS | Blood | Cox model 3 | 0.069 | 0.967 |
| CRP | WP3 GoDARTS + DCS | Blood | Cox model 1 | -0.084 | 0.985 |
| **Transcriptomics** | | | | | |
| CRELD1 | IMIDIA | Islets | T2D vs ND | -0.450 | 1.73e-05 |
| GPT | WP6 Mice | Liver | DBA2J D33 HF vs RC | -0.795 | 8.97e-05 |
| FAS | IMIDIA | Islets | T2D vs ND | 0.514 | 1.42e-04 |
| CRP | IMIDIA | Islets | T2D vs ND | 1.044 | 0.005 |
| IL18R1 | WP5 | Islets LCM | T2D vs ND + Tubingen | 1.300 | 0.058 |
| GDF15 | WP6 Mice | Liver | BALBC D05 HF vs RC | -2.114 | 0.077 |
| RTN4R | WP6 Mice | Adipose tissue | BALBC D33 HF vs RC | 1.962 | 0.126 |
| ENPP7 | IMIDIA | Islets | IGT vs ND | 0.149 | 0.363 |
| GGT1 | IMIDIA | Islets | T2D vs ND | 0.331 | 0.375 |

#### About

Omics-specific volcano plots with the best result for each tissue
based on lowest adjusted p-value. Best results (lowest adjusted p-value)
for all targets is shown as background points.

- Shape: Points are shaped according to omics type.
- Color: Points with an adjusted p-value <0.05 are shown in blue,
  while non-significant points are shown in gray.
- Background: The best results for all targets detected in RHAPSODY
  experiments are included as a reference background in light gray with a
  reduced size.

The best results from each target is shown in a table format together
with the experiment-specific information, including:

- **Experiment**: Experimental setting within the
  RHAPSODY consortium. Find more information on the experiments here.
- **Tissue**: The tissue the target was detected in. The
  tissue availability is omics dependent:
  - Adipose tissue: Transcriptomics.
  - Blood: Proteomics.
  - Islets: Transcriptomics.
  - Islets LCM (laser capture microdissected): Transcriptomics.
  - Liver: Transcriptomics.
  - Muscles: Transcriptomics.
- **Comparison**: Experimental setup or modeling.
- **log10 fold change**: log10-transformed fold change
  (FC).
- **Adj. p-value**: FDR adjusted p-value.

Significant results with an adjusted p-value <0.05 are shown in
**blue**, while
non-significant points are shown in **gray**.

## 2.2 RHAPSODY meta rank

### Table

RHAPSODY meta rank

|  | Detected in blood | Adj. meta p-value | Rank |
| --- | --- | --- | --- |
| ENPP7 | Yes | 1.42e-06 | 20 |
| CRELD1 | Yes | 0.008 | 43 |
| IL18R1 | Yes | 4.82e-08 | 94 |
| RTN4R | Yes | 0.002 | 269 |
| GDF15 | Yes | 2.22e-08 | 355 |
| FAS | Yes | 3.76e-13 | 538 |
| GGT1 | Yes | 0.371 | - |
| GPT | Yes | 0.734 | - |
| CRP | Yes | 0.986 | - |

### About

The RHAPSODY meta rank is made to identify novel targets based on
RHAPSODY experimental data. The rank is based on three steps:

1. Meta-analysis of p-values from all proteomics and transcriptomics
   experiments within the RHAPSODY consortium (see the individual results
   above). The meta-analysis use the sample size-based meta-analysis
   described by Willer et al,
   2010. The reported meta p-values are FDR-adjusted.
2. Targets were evaluated on being found in the blood circulation using
   data from the Human Protein Atlas. Targets found in circulation were
   included in step 3.
3. Targets with a significant meta p-value, defined as meta p-value
   < 0.05, were inversely ranked according to their co-mentions with
   diabetes. The best rank of 1 was assigned to the lowest number of
   co-mentions (zero (0) co-mentions), signifying the most novel biomarker
   for diabetes.

Significant results with an adjusted meta p-value <0.05 are shown
in blue,
while non-significant points are shown in gray.

en-dash (-) indicates that no information was available for the
target.

## 2.3 Best results within tissue and model

### Compare across targets

Compare tissue-specific results across targets

### Compare across species

Compare tissue-specific results across species

### Rank list table

Best result for each target based on tissue type

|  | | Human | | Mouse | |
| --- | --- | --- | --- | --- | --- |
|  | Omics type | log10 fold change | Adj. p-value | log10 fold change | Adj. p-value |
| **Adipose tissue** | | | | | |
| CRELD1 | Transcriptomics | – | – | -1.369 | 0.084 |
| GPT | Transcriptomics | – | – | -1.371 | 0.120 |
| IL18R1 | Transcriptomics | – | – | 1.371 | 0.124 |
| RTN4R | Transcriptomics | – | – | 1.962 | 0.126 |
| FAS | Transcriptomics | – | – | 0.399 | 0.136 |
| ENPP7 | Transcriptomics | – | – | 2.279 | 0.654 |
| **Blood** | | | | | |
| GDF15 | Proteomics | 0.424 | 0.030 | – | – |
| CRELD1 | Proteomics | 0.382 | 0.159 | – | – |
| ENPP7 | Proteomics | 0.357 | 0.159 | – | – |
| FAS | Proteomics | 0.315 | 0.159 | – | – |
| IL18R1 | Proteomics | 0.401 | 0.190 | – | – |
| RTN4R | Proteomics | 0.273 | 0.430 | – | – |
| GPT | Proteomics | 0.069 | 0.967 | – | – |
| CRP | Proteomics | -0.084 | 0.985 | – | – |
| **Islets** | | | | | |
| CRELD1 | Transcriptomics | -0.450 | 1.73e-05 | 0.206 | 0.212 |
| FAS | Transcriptomics | 0.514 | 1.42e-04 | -0.418 | 0.402 |
| CRP | Transcriptomics | 1.044 | 0.005 | -1.267 | 0.103 |
| GDF15 | Transcriptomics | 0.333 | 0.149 | – | – |
| IL18R1 | Transcriptomics | 0.238 | 0.241 | – | – |
| ENPP7 | Transcriptomics | 0.149 | 0.363 | – | – |
| GGT1 | Transcriptomics | 0.331 | 0.375 | – | – |
| GPT | Transcriptomics | -0.067 | 0.440 | 0.417 | 0.152 |
| **Islets LCM** | | | | | |
| FAS | Transcriptomics | 1.092 | 0.010 | – | – |
| IL18R1 | Transcriptomics | 1.300 | 0.058 | – | – |
| CRP | Transcriptomics | 1.451 | 0.204 | – | – |
| GDF15 | Transcriptomics | 0.683 | 0.394 | – | – |
| GGT1 | Transcriptomics | 0.637 | 0.420 | – | – |
| CRELD1 | Transcriptomics | -0.129 | 0.476 | – | – |
| **Liver** | | | | | |
| GPT | Transcriptomics | – | – | -0.795 | 8.97e-05 |
| FAS | Transcriptomics | – | – | 0.737 | 0.006 |
| CRELD1 | Transcriptomics | – | – | -0.532 | 0.026 |
| GDF15 | Transcriptomics | – | – | -2.114 | 0.077 |
| CRP | Transcriptomics | – | – | 0.357 | 0.114 |
| **Muscle** | | | | | |
| FAS | Transcriptomics | – | – | -1.179 | 0.003 |
| GPT | Transcriptomics | – | – | 0.494 | 0.028 |
| CRELD1 | Transcriptomics | – | – | -0.311 | 0.032 |
| CRP | Transcriptomics | – | – | -2.606 | 0.516 |

### About

#### Compare across targets

Comparison of targets across tissues and species. The plot is made to
enable direct comparison between targets. Dependent on the number of
targets, the plot will give target comparisons horizontally (at 8 or
fewer targets) or vertically (more than 8 targets).

- Shape: Points are shaped according to omics type.
- Color: Points with an adjusted p-value <0.05 are shown in blue,
  while non-significant points are shown in gray.
- Color density: Points with a adjusted p-value <0.05 are shown at
  full density, while points above the p-value threshold are more
  see-through (lower density).

Empty plots indicates that the specific target were not measured in
RHAPSODY experiments.

#### Compare across species

Comparison of tissues across species for each target. The plot is
made to enable direct comparison between tissues and species for each
target.

- Shape: Points are shaped according to omics type.
- Color: Points with an adjusted p-value <0.05 are shown in blue,
  while non-significant points are shown in gray.
- Color density: Points with a adjusted p-value <0.05 are shown at
  full density, while points above the p-value threshold are more
  see-through (lower density).

Empty plots indicates that the specific target were not measured in
RHAPSODY experiments.

#### Rank list table

The table summarizes the best results for each tissue and species
based on adjusted p-value.

- **Omics type**: Omics type
- **log10 fold change**: log10-transformed fold change
  (FC).
- **Adj. p-value**: FDR adjusted p-value.

Significant results with an adjusted p-value <0.05 are shown in
blue,
while non-significant points are shown in gray.

en-dash (-) indicates that no information was available for the
target.

# 3 Disease association

## 3.1 Text mining results from public and in-house sources

### 3.1.1 Disease association in scientific articles

#### Plot

Disease association in scientific articles

#### About

Text mining results for target-disease (any) association based on
in-house and PHAROS data.

- In-house text-mining
  - UniProt AC - all diseases co-mentions (PubMed): Sum of diabetes and
    all other diseases co-mentions in PubMed abstracts.
  - UniProt AC - all diseases co-mentions (full-text): Sum of diabetes
    and all other diseases co-mentions in 15 mio full-text articles.
- PHAROS
  - Jensen Score: Gene-disease association score based on biomedical
    abstracts, taking co-occurrence within and between sentences into
    account. Absolute counts are reported here. For more information, see Pletscher-Frankild
    et al, 2015.
  - PubTator Score: Online tool for curation of PubMed articles.
    Biological entities (chemicals, diseases, genes, mutations, and species)
    are annotated using entity-specific text-mining tools. Normalized scores
    used for ranking in the tool is reported here. For more information, see
    the website
    and Wei,
    2013.

To ease the comparison between the proteins, a geometric mean has
been calculated for the five parameters. Geometric means can be
calculated with the formula: \(GM =
\sqrt[n]{x\_{1} \cdot x\_{2} \cdot ... \cdot x\_{n}}\).  Using the
geometric mean instead of the arithmetic mean (normally known as mean or
average) to summarize our data is beneficial for two reasons:

- The geometric mean is not affected by the scale of the data. We can
  therefore summarize our data without normalizing it.
- The geometric mean is less sensitive to outliers than the arithmetic
  mean, but still more sensitive than the median.
- The geometric mean is also more stable than the median, which can
  change dramatically when adding or removing just a few input values if
  your sample size is small.

However, it is important to note that the geometric mean **does
not** handle zeros and negative values. It is therefore not
useful for all data types.

### 3.1.2 Diabetes fraction of co-mentions

#### Plot

Disease co-mentions

#### About

The in-house text-mining has been conducted in two modes for PubMed
abstracts and full-text papers:

- UniProt AC - all diseases except diabetes co-mentions
- UniProt AC - diabetes co-mentions

Using these text-mining results, we can compare the number of
co-mentions associated with diabetes to all other diseases.

The plot is split into three panel (left-to-right):

- A proportional area plot (left panel) shows the percentage of
  co-mentions in full text articles that are diabetes-related.
- The first bar plot (middle panel) shows the co-mentions count for
  diabetes in dark
  blue and the co-mentions count for all diseases *but*
  diabetes in light
  blue derived from 15 million full-text articles (in-house
  resource, see Westergaard
  et al. PLoS Computational Biology 2018).
- The second bar plot (right panel) shows similar to the middle panel
  co-mentions count for diabetes in dark
  blue and the co-mentions count for all diseases *but*
  diabetes in light
  blue. The data is obtained from 15 million abstracts (in-house
  resource, see Westergaard et
  al. PLoS Computational Biology 2018).

The plot has been sorted according to highest full-text all diabetes
co-mentions.

## 3.2 Open Targets association scores

### Diabetes mellitus (EFO\_000400)

Association score for diabetes mellitus (supergroup)

### T2DM (MONDO\_0005148)

Association score for T2DM

### T1DM (MONDO\_0005147)

Association score for T1DM

### GDM (EFO\_0004593)

Association score for GDM

### Other diabetes mellitus subtypes

Association score for other diabetes mellitus subtypes

### About

Open Targets association scores for the diabetes mellitus supergroup
and selected subtypes.

- **Diabetes mellitus (EFO\_0000400)**: Supergroup for
  all diabetes mellitus diseases.
- **T2DM (MONDO\_0005148)**: Type 2 diabetes
  mellitus.
- **T1DM (MONDO\_0005147)**: Type 1 diabetes
  mellitus.
- **GDM (EFO\_0004593)**: Gestational diabetes
  mellitus.
- **Other diabetes mellitus subtypes**: Any other
  diabetes mellitus subgroup than T2DM, T1DM and GDM, e.g., monogenic
  diabetes mellitus, prediabetes syndrome, and neonatal diabetes
  mellitus.

The Open Targets association score is a standardized measure reported
a value between 0 and 1. Here, 1 indicates a strong association, while 0
indicates that there is no known association.  The score is calculated
with the formula \(s = F \cdot S \cdot
C\), where \(s\) is the score,
\(F\) is the frequency i.e. the
relative occurrence of a target-disease evidence, \(S\) is the severity i.e. the magnitude or
strength of the effect described by the evidence, and \(C\) is the confidence i.e. overall
confidence for the observation that generates the target-disease
evidence.  
The targets are ranked according to the overall association score,
summarizing the seven association variables.

Source: Open
Targets Platform Documentation.

# 4 Mechanistic evidence

## 4.1 Protein-protein interaction network

### 4.1.1 Network plots

#### All interactions

PPI networks

#### High-confidence interactions only

PPI networks

#### Summary table

Protein-protein interaction networks summary

| Target | Mean diabetes textmining % | Associated with diabetes | Suggested diabetes biomarkers | Potential interaction partners | High-confidence interaction partners | Total interaction partners |
| --- | --- | --- | --- | --- | --- | --- |
| CRELD1 | - | 0 | 0 | 0 | 0 | 0 |
| CRP | 12 | 47 | 11 | 55 | 13 | 68 |
| ENPP7 | 15 | 9 | 0 | 20 | 0 | 20 |
| FAS | 10 | 71 | 0 | 96 | 21 | 117 |
| GDF15 | 10 | 45 | 2 | 67 | 2 | 69 |
| GGT1 | 14 | 23 | 3 | 42 | 0 | 42 |
| GPT | 12 | 48 | 1 | 75 | 0 | 75 |
| IL18R1 | 13 | 8 | 1 | 8 | 4 | 12 |
| RTN4R | 9 | 76 | 1 | 191 | 10 | 201 |

#### About

Protein-protein interaction networks based on physical links data
from the STRING database. See Szklarczyk
et al, 2018 for a description of the resource.

Network features:

- Interaction partners (nodes) are colored according to the confidence
  score of their interaction with the primary targets.
  - High confidence interaction partners (confidence score > 0.7) are
    shown in blue
  - Suggested interaction partners (confidence score ≤ 0.7) are shown in
    gray
- Interactions (edges) are shown according the confidence score of the
  interaction
  - High confidence interactions (confidence score > 0.7) are shown
    as solid line (—)
  - Suggested interactions (confidence score ≤ 0.7) are show as dashed
    lines (- - -)

Summary table:

- **Mean diabetes textmining %**: The avarage diabetes
  percentage of diabetes co-mentions to total co-mentions across the
  network. Based on textmining from Westergaard et al
- **Associated with diabetes**: Number of nodes that have
  been associated with diabetes mellitus (Open Targets)
- **Suggested diabetes biomarkers**: Number of nodes that
  have been identified as potential biomarkers for Type 2 diabetes
  mellitus
- **Potential interaction partners**: Number of
  interaction partners below the confidence threshold (confidence score ≤
  0.7)
- **High-confidence interaction partners**: Number of
  interaction partners above the confidence threshold (confidence score
  > 0.7)
- **Total interaction partners**: Total number of
  interaction partners

In cases where none of the targets have any interaction partners (all
or high-confident), the following error messages will be shown:

- All interactions:
  `Unfortunately, no interactions were found for any of the selected targets.`
- High-confidence interactions:
  `Unfortunately, no high-confidence interactions were found for any of the selected targets.`

### 4.1.2 Interaction partners

#### All interactions

Protein-protein interaction partners

|  | | Diabetes association | | | Confidence scores for interaction | | | |
| --- | --- | --- | --- | --- | --- | --- | --- | --- |
| Interaction partner | UniProt ID | Diabetes textmining % | Diabetes associations | Suggested diabetes biomarker | Experimental score | Database score | Textmining score | Combined score |
| **CRP** | | | | | | | | |
| RBP4 | P02753 | 38 | T1DM, T2DM, GDM, Other | Abbasi et al, 2016 | 0.00 | 0.00 | 0.19 | 0.19 |
| FFAR2 | O15552 | 36 | T1DM, T2DM, Other | No | 0.27 | 0.00 | 0.05 | 0.28 |
| APOB | P04114 | 34 | T1DM, T2DM, GDM, Other | Abbasi et al, 2016 | 0.00 | 0.00 | 0.35 | 0.34 |
| ANGPTL4 | Q9BY76 | 27 | T1DM, T2DM, GDM, Other | No | 0.00 | 0.00 | 0.21 | 0.21 |
| OLR1 | P78380 | 26 | T2DM, GDM | RHAPSODY partner suggestion | 0.00 | 0.00 | 0.88 | 0.88 |
| SELP | P16109 | 22 | T1DM, T2DM, GDM, Other | No | 0.00 | 0.00 | 0.33 | 0.33 |
| LEP | P41159 | 21 | T1DM, T2DM, GDM, Other | Abbasi et al, 2016 | 0.00 | 0.00 | 0.60 | 0.60 |
| FCGR1B | Q92637 | 19 | – | No | 0.00 | 0.00 | 0.53 | 0.53 |
| DDIT3 | P35638 | 18 | T1DM, T2DM, GDM, Other | No | 0.00 | 0.00 | 0.41 | 0.41 |
| SNRPN | P63162 | 16 | – | No | 0.00 | 0.00 | 0.25 | 0.25 |
| TLR4 | O00206 | 16 | T1DM, T2DM, GDM, Other | No | 0.00 | 0.00 | 0.15 | 0.15 |
| SNRNP70 | P08621 | 15 | – | No | 0.27 | 0.00 | 0.00 | 0.27 |
| CFHR5 | Q9BXR6 | 14 | – | No | 0.30 | 0.00 | 0.59 | 0.70 |
| C1QC | P02747 | 14 | – | No | 0.00 | 0.60 | 0.26 | 0.69 |
| APCS | P02743 | 14 | T1DM, GDM | No | 0.46 | 0.00 | 0.41 | 0.67 |
| PTX3 | P26022 | 14 | T1DM, T2DM, GDM, Other | RHAPSODY partner suggestion | 0.30 | 0.00 | 0.00 | 0.30 |
| C1QA | P02745 | 13 | – | No | 0.57 | 0.60 | 0.26 | 0.86 |
| C1QB | P02746 | 13 | – | No | 0.00 | 0.60 | 0.00 | 0.60 |
| CD14 | P08571 | 13 | T1DM | No | 0.00 | 0.00 | 0.25 | 0.25 |
| FCN3 | O75636 | 13 | T1DM, T2DM, GDM, Other | No | 0.00 | 0.00 | 0.16 | 0.16 |
| CFHR3 | Q02985 | 12 | – | No | 0.42 | 0.00 | 0.00 | 0.42 |
| LOX | P28300 | 12 | T1DM, T2DM, Other | No | 0.00 | 0.00 | 0.31 | 0.31 |
| LAMA1 | P25391 | 12 | T2DM, Other | Network of genes in T2D loci (suggested candidate) | 0.29 | 0.00 | 0.00 | 0.28 |
| GP1BA | P07359 | 12 | T2DM, Other | No | 0.00 | 0.00 | 0.25 | 0.24 |
| CES1 | P23141 | 12 | T1DM, T2DM, Other | No | 0.06 | 0.00 | 0.21 | 0.22 |
| PTPRS | Q13332 | 12 | T1DM | No | 0.19 | 0.00 | 0.00 | 0.19 |
| PRSS23 | O95084 | 12 | T1DM | No | 0.18 | 0.00 | 0.00 | 0.18 |
| CTSH | P09668 | 12 | T1DM, T2DM | No | 0.00 | 0.00 | 0.17 | 0.17 |
| FCN2 | Q15485 | 11 | T1DM | Network expansion | 0.27 | 0.00 | 0.83 | 0.87 |
| C4A | P0C0L4 | 11 | T1DM, T2DM, GDM, Other | No | 0.00 | 0.00 | 0.72 | 0.72 |
| C1GALT1 | Q9NS00 | 11 | Other | No | 0.26 | 0.00 | 0.00 | 0.26 |
| CD53 | P19397 | 11 | – | No | 0.00 | 0.00 | 0.16 | 0.15 |
| CFH | P08603 | 10 | T1DM, T2DM, GDM | Network expansion | 0.73 | 0.00 | 0.90 | 0.97 |
| FCGR2A | P12318 | 10 | T1DM, T2DM, Other | No | 0.46 | 0.00 | 0.90 | 0.94 |
| C3 | P01024 | 10 | T1DM, T2DM, GDM, Other | Abbasi et al, 2016 | 0.48 | 0.00 | 0.66 | 0.82 |
| FCGR2B | P31994 | 10 | T2DM, Other | No | 0.00 | 0.00 | 0.75 | 0.75 |
| FCGR3A | P08637 | 10 | T1DM | No | 0.00 | 0.00 | 0.27 | 0.27 |
| CALR | P27797 | 10 | T1DM, T2DM | No | 0.00 | 0.00 | 0.23 | 0.23 |
| UBR3 | Q6ZT12 | 10 | T1DM | No | 0.19 | 0.00 | 0.00 | 0.19 |
| C4BPA | P04003 | 9 | – | No | 0.42 | 0.00 | 0.54 | 0.72 |
| CFHR1 | Q03591 | 9 | T2DM | No | 0.30 | 0.00 | 0.41 | 0.56 |
| FCN1 | O00602 | 8 | T1DM | No | 0.30 | 0.00 | 0.86 | 0.90 |
| H2AC20 | Q16777 | 8 | – | No | 0.27 | 0.00 | 0.35 | 0.51 |
| CFP | P27918 | 8 | T1DM, T2DM | Network expansion | 0.30 | 0.00 | 0.25 | 0.45 |
| GMPPA | Q96IJ6 | 8 | Other | No | 0.28 | 0.00 | 0.00 | 0.28 |
| CFI | P05156 | 8 | T1DM, T2DM, Other | No | 0.27 | 0.00 | 0.00 | 0.27 |
| ST6GAL1 | P15907 | 8 | T1DM, T2DM | No | 0.00 | 0.00 | 0.27 | 0.27 |
| CD209 | Q9NNX6 | 8 | T1DM, GDM | No | 0.00 | 0.00 | 0.26 | 0.26 |
| CHST3 | Q7LGC8 | 8 | T1DM, T2DM | No | 0.18 | 0.00 | 0.00 | 0.18 |
| CNTNAP3 | Q9BZ76 | 8 | – | No | 0.18 | 0.00 | 0.00 | 0.18 |
| H1-0 | P07305 | 7 | Other | No | 0.00 | 0.00 | 0.29 | 0.29 |
| KRT8 | P05787 | 7 | GDM | No | 0.00 | 0.00 | 0.28 | 0.28 |
| H1-1 | Q02539 | 7 | T1DM | No | 0.27 | 0.00 | 0.00 | 0.27 |
| STC2 | O76061 | 7 | T2DM, Other | No | 0.23 | 0.00 | 0.00 | 0.23 |
| H2AW | Q7L7L0 | 7 | – | No | 0.00 | 0.00 | 0.21 | 0.21 |
| MAPK8IP2 | Q13387 | 7 | – | No | 0.17 | 0.00 | 0.00 | 0.17 |
| CFHR4 | Q92496 | 6 | – | No | 0.57 | 0.00 | 0.76 | 0.89 |
| C1S | P09871 | 6 | – | No | 0.00 | 0.00 | 0.18 | 0.18 |
| LGALS3BP | Q08380 | 5 | T1DM, T2DM | No | 0.00 | 0.00 | 0.21 | 0.21 |
| ITM2B | Q9Y287 | 4 | T1DM, T2DM, Other | No | 0.18 | 0.00 | 0.00 | 0.17 |
| CA11 | O75493 | - | – | No | 0.18 | 0.00 | 0.00 | 0.18 |
| FCGR1A | P12314 | - | – | No | 0.00 | 0.00 | 0.94 | 0.94 |
| FCAR | P24071 | - | – | No | 0.00 | 0.00 | 0.44 | 0.44 |
| C4B | P0C0L5 | - | T1DM | No | 0.00 | 0.00 | 0.31 | 0.31 |
| C4B\_2 | P0C0L5 | - | T1DM | No | 0.00 | 0.00 | 0.31 | 0.31 |
| GP6 | Q9HCN6 | - | T1DM, T2DM | No | 0.00 | 0.00 | 0.27 | 0.27 |
| S100A12 | P80511 | - | T2DM, Other | RHAPSODY partner suggestion | 0.00 | 0.00 | 0.18 | 0.18 |
| **ENPP7** | | | | | | | | |
| TTC17 | Q96AE7 | 29 | – | No | 0.13 | 0.00 | 0.26 | 0.33 |
| ACER2 | Q5QJU3 | 22 | – | No | 0.00 | 0.65 | 0.00 | 0.65 |
| ASAH2 | Q9NR71 | 19 | T2DM | No | 0.00 | 0.65 | 0.00 | 0.65 |
| CERS6 | Q6ZMG9 | 19 | T2DM | No | 0.00 | 0.65 | 0.00 | 0.65 |
| PIGF | Q07326 | 18 | – | No | 0.08 | 0.00 | 0.14 | 0.18 |
| CERS4 | Q9HA82 | 17 | T2DM | No | 0.00 | 0.65 | 0.00 | 0.65 |
| SGPP1 | Q9BX95 | 17 | T2DM | No | 0.00 | 0.65 | 0.00 | 0.65 |
| CERS2 | Q96G23 | 17 | T1DM, T2DM | No | 0.00 | 0.65 | 0.00 | 0.65 |
| ACER1 | Q8TDN7 | 16 | – | No | 0.00 | 0.65 | 0.00 | 0.65 |
| CERS3 | Q8IU89 | 16 | – | No | 0.00 | 0.65 | 0.00 | 0.65 |
| PLPP3 | O14495 | 15 | – | No | 0.00 | 0.65 | 0.00 | 0.65 |
| SGPP2 | Q8IWX5 | 14 | Other | No | 0.00 | 0.65 | 0.00 | 0.65 |
| PLPP2 | O43688 | 14 | – | No | 0.00 | 0.65 | 0.00 | 0.65 |
| ASAH1 | Q13510 | 13 | T1DM, T2DM, GDM, Other | No | 0.00 | 0.65 | 0.00 | 0.65 |
| GBA2 | Q9HCG7 | 11 | T2DM | No | 0.00 | 0.65 | 0.00 | 0.65 |
| DEGS2 | Q6QHC5 | 11 | – | No | 0.00 | 0.65 | 0.00 | 0.65 |
| DEGS1 | O15121 | 10 | T1DM, T2DM | No | 0.00 | 0.65 | 0.00 | 0.65 |
| GBA | P04062 | 9 | – | No | 0.00 | 0.65 | 0.00 | 0.65 |
| LRRC73 | Q5JTD7 | - | – | No | 0.26 | 0.00 | 0.00 | 0.26 |
| CERS5 | Q8N5B7 | - | – | No | 0.00 | 0.65 | 0.00 | 0.65 |
| **FAS** | | | | | | | | |
| SFXN5 | Q8TD22 | 23 | – | No | 0.22 | 0.00 | 0.00 | 0.22 |
| TNFRSF10A | O00220 | 22 | T1DM, T2DM, GDM | No | 0.00 | 0.60 | 0.42 | 0.76 |
| GGCX | P38435 | 22 | – | No | 0.00 | 0.00 | 0.34 | 0.34 |
| UFSP2 | Q9NUQ7 | 22 | – | No | 0.22 | 0.00 | 0.00 | 0.22 |
| SPATA5L1 | Q9BVQ7 | 22 | – | No | 0.21 | 0.00 | 0.00 | 0.21 |
| ACAD9 | Q9H845 | 21 | – | No | 0.16 | 0.00 | 0.00 | 0.16 |
| NDUFAF1 | Q9Y375 | 20 | T1DM, T2DM, GDM, Other | No | 0.19 | 0.00 | 0.00 | 0.19 |
| PEX1 | O43933 | 18 | Other | No | 0.27 | 0.00 | 0.00 | 0.27 |
| MAP3K5 | Q99683 | 17 | T1DM, T2DM | No | 0.64 | 0.90 | 0.00 | 0.96 |
| PEA15 | Q15121 | 17 | T2DM | No | 0.28 | 0.00 | 0.00 | 0.28 |
| HNF4A | P41235 | 17 | T1DM, T2DM, GDM, Other | No | 0.00 | 0.00 | 0.26 | 0.26 |
| PDXDC1 | Q6P996 | 17 | T2DM | No | 0.22 | 0.00 | 0.00 | 0.22 |
| CAV1 | Q03135 | 16 | T1DM, T2DM, GDM, Other | No | 0.48 | 0.00 | 0.46 | 0.71 |
| HSPA5 | P11021 | 16 | T1DM, T2DM, GDM, Other | No | 0.27 | 0.00 | 0.00 | 0.27 |
| MAPK8 | P45983 | 15 | T1DM, T2DM, GDM, Other | No | 0.64 | 0.00 | 0.00 | 0.64 |
| TRAF2 | Q12933 | 15 | – | No | 0.00 | 0.60 | 0.00 | 0.60 |
| LGALS3 | P17931 | 15 | T1DM, T2DM, GDM, Other | No | 0.00 | 0.00 | 0.33 | 0.33 |
| PIK3R1 | P27986 | 15 | T1DM, T2DM, GDM, Other | No | 0.27 | 0.00 | 0.00 | 0.27 |
| ATG5 | Q9H1Y0 | 14 | T2DM, Other | No | 0.48 | 0.00 | 0.25 | 0.59 |
| CD40 | P25942 | 14 | T1DM, T2DM, GDM, Other | No | 0.00 | 0.00 | 0.53 | 0.53 |
| TNFSF13 | O75888 | 14 | – | No | 0.27 | 0.00 | 0.00 | 0.27 |
| LTA | P01374 | 14 | T1DM, T2DM, GDM, Other | No | 0.06 | 0.00 | 0.19 | 0.20 |
| C1QC | P02747 | 14 | – | No | 0.00 | 0.00 | 0.18 | 0.18 |
| TNFRSF1A | P19438 | 13 | T1DM, T2DM, GDM, Other | No | 0.29 | 0.00 | 0.40 | 0.55 |
| WDR11 | Q9BZH6 | 13 | T2DM, Other | No | 0.42 | 0.00 | 0.00 | 0.42 |
| MAP1LC3B | Q9GZQ8 | 13 | T2DM | No | 0.27 | 0.00 | 0.00 | 0.27 |
| PSTPIP1 | O43586 | 13 | T2DM | No | 0.00 | 0.00 | 0.25 | 0.25 |
| C1QA | P02745 | 13 | – | No | 0.00 | 0.00 | 0.18 | 0.18 |
| FASLG | P48023 | 12 | T1DM, T2DM, Other | No | 0.65 | 0.90 | 0.90 | 1.00 |
| FAF1 | Q9UNN5 | 12 | T2DM | No | 0.50 | 0.90 | 0.45 | 0.97 |
| SQSTM1 | Q13501 | 12 | T1DM, T2DM, Other | No | 0.27 | 0.00 | 0.00 | 0.27 |
| ANK3 | Q12955 | 12 | T1DM | No | 0.21 | 0.00 | 0.00 | 0.21 |
| NRP1 | O14786 | 12 | T1DM, T2DM | No | 0.00 | 0.00 | 0.16 | 0.16 |
| TNFRSF10B | O14763 | 11 | T1DM, T2DM | No | 0.29 | 0.60 | 0.00 | 0.70 |
| PLCG1 | P19174 | 11 | T2DM | No | 0.27 | 0.00 | 0.61 | 0.70 |
| RHOA | P61586 | 11 | T1DM, T2DM, Other | No | 0.65 | 0.00 | 0.00 | 0.65 |
| HIPK3 | Q9H422 | 11 | T2DM, Other | No | 0.34 | 0.00 | 0.28 | 0.50 |
| TRAF3 | Q13114 | 11 | T2DM | No | 0.27 | 0.00 | 0.00 | 0.27 |
| CANX | P27824 | 11 | – | No | 0.00 | 0.00 | 0.25 | 0.25 |
| ARF5 | P84085 | 11 | T2DM | No | 0.24 | 0.00 | 0.00 | 0.24 |
| GPD1L | Q8N335 | 11 | – | No | 0.20 | 0.00 | 0.00 | 0.20 |
| UNC5B | Q8IZJ1 | 11 | GDM | No | 0.00 | 0.00 | 0.18 | 0.18 |
| RIPK1 | Q13546 | 10 | – | No | 0.44 | 0.90 | 0.47 | 0.97 |
| CASP10 | Q92851 | 10 | – | No | 0.52 | 0.90 | 0.47 | 0.97 |
| FAIM2 | Q9BWQ8 | 10 | T2DM, GDM | No | 0.27 | 0.90 | 0.00 | 0.92 |
| PTPN13 | Q12923 | 10 | – | No | 0.48 | 0.00 | 0.41 | 0.68 |
| TNFSF10 | P50591 | 10 | T1DM, T2DM | No | 0.00 | 0.60 | 0.00 | 0.60 |
| FYN | P06241 | 10 | T2DM, GDM | No | 0.50 | 0.00 | 0.00 | 0.50 |
| LCK | P06239 | 10 | T1DM | No | 0.48 | 0.00 | 0.00 | 0.48 |
| HSPA8 | P11142 | 10 | – | No | 0.00 | 0.00 | 0.43 | 0.43 |
| CTNNB1 | P35222 | 10 | T1DM, T2DM, GDM | No | 0.39 | 0.00 | 0.00 | 0.39 |
| CASP3 | P42574 | 10 | T1DM, T2DM, GDM, Other | No | 0.06 | 0.36 | 0.00 | 0.37 |
| TUBB4A | P04350 | 10 | Other | No | 0.28 | 0.00 | 0.00 | 0.28 |
| HSPA4 | P34932 | 10 | – | No | 0.00 | 0.00 | 0.27 | 0.27 |
| EIF2AK3 | Q9NZJ5 | 10 | T1DM, T2DM, GDM, Other | No | 0.27 | 0.00 | 0.00 | 0.27 |
| FCGR2B | P31994 | 10 | T2DM, Other | No | 0.00 | 0.00 | 0.23 | 0.23 |
| CDYL | Q9Y232 | 10 | – | No | 0.22 | 0.00 | 0.00 | 0.22 |
| TUBB2B | Q9BVA1 | 10 | – | No | 0.21 | 0.00 | 0.00 | 0.21 |
| UGDH | O60701 | 10 | – | No | 0.19 | 0.00 | 0.00 | 0.19 |
| CFLAR | O15519 | 9 | T2DM | No | 0.49 | 0.90 | 0.45 | 0.97 |
| SRC | P12931 | 9 | T1DM, T2DM | No | 0.48 | 0.90 | 0.20 | 0.95 |
| BTK | Q06187 | 9 | – | No | 0.00 | 0.90 | 0.07 | 0.90 |
| SYK | P43405 | 9 | Other | No | 0.00 | 0.90 | 0.00 | 0.90 |
| SUMO1 | P63165 | 9 | T1DM, T2DM | No | 0.48 | 0.00 | 0.61 | 0.79 |
| BID | P55957 | 9 | T1DM | No | 0.65 | 0.00 | 0.00 | 0.65 |
| RAP1A | P62834 | 9 | T2DM, GDM | No | 0.55 | 0.00 | 0.00 | 0.55 |
| UBE2I | P63279 | 9 | T2DM | No | 0.50 | 0.00 | 0.00 | 0.50 |
| HIPK2 | Q9H2X6 | 9 | – | No | 0.10 | 0.00 | 0.41 | 0.45 |
| TRIP6 | Q15654 | 9 | – | No | 0.27 | 0.00 | 0.00 | 0.27 |
| CD44 | P16070 | 9 | T1DM, T2DM, GDM, Other | No | 0.00 | 0.00 | 0.26 | 0.25 |
| TUBB2A | Q13885 | 9 | – | No | 0.21 | 0.00 | 0.00 | 0.21 |
| CASP8 | Q14790 | 8 | T1DM, T2DM | No | 0.53 | 0.90 | 0.94 | 1.00 |
| FADD | Q13158 | 8 | T1DM | No | 0.95 | 0.90 | 0.91 | 1.00 |
| YES1 | P07947 | 8 | – | No | 0.48 | 0.00 | 0.00 | 0.48 |
| BMP1 | P13497 | 8 | – | No | 0.00 | 0.00 | 0.29 | 0.29 |
| APAF1 | O14727 | 8 | – | No | 0.25 | 0.00 | 0.00 | 0.24 |
| RUFY1 | Q96T51 | 8 | T2DM | No | 0.23 | 0.00 | 0.00 | 0.23 |
| EGFR | P00533 | 7 | T1DM, T2DM, GDM, Other | No | 0.00 | 0.00 | 0.75 | 0.75 |
| EZR | P15311 | 7 | T1DM | No | 0.47 | 0.00 | 0.16 | 0.54 |
| FEM1B | Q9UK73 | 7 | T1DM, T2DM, Other | No | 0.21 | 0.00 | 0.28 | 0.41 |
| PML | P29590 | 7 | T1DM, T2DM | No | 0.34 | 0.00 | 0.00 | 0.34 |
| MSN | P26038 | 7 | T2DM | No | 0.30 | 0.00 | 0.00 | 0.30 |
| ARHGDIA | P52565 | 7 | T1DM, Other | No | 0.29 | 0.00 | 0.00 | 0.29 |
| RARA | P10276 | 7 | T2DM | No | 0.27 | 0.00 | 0.00 | 0.27 |
| IFNB1 | P01574 | 7 | T1DM, T2DM, Other | No | 0.00 | 0.00 | 0.26 | 0.26 |
| RHOBTB3 | O94955 | 7 | – | No | 0.22 | 0.00 | 0.00 | 0.22 |
| LYN | P07948 | 7 | T1DM, T2DM, Other | No | 0.00 | 0.00 | 0.22 | 0.22 |
| UBA7 | P41226 | 7 | T2DM | No | 0.21 | 0.00 | 0.00 | 0.21 |
| FAF2 | Q96CS3 | 7 | – | No | 0.20 | 0.00 | 0.00 | 0.20 |
| KRIT1 | O00522 | 6 | – | No | 0.27 | 0.00 | 0.00 | 0.27 |
| TUBB3 | Q13509 | 6 | – | No | 0.24 | 0.00 | 0.00 | 0.24 |
| DPH1 | Q9BZG8 | 6 | – | No | 0.23 | 0.00 | 0.00 | 0.23 |
| RDX | P35241 | 6 | T1DM, T2DM | No | 0.06 | 0.00 | 0.20 | 0.21 |
| FBF1 | Q8TES7 | 6 | Other | No | 0.19 | 0.00 | 0.00 | 0.19 |
| C17orf75 | Q9HAS0 | 5 | – | No | 0.63 | 0.00 | 0.00 | 0.63 |
| PLK3 | Q9H4B4 | 5 | – | No | 0.00 | 0.00 | 0.40 | 0.40 |
| PRAM1 | Q96QH2 | 5 | – | No | 0.27 | 0.00 | 0.00 | 0.27 |
| PDCD6 | O75340 | 5 | – | No | 0.27 | 0.00 | 0.00 | 0.27 |
| PLK1 | P53350 | 5 | T2DM, GDM | No | 0.00 | 0.00 | 0.20 | 0.20 |
| MAP1S | Q66K74 | 5 | – | No | 0.19 | 0.00 | 0.00 | 0.19 |
| INPP5D | Q92835 | 4 | T1DM, T2DM | No | 0.27 | 0.00 | 0.25 | 0.43 |
| TP63 | Q9H3D4 | 4 | – | No | 0.27 | 0.00 | 0.00 | 0.27 |
| EEF1AKNMT | Q8N6R0 | 4 | – | No | 0.21 | 0.00 | 0.00 | 0.21 |
| INTS2 | Q9H0H0 | 4 | – | No | 0.19 | 0.00 | 0.00 | 0.19 |
| BABAM2 | Q9NXR7 | 3 | – | No | 0.27 | 0.00 | 0.00 | 0.27 |
| CTU2 | Q2VPK5 | 3 | – | No | 0.24 | 0.00 | 0.00 | 0.24 |
| FAM91A1 | Q658Y4 | - | – | No | 0.41 | 0.00 | 0.00 | 0.41 |
| FAM118B | Q9BPY3 | - | – | No | 0.23 | 0.00 | 0.00 | 0.23 |
| TIMM29 | Q9BSF4 | - | – | No | 0.23 | 0.00 | 0.00 | 0.23 |
| DAXX | Q9UER7 | - | T1DM | No | 0.66 | 0.90 | 0.39 | 0.98 |
| TRADD | Q15628 | - | – | No | 0.46 | 0.90 | 0.28 | 0.96 |
| CALM1 | P0DP23 | - | T1DM, GDM | No | 0.80 | 0.00 | 0.00 | 0.80 |
| PRKCA | P17252 | - | T1DM, T2DM | No | 0.55 | 0.00 | 0.00 | 0.55 |
| TNF | P01375 | - | T1DM, T2DM, GDM, Other | No | 0.28 | 0.00 | 0.19 | 0.39 |
| ABHD11 | Q8NFV4 | - | Other | No | 0.39 | 0.00 | 0.00 | 0.39 |
| CASP8AP2 | Q9UKL3 | - | – | No | 0.34 | 0.00 | 0.00 | 0.34 |
| CHD1L | Q86WJ1 | - | T2DM | No | 0.21 | 0.00 | 0.00 | 0.21 |
| **GDF15** | | | | | | | | |
| INSR | P06213 | 27 | T1DM, T2DM, GDM, Other | No | 0.15 | 0.00 | 0.08 | 0.18 |
| ERN1 | O75460 | 26 | T1DM, T2DM, GDM, Other | No | 0.15 | 0.00 | 0.07 | 0.17 |
| PRKCZ | Q05513 | 23 | T2DM, GDM | No | 0.15 | 0.00 | 0.05 | 0.16 |
| GSK3A | P49840 | 22 | T1DM, T2DM, GDM, Other | No | 0.15 | 0.00 | 0.06 | 0.16 |
| IGF1R | P08069 | 17 | T1DM, T2DM, GDM, Other | RHAPSODY partner suggestion | 0.15 | 0.00 | 0.08 | 0.18 |
| IRAK3 | Q9Y616 | 16 | T1DM, T2DM | No | 0.15 | 0.00 | 0.05 | 0.16 |
| GSK3B | P49841 | 15 | T1DM, T2DM, GDM, Other | No | 0.15 | 0.00 | 0.08 | 0.18 |
| STAT5A | P42229 | 14 | T1DM, T2DM, GDM | No | 0.25 | 0.00 | 0.00 | 0.25 |
| ACVR1C | Q8NER5 | 14 | T2DM | No | 0.15 | 0.00 | 0.05 | 0.16 |
| PRKCI | P41743 | 14 | – | No | 0.15 | 0.00 | 0.05 | 0.16 |
| SETD4 | Q9NVD3 | 13 | Other | No | 0.26 | 0.00 | 0.00 | 0.26 |
| MAP3K11 | Q16584 | 13 | – | No | 0.15 | 0.00 | 0.08 | 0.18 |
| IRAK4 | Q9NWZ3 | 13 | T2DM | No | 0.15 | 0.00 | 0.05 | 0.16 |
| ACVRL1 | P37023 | 12 | – | No | 0.15 | 0.00 | 0.30 | 0.38 |
| CDC42BPG | Q6DT37 | 12 | – | No | 0.15 | 0.00 | 0.14 | 0.23 |
| CDC42BPB | Q9Y5S2 | 12 | – | No | 0.15 | 0.00 | 0.08 | 0.18 |
| MAP3K21 | Q5TCX8 | 12 | – | No | 0.15 | 0.00 | 0.08 | 0.18 |
| ROCK1 | Q13464 | 12 | T1DM, T2DM | No | 0.15 | 0.00 | 0.08 | 0.18 |
| CCN3 | P48745 | 12 | T2DM | Network expansion | 0.00 | 0.00 | 0.17 | 0.17 |
| ACVR2A | P27037 | 12 | – | No | 0.15 | 0.00 | 0.06 | 0.16 |
| IRAK1 | P51617 | 12 | T1DM, T2DM | No | 0.15 | 0.00 | 0.05 | 0.16 |
| MAP3K9 | P80192 | 11 | – | No | 0.15 | 0.00 | 0.11 | 0.21 |
| ROCK2 | O75116 | 11 | T1DM, T2DM | No | 0.15 | 0.00 | 0.08 | 0.18 |
| MAP3K10 | Q02779 | 11 | – | No | 0.15 | 0.00 | 0.08 | 0.18 |
| COL12A1 | Q99715 | 11 | – | No | 0.08 | 0.00 | 0.14 | 0.17 |
| RIPK3 | Q9Y572 | 11 | T1DM, T2DM | No | 0.15 | 0.00 | 0.07 | 0.17 |
| ACVR1B | P36896 | 11 | – | No | 0.15 | 0.00 | 0.05 | 0.16 |
| LIMK2 | P53671 | 11 | – | No | 0.15 | 0.00 | 0.05 | 0.16 |
| PASK | Q96RG2 | 11 | T2DM, Other | No | 0.15 | 0.00 | 0.04 | 0.15 |
| BMPR1A | P36894 | 10 | T2DM | No | 0.15 | 0.00 | 0.10 | 0.20 |
| AXL | P30530 | 10 | T1DM, T2DM | No | 0.15 | 0.00 | 0.08 | 0.18 |
| IRAK2 | O43187 | 10 | T2DM | No | 0.15 | 0.00 | 0.05 | 0.16 |
| NTRK1 | P04629 | 10 | T1DM, T2DM, GDM, Other | No | 0.15 | 0.00 | 0.05 | 0.16 |
| ERN2 | Q76MJ5 | 10 | – | No | 0.15 | 0.00 | 0.06 | 0.16 |
| TGFBR2 | P37173 | 9 | T1DM, T2DM | No | 0.15 | 0.00 | 0.42 | 0.49 |
| NTRK2 | Q16620 | 9 | T1DM, T2DM, Other | No | 0.15 | 0.00 | 0.08 | 0.18 |
| RIPK2 | O43353 | 9 | T2DM | No | 0.15 | 0.00 | 0.08 | 0.18 |
| BMPR1B | O00238 | 9 | T2DM | No | 0.15 | 0.00 | 0.07 | 0.17 |
| GDNF | P39905 | 9 | T1DM, T2DM, Other | No | 0.00 | 0.00 | 0.16 | 0.16 |
| PTK7 | Q13308 | 9 | – | No | 0.15 | 0.00 | 0.04 | 0.15 |
| LRRK2 | Q5S007 | 9 | T1DM, T2DM, Other | No | 0.15 | 0.00 | 0.05 | 0.15 |
| SAE1 | Q9UBE0 | 8 | T2DM | No | 0.21 | 0.00 | 0.00 | 0.21 |
| CDC42BPA | Q5VT25 | 8 | – | No | 0.15 | 0.00 | 0.08 | 0.18 |
| NTRK3 | Q16288 | 8 | – | No | 0.15 | 0.00 | 0.08 | 0.18 |
| PTK6 | Q13882 | 8 | – | No | 0.15 | 0.00 | 0.06 | 0.17 |
| MET | P08581 | 8 | T1DM, T2DM, GDM, Other | No | 0.15 | 0.00 | 0.05 | 0.16 |
| MERTK | Q12866 | 8 | T2DM, Other | No | 0.15 | 0.00 | 0.06 | 0.16 |
| TWSG1 | Q9GZX9 | 7 | T1DM | No | 0.10 | 0.00 | 0.65 | 0.67 |
| VIM | P08670 | 7 | T1DM, T2DM, Other | No | 0.14 | 0.00 | 0.27 | 0.34 |
| GFRA1 | P56159 | 7 | – | No | 0.00 | 0.00 | 0.27 | 0.27 |
| ABL2 | P42684 | 7 | T2DM | No | 0.15 | 0.00 | 0.08 | 0.18 |
| ALK | Q9UM73 | 7 | T1DM, Other | No | 0.15 | 0.00 | 0.06 | 0.17 |
| MDM2 | Q00987 | 6 | T1DM, T2DM | No | 0.00 | 0.00 | 0.25 | 0.25 |
| NUP58 | Q9BVL2 | 6 | T1DM | No | 0.21 | 0.00 | 0.00 | 0.21 |
| NLGN3 | Q9NZ94 | 6 | T2DM | No | 0.19 | 0.00 | 0.00 | 0.19 |
| FES | P07332 | 6 | – | No | 0.15 | 0.00 | 0.05 | 0.16 |
| PKDCC | Q504Y2 | 6 | – | No | 0.15 | 0.00 | 0.05 | 0.16 |
| RET | P07949 | 5 | T1DM, T2DM, Other | No | 0.80 | 0.00 | 0.27 | 0.85 |
| ENG | P17813 | 5 | T1DM, T2DM, Other | No | 0.27 | 0.00 | 0.00 | 0.27 |
| CIT | O14578 | 5 | – | No | 0.15 | 0.00 | 0.08 | 0.18 |
| ROS1 | P08922 | 5 | T2DM | No | 0.15 | 0.00 | 0.05 | 0.16 |
| GFRAL | Q6UXV0 | 4 | T2DM | No | 0.80 | 0.00 | 0.90 | 0.98 |
| MDFI | Q99750 | 4 | – | No | 0.28 | 0.00 | 0.00 | 0.28 |
| CBX3 | Q13185 | 4 | – | No | 0.27 | 0.00 | 0.00 | 0.27 |
| INSRR | P14616 | 4 | T2DM | No | 0.15 | 0.00 | 0.08 | 0.18 |
| MST1R | Q04912 | 3 | T2DM | No | 0.15 | 0.00 | 0.05 | 0.16 |
| FER | P16591 | 3 | Other | No | 0.15 | 0.00 | 0.05 | 0.16 |
| CCN2 | P29279 | - | T1DM, T2DM, Other | No | 0.00 | 0.00 | 0.28 | 0.28 |
| LIMK1 | P53667 | - | – | No | 0.15 | 0.00 | 0.05 | 0.16 |
| **GGT1** | | | | | | | | |
| DPP4 | P27487 | 38 | T1DM, T2DM, GDM, Other | Abbasi et al, 2016 | 0.00 | 0.00 | 0.23 | 0.23 |
| NAMPT | P43490 | 35 | T1DM, T2DM, GDM, Other | Network expansion | 0.19 | 0.00 | 0.00 | 0.19 |
| APOB | P04114 | 34 | T1DM, T2DM, GDM, Other | Abbasi et al, 2016 | 0.00 | 0.00 | 0.27 | 0.27 |
| GPX1 | P07203 | 22 | T1DM, T2DM, Other | No | 0.00 | 0.65 | 0.00 | 0.65 |
| GPX8 | Q8TED1 | 19 | – | No | 0.00 | 0.65 | 0.00 | 0.65 |
| GPX3 | P22352 | 19 | T1DM, T2DM, GDM, Other | No | 0.00 | 0.65 | 0.00 | 0.65 |
| GSTO2 | Q9H4Y5 | 18 | – | No | 0.00 | 0.65 | 0.00 | 0.65 |
| GSTA4 | O15217 | 17 | T2DM | No | 0.00 | 0.65 | 0.06 | 0.66 |
| HNF4A | P41235 | 17 | T1DM, T2DM, GDM, Other | No | 0.00 | 0.00 | 0.25 | 0.25 |
| GGT2 | P36268 | 16 | – | No | 0.54 | 0.00 | 0.00 | 0.54 |
| CYP2E1 | P05181 | 16 | T1DM, T2DM | No | 0.00 | 0.00 | 0.26 | 0.26 |
| GSTM5 | P46439 | 15 | T2DM | No | 0.00 | 0.65 | 0.14 | 0.69 |
| GPX2 | P18283 | 15 | T2DM | No | 0.00 | 0.65 | 0.00 | 0.65 |
| GSTM4 | Q03013 | 14 | – | No | 0.00 | 0.65 | 0.16 | 0.69 |
| GSTM1 | P09488 | 14 | T1DM, T2DM, GDM, Other | No | 0.00 | 0.65 | 0.14 | 0.69 |
| GPX7 | Q96SL4 | 14 | T2DM | No | 0.00 | 0.65 | 0.00 | 0.65 |
| GPX6 | P59796 | 14 | – | No | 0.00 | 0.65 | 0.00 | 0.65 |
| GSTT2B | P0CG30 | 14 | – | No | 0.00 | 0.65 | 0.00 | 0.65 |
| GSTA1 | P08263 | 13 | T1DM, T2DM, Other | No | 0.00 | 0.65 | 0.06 | 0.66 |
| GGT7 | Q9UJ14 | 13 | – | No | 0.00 | 0.65 | 0.00 | 0.65 |
| GPX5 | O75715 | 13 | – | No | 0.00 | 0.65 | 0.00 | 0.65 |
| SMARCAD1 | Q9H4L7 | 13 | T2DM | No | 0.16 | 0.00 | 0.00 | 0.16 |
| GSTM2 | P28161 | 12 | T2DM | No | 0.00 | 0.65 | 0.16 | 0.69 |
| GSTA2 | P09210 | 12 | – | No | 0.00 | 0.65 | 0.06 | 0.66 |
| GSTA3 | Q16772 | 12 | T2DM | No | 0.00 | 0.65 | 0.06 | 0.66 |
| MGST3 | O14880 | 12 | T2DM | No | 0.00 | 0.65 | 0.00 | 0.65 |
| GSTM3 | P21266 | 11 | – | No | 0.00 | 0.65 | 0.12 | 0.68 |
| GSTA5 | Q7RTV2 | 11 | – | No | 0.00 | 0.65 | 0.06 | 0.66 |
| MGST1 | P10620 | 11 | – | No | 0.00 | 0.65 | 0.00 | 0.65 |
| GYG1 | P46976 | 11 | T2DM | No | 0.27 | 0.00 | 0.00 | 0.27 |
| CD53 | P19397 | 11 | – | No | 0.27 | 0.00 | 0.00 | 0.27 |
| CANX | P27824 | 11 | – | No | 0.27 | 0.00 | 0.00 | 0.27 |
| GGT6 | Q6P531 | 10 | – | No | 0.00 | 0.65 | 0.00 | 0.65 |
| MGST2 | Q99735 | 10 | T2DM | No | 0.00 | 0.65 | 0.00 | 0.65 |
| HPGDS | O60760 | 10 | T1DM, T2DM | No | 0.00 | 0.65 | 0.05 | 0.65 |
| DNPEP | Q9ULA0 | 10 | – | No | 0.00 | 0.00 | 0.16 | 0.16 |
| GSTP1 | P09211 | 9 | T2DM, GDM, Other | No | 0.00 | 0.65 | 0.06 | 0.66 |
| CD81 | P60033 | 9 | – | No | 0.29 | 0.00 | 0.00 | 0.29 |
| GSTO1 | P78417 | 8 | T2DM | No | 0.00 | 0.65 | 0.00 | 0.65 |
| NUBPL | Q8TB37 | 8 | – | No | 0.17 | 0.00 | 0.00 | 0.17 |
| CD82 | P27701 | 7 | – | No | 0.27 | 0.00 | 0.00 | 0.27 |
| GGT5 | P36269 | 5 | T2DM | No | 0.00 | 0.65 | 0.00 | 0.65 |
| **GPT** | | | | | | | | |
| PRKAA2 | P54646 | 33 | T1DM, T2DM, GDM, Other | No | 0.22 | 0.00 | 0.00 | 0.22 |
| PRKAG1 | P54619 | 30 | Other | No | 0.24 | 0.00 | 0.00 | 0.24 |
| AGXT2 | Q9BYV1 | 21 | T2DM | No | 0.34 | 0.00 | 0.00 | 0.34 |
| H6PD | O95479 | 21 | T1DM, T2DM, Other | No | 0.21 | 0.00 | 0.00 | 0.21 |
| ALDH6A1 | Q02252 | 20 | T1DM | No | 0.33 | 0.00 | 0.00 | 0.33 |
| PRKAG2 | Q9UGJ0 | 20 | T2DM, Other | No | 0.24 | 0.00 | 0.00 | 0.24 |
| BDH2 | Q9BUT1 | 20 | T2DM | No | 0.16 | 0.00 | 0.00 | 0.16 |
| NPEPL1 | Q8NDH3 | 19 | – | No | 0.26 | 0.00 | 0.00 | 0.26 |
| ALDH9A1 | P49189 | 19 | – | No | 0.20 | 0.00 | 0.00 | 0.20 |
| ALDH16A1 | Q8IZ83 | 18 | – | No | 0.20 | 0.00 | 0.00 | 0.20 |
| DHRS1 | Q96LJ7 | 18 | – | No | 0.16 | 0.00 | 0.00 | 0.16 |
| HSD17B14 | Q9BPX1 | 17 | – | No | 0.16 | 0.00 | 0.00 | 0.16 |
| TKTL2 | Q9H0I9 | 15 | – | No | 0.28 | 0.00 | 0.00 | 0.28 |
| TKT | P29401 | 15 | T1DM, T2DM, GDM, Other | No | 0.28 | 0.00 | 0.00 | 0.28 |
| CAT | P04040 | 15 | T1DM, T2DM, GDM, Other | No | 0.27 | 0.00 | 0.00 | 0.27 |
| ALDH2 | P05091 | 15 | T1DM, T2DM | No | 0.26 | 0.00 | 0.00 | 0.26 |
| SASS6 | Q6UVJ0 | 15 | – | No | 0.15 | 0.00 | 0.00 | 0.15 |
| CES3 | Q6UWW8 | 15 | T2DM | No | 0.15 | 0.00 | 0.00 | 0.15 |
| ALDH8A1 | Q9H2A2 | 14 | – | No | 0.20 | 0.00 | 0.00 | 0.20 |
| BCAT2 | O15382 | 14 | T1DM, T2DM, Other | No | 0.00 | 0.00 | 0.20 | 0.20 |
| DECR1 | Q16698 | 14 | – | No | 0.16 | 0.00 | 0.00 | 0.16 |
| CES2 | O00748 | 13 | Other | No | 0.23 | 0.00 | 0.00 | 0.23 |
| HSPD1 | P10809 | 13 | T1DM, T2DM, Other | No | 0.17 | 0.00 | 0.00 | 0.17 |
| DECR2 | Q9NUI1 | 13 | – | No | 0.16 | 0.00 | 0.00 | 0.16 |
| CEL | P19835 | 13 | T1DM, T2DM, Other | No | 0.15 | 0.00 | 0.00 | 0.15 |
| ACO1 | P21399 | 13 | – | No | 0.15 | 0.00 | 0.00 | 0.15 |
| ACO2 | Q99798 | 12 | T1DM, T2DM | No | 0.34 | 0.00 | 0.00 | 0.34 |
| PEPD | P12955 | 12 | T2DM, GDM | No | 0.34 | 0.00 | 0.00 | 0.34 |
| FH | P07954 | 12 | T1DM, T2DM | No | 0.34 | 0.00 | 0.00 | 0.34 |
| LAP3 | P28838 | 12 | T1DM, T2DM | No | 0.26 | 0.00 | 0.00 | 0.26 |
| ALDH1A2 | O94788 | 12 | T2DM | No | 0.26 | 0.00 | 0.00 | 0.26 |
| TKTL1 | P51854 | 12 | – | No | 0.26 | 0.00 | 0.00 | 0.26 |
| CES1 | P23141 | 12 | T1DM, T2DM, Other | No | 0.23 | 0.00 | 0.00 | 0.23 |
| BCAT1 | P54687 | 12 | T1DM, T2DM | No | 0.00 | 0.00 | 0.20 | 0.20 |
| HSDL2 | Q6YN16 | 12 | T1DM | No | 0.16 | 0.00 | 0.00 | 0.16 |
| ALDH7A1 | P49419 | 11 | T1DM, T2DM | No | 0.34 | 0.00 | 0.00 | 0.34 |
| PHYHD1 | Q5SRE7 | 11 | – | Network expansion | 0.26 | 0.00 | 0.00 | 0.26 |
| SI | P14410 | 11 | T2DM | No | 0.25 | 0.00 | 0.00 | 0.25 |
| DHRS4 | Q9BTZ2 | 11 | – | No | 0.16 | 0.00 | 0.00 | 0.16 |
| CTBP2 | P56545 | 11 | T1DM, GDM | No | 0.15 | 0.00 | 0.00 | 0.15 |
| IREB2 | P48200 | 11 | Other | No | 0.15 | 0.00 | 0.00 | 0.15 |
| TALDO1 | P37837 | 10 | – | No | 0.34 | 0.00 | 0.00 | 0.34 |
| ALDH1B1 | P30837 | 10 | Other | No | 0.26 | 0.00 | 0.00 | 0.26 |
| GAA | P10253 | 10 | T1DM, T2DM, Other | No | 0.25 | 0.00 | 0.00 | 0.25 |
| G6PD | P11413 | 10 | T1DM | No | 0.24 | 0.00 | 0.00 | 0.24 |
| ALDH1A3 | P47895 | 10 | T2DM | No | 0.20 | 0.00 | 0.00 | 0.20 |
| TPI1 | P60174 | 9 | T1DM, T2DM, Other | No | 0.34 | 0.00 | 0.00 | 0.34 |
| GBA | P04062 | 9 | – | No | 0.19 | 0.00 | 0.00 | 0.19 |
| ATP6V1D | Q9Y5K8 | 9 | – | No | 0.18 | 0.00 | 0.00 | 0.18 |
| CES4A | Q5XG92 | 9 | – | No | 0.15 | 0.00 | 0.00 | 0.15 |
| ALDH1A1 | P00352 | 8 | T2DM | No | 0.26 | 0.00 | 0.00 | 0.26 |
| DHRS2 | Q13268 | 8 | T2DM | No | 0.16 | 0.00 | 0.00 | 0.16 |
| CTBP1 | Q13363 | 8 | T2DM | No | 0.15 | 0.00 | 0.00 | 0.15 |
| FOLR1 | P15328 | 7 | – | No | 0.00 | 0.00 | 0.47 | 0.47 |
| PHYH | O14832 | 7 | T2DM | No | 0.34 | 0.00 | 0.00 | 0.34 |
| ABAT | P80404 | 7 | T1DM | No | 0.34 | 0.00 | 0.00 | 0.34 |
| PHGDH | O43175 | 7 | T1DM, T2DM | No | 0.34 | 0.00 | 0.00 | 0.34 |
| CAPN1 | P07384 | 7 | T2DM | No | 0.27 | 0.00 | 0.00 | 0.27 |
| MGAM | O43451 | 7 | T1DM, T2DM, GDM | No | 0.25 | 0.00 | 0.00 | 0.25 |
| MYORG | Q6NSJ0 | 7 | – | No | 0.24 | 0.00 | 0.00 | 0.24 |
| DHRS4L2 | Q6PKH6 | 7 | – | No | 0.16 | 0.00 | 0.00 | 0.16 |
| AP2M1 | Q96CW1 | 7 | – | No | 0.16 | 0.00 | 0.00 | 0.16 |
| NLGN2 | Q8NFZ4 | 7 | – | No | 0.15 | 0.00 | 0.00 | 0.15 |
| NLGN1 | Q8N2Q7 | 7 | T2DM | No | 0.15 | 0.00 | 0.00 | 0.15 |
| NLGN4X | Q8N0W4 | 7 | – | No | 0.15 | 0.00 | 0.00 | 0.15 |
| CFL1 | P23528 | 6 | T1DM, T2DM | No | 0.27 | 0.00 | 0.00 | 0.27 |
| MDM2 | Q00987 | 6 | T1DM, T2DM | No | 0.00 | 0.00 | 0.24 | 0.24 |
| HLTF | Q14527 | 6 | T1DM | No | 0.16 | 0.00 | 0.00 | 0.16 |
| NLGN3 | Q9NZ94 | 6 | T2DM | No | 0.15 | 0.00 | 0.00 | 0.15 |
| DCAF7 | P61962 | 5 | T2DM | No | 0.17 | 0.00 | 0.00 | 0.17 |
| CES5A | Q6NT32 | 4 | – | No | 0.23 | 0.00 | 0.00 | 0.23 |
| RALA | P11233 | 4 | – | No | 0.21 | 0.00 | 0.00 | 0.21 |
| PECR | Q9BY49 | 3 | – | No | 0.16 | 0.00 | 0.00 | 0.16 |
| MGAM2 | Q2M2H8 | - | T2DM | No | 0.25 | 0.00 | 0.00 | 0.25 |
| PRKAB2 | O43741 | - | T2DM, Other | No | 0.22 | 0.00 | 0.00 | 0.22 |
| **IL18R1** | | | | | | | | |
| SCT | P09683 | 21 | T1DM, T2DM | No | 0.00 | 0.00 | 0.28 | 0.28 |
| IL18 | Q14116 | 19 | T1DM, T2DM, GDM, Other | Abbasi et al, 2016 | 0.93 | 0.90 | 0.91 | 1.00 |
| IL33 | O95760 | 15 | T1DM, T2DM, GDM, Other | No | 0.00 | 0.00 | 0.54 | 0.54 |
| IL37 | Q9NZH6 | 13 | T1DM, T2DM, GDM | No | 0.28 | 0.60 | 0.78 | 0.93 |
| IL18RAP | O95256 | 12 | T1DM | No | 0.80 | 0.90 | 0.91 | 1.00 |
| SIGIRR | Q6IA17 | 12 | – | No | 0.00 | 0.60 | 0.60 | 0.84 |
| IRAK1 | P51617 | 12 | T1DM, T2DM | No | 0.05 | 0.00 | 0.19 | 0.19 |
| SLC12A3 | P55017 | 12 | T1DM, T2DM | No | 0.18 | 0.00 | 0.00 | 0.18 |
| TICAM2 | Q86XR7 | 11 | – | No | 0.00 | 0.00 | 0.21 | 0.21 |
| DNMT3A | Q9Y6K1 | 10 | T1DM, T2DM, GDM | No | 0.00 | 0.00 | 0.43 | 0.43 |
| IL1RAPL1 | Q9NZN1 | 8 | – | No | 0.00 | 0.00 | 0.56 | 0.56 |
| **RTN4R** | | | | | | | | |
| BUD13 | Q9BRD0 | 31 | – | No | 0.17 | 0.00 | 0.00 | 0.17 |
| CAB39 | Q9Y376 | 23 | – | No | 0.17 | 0.00 | 0.11 | 0.23 |
| CISD3 | P0C7P0 | 23 | – | No | 0.17 | 0.00 | 0.00 | 0.17 |
| SORT1 | Q99523 | 20 | T2DM, Other | No | 0.00 | 0.00 | 0.38 | 0.38 |
| MRPS16 | Q9Y3D3 | 20 | – | No | 0.17 | 0.00 | 0.00 | 0.17 |
| SLC25A10 | Q9UBX3 | 20 | – | No | 0.16 | 0.00 | 0.00 | 0.16 |
| RPL3L | Q92901 | 20 | – | No | 0.17 | 0.00 | 0.00 | 0.16 |
| SNAPC4 | Q5SXM2 | 19 | T1DM, T2DM | No | 0.16 | 0.00 | 0.00 | 0.16 |
| PTPRQ | Q9UMZ3 | 18 | – | No | 0.27 | 0.00 | 0.00 | 0.27 |
| MRPL33 | O75394 | 18 | T1DM | No | 0.17 | 0.00 | 0.00 | 0.17 |
| CTH | P32929 | 17 | T1DM, T2DM | No | 0.00 | 0.00 | 0.25 | 0.25 |
| TNFRSF1B | P20333 | 17 | T1DM, T2DM, GDM | Abbasi et al, 2016 | 0.00 | 0.00 | 0.21 | 0.21 |
| PTPRA | P18433 | 17 | – | No | 0.18 | 0.00 | 0.00 | 0.18 |
| STK25 | O00506 | 17 | T1DM, T2DM | No | 0.17 | 0.00 | 0.00 | 0.17 |
| CAB39L | Q9H9S4 | 16 | – | No | 0.17 | 0.00 | 0.11 | 0.23 |
| PTPRE | P23469 | 16 | – | No | 0.18 | 0.00 | 0.00 | 0.18 |
| NWD1 | Q149M9 | 16 | – | No | 0.18 | 0.00 | 0.00 | 0.18 |
| NRXN3 | Q9Y4C0 | 16 | T2DM | No | 0.18 | 0.00 | 0.00 | 0.18 |
| FXN | Q16595 | 16 | T2DM | No | 0.17 | 0.00 | 0.00 | 0.17 |
| MRPS5 | P82675 | 16 | – | No | 0.17 | 0.00 | 0.00 | 0.17 |
| MRPL12 | P52815 | 16 | – | No | 0.16 | 0.00 | 0.00 | 0.16 |
| SNRPN | P63162 | 16 | – | No | 0.15 | 0.00 | 0.00 | 0.15 |
| SUOX | P51687 | 15 | T1DM, T2DM | No | 0.19 | 0.00 | 0.00 | 0.19 |
| PTPRT | O14522 | 15 | – | No | 0.18 | 0.00 | 0.00 | 0.18 |
| USHBP1 | Q8N6Y0 | 15 | – | No | 0.17 | 0.00 | 0.00 | 0.17 |
| MRPL21 | Q7Z2W9 | 15 | – | No | 0.17 | 0.00 | 0.00 | 0.17 |
| MRPL4 | Q9BYD3 | 15 | – | No | 0.16 | 0.00 | 0.00 | 0.16 |
| MT-ND1 | P03886 | 14 | T1DM, T2DM, GDM, Other | No | 0.00 | 0.00 | 0.61 | 0.61 |
| LTA | P01374 | 14 | T1DM, T2DM, GDM, Other | No | 0.00 | 0.00 | 0.44 | 0.44 |
| ZMAT5 | Q9UDW3 | 14 | T2DM | No | 0.17 | 0.00 | 0.00 | 0.17 |
| S1PR2 | O95136 | 13 | T2DM | No | 0.00 | 0.00 | 0.37 | 0.37 |
| KDR | P35968 | 13 | T1DM, T2DM, GDM | No | 0.08 | 0.00 | 0.26 | 0.29 |
| LY86 | O95711 | 13 | Other | No | 0.20 | 0.00 | 0.00 | 0.20 |
| PTPRD | P23468 | 13 | T2DM, GDM | No | 0.18 | 0.00 | 0.00 | 0.18 |
| SNRPB2 | P08579 | 13 | – | No | 0.18 | 0.00 | 0.00 | 0.18 |
| SART1 | O43290 | 13 | – | No | 0.17 | 0.00 | 0.00 | 0.17 |
| PPP1CC | P36873 | 13 | – | No | 0.16 | 0.00 | 0.05 | 0.16 |
| PPP1CB | P62140 | 13 | T1DM, T2DM | No | 0.16 | 0.00 | 0.05 | 0.16 |
| PPP3CB | P16298 | 13 | – | No | 0.16 | 0.00 | 0.05 | 0.16 |
| RNPC3 | Q96LT9 | 12 | T2DM | No | 0.18 | 0.00 | 0.00 | 0.18 |
| PTPRS | Q13332 | 12 | T1DM | No | 0.18 | 0.00 | 0.00 | 0.18 |
| STK24 | Q9Y6E0 | 12 | – | No | 0.17 | 0.00 | 0.00 | 0.17 |
| LSM8 | O95777 | 12 | – | No | 0.17 | 0.00 | 0.00 | 0.17 |
| PPP3CC | P48454 | 12 | – | No | 0.16 | 0.00 | 0.05 | 0.16 |
| PPP2CA | P67775 | 12 | T2DM | No | 0.16 | 0.00 | 0.05 | 0.16 |
| SNRPD1 | P62314 | 12 | – | No | 0.15 | 0.00 | 0.00 | 0.15 |
| MSRB3 | Q8IXL7 | 12 | – | No | 0.15 | 0.00 | 0.00 | 0.15 |
| MSRB2 | Q9Y3D2 | 12 | – | No | 0.15 | 0.00 | 0.00 | 0.15 |
| ACTB | P60709 | 11 | T1DM, T2DM, GDM, Other | No | 0.10 | 0.00 | 0.31 | 0.35 |
| ITGB1 | P05556 | 11 | T1DM, T2DM, GDM | No | 0.04 | 0.00 | 0.23 | 0.24 |
| BUD31 | P41223 | 11 | – | No | 0.21 | 0.00 | 0.00 | 0.21 |
| SNRPG | P62308 | 11 | – | No | 0.21 | 0.00 | 0.00 | 0.21 |
| PRPF39 | Q86UA1 | 11 | T1DM | No | 0.17 | 0.00 | 0.06 | 0.19 |
| PTPRK | Q15262 | 11 | T1DM | No | 0.18 | 0.00 | 0.00 | 0.18 |
| GFM1 | Q96RP9 | 11 | – | No | 0.17 | 0.00 | 0.00 | 0.17 |
| MRPL27 | Q9P0M9 | 11 | – | No | 0.17 | 0.00 | 0.00 | 0.17 |
| CWF19L2 | Q2TBE0 | 11 | – | No | 0.17 | 0.00 | 0.00 | 0.17 |
| MYBL1 | P10243 | 11 | – | No | 0.16 | 0.00 | 0.00 | 0.16 |
| POTEF | A5A3E0 | 11 | – | No | 0.10 | 0.00 | 0.10 | 0.15 |
| IFNG | P01579 | 10 | T1DM, T2DM, GDM, Other | No | 0.00 | 0.00 | 0.35 | 0.34 |
| PTPRC | P08575 | 10 | T1DM, T2DM, GDM | No | 0.18 | 0.00 | 0.09 | 0.22 |
| ITGAV | P06756 | 10 | T1DM, GDM | No | 0.00 | 0.00 | 0.20 | 0.20 |
| MRPL17 | Q9NRX2 | 10 | – | No | 0.17 | 0.00 | 0.00 | 0.17 |
| NTN5 | Q8WTR8 | 10 | T1DM | No | 0.17 | 0.00 | 0.00 | 0.17 |
| CTNNB1 | P35222 | 10 | T1DM, T2DM, GDM | No | 0.06 | 0.00 | 0.14 | 0.16 |
| PPEF2 | O14830 | 10 | – | No | 0.16 | 0.00 | 0.05 | 0.16 |
| ANPEP | P15144 | 9 | T1DM, T2DM, GDM, Other | No | 0.00 | 0.00 | 0.90 | 0.90 |
| APP | P05067 | 9 | T1DM, T2DM, GDM, Other | No | 0.05 | 0.00 | 0.84 | 0.84 |
| ROBO2 | Q9HCK4 | 9 | T2DM | No | 0.13 | 0.00 | 0.19 | 0.27 |
| IL20RA | Q9UHF4 | 9 | T2DM | No | 0.00 | 0.00 | 0.25 | 0.25 |
| MYOC | Q99972 | 9 | – | No | 0.06 | 0.00 | 0.23 | 0.24 |
| CNTNAP1 | P78357 | 9 | T2DM | No | 0.00 | 0.00 | 0.19 | 0.19 |
| SNRPA | P09012 | 9 | – | No | 0.18 | 0.00 | 0.00 | 0.18 |
| EFL1 | Q7Z2Z2 | 9 | – | No | 0.17 | 0.00 | 0.00 | 0.17 |
| EEF2 | P13639 | 9 | – | No | 0.17 | 0.00 | 0.00 | 0.17 |
| PPP5C | P53041 | 9 | – | No | 0.16 | 0.00 | 0.05 | 0.16 |
| NAA38 | Q9BRA0 | 9 | – | No | 0.15 | 0.00 | 0.00 | 0.15 |
| POTEI | P0CG38 | 9 | – | No | 0.10 | 0.00 | 0.10 | 0.15 |
| LSM6 | P62312 | 9 | – | No | 0.15 | 0.00 | 0.00 | 0.15 |
| RHO | P08100 | 9 | T1DM, T2DM | No | 0.00 | 0.00 | 0.15 | 0.15 |
| NGFR | P08138 | 8 | T1DM, T2DM | No | 0.50 | 0.90 | 0.90 | 0.99 |
| MAG | P20916 | 8 | – | No | 0.24 | 0.90 | 0.73 | 0.98 |
| LILRB3 | O75022 | 8 | – | No | 0.00 | 0.00 | 0.42 | 0.42 |
| RTN1 | Q16799 | 8 | – | No | 0.17 | 0.00 | 0.31 | 0.41 |
| ADAM22 | Q9P0K1 | 8 | T1DM | No | 0.00 | 0.00 | 0.36 | 0.36 |
| PLRG1 | O43660 | 8 | – | No | 0.21 | 0.00 | 0.00 | 0.21 |
| SF3B3 | Q15393 | 8 | – | No | 0.21 | 0.00 | 0.00 | 0.21 |
| OLFM1 | Q99784 | 8 | – | No | 0.06 | 0.00 | 0.19 | 0.20 |
| NTN1 | O95631 | 8 | T1DM, T2DM, GDM | No | 0.17 | 0.00 | 0.07 | 0.19 |
| FGFR1 | P11362 | 8 | T1DM, T2DM | No | 0.18 | 0.00 | 0.00 | 0.17 |
| LSM7 | Q9UK45 | 8 | – | No | 0.17 | 0.00 | 0.00 | 0.17 |
| STK26 | Q9P289 | 8 | Other | No | 0.17 | 0.00 | 0.00 | 0.17 |
| SRRM2 | Q9UQ35 | 8 | T2DM | No | 0.17 | 0.00 | 0.00 | 0.17 |
| PPP3CA | Q08209 | 8 | Other | No | 0.16 | 0.00 | 0.05 | 0.16 |
| TTF1 | Q15361 | 8 | – | No | 0.16 | 0.00 | 0.00 | 0.16 |
| PPP4C | P60510 | 8 | T1DM | No | 0.16 | 0.00 | 0.05 | 0.16 |
| MYB | P10242 | 8 | T2DM, Other | No | 0.16 | 0.00 | 0.00 | 0.16 |
| PPP2CB | P62714 | 8 | – | No | 0.16 | 0.00 | 0.05 | 0.16 |
| SNRPD2 | P62316 | 8 | T2DM | No | 0.15 | 0.00 | 0.00 | 0.15 |
| SNRPB | P14678 | 8 | – | No | 0.15 | 0.00 | 0.00 | 0.15 |
| SNIP1 | Q8TAD8 | 8 | – | No | 0.15 | 0.00 | 0.00 | 0.15 |
| ARHGDIA | P52565 | 7 | T1DM, Other | No | 0.00 | 0.90 | 0.00 | 0.90 |
| RBM25 | P49756 | 7 | – | No | 0.35 | 0.00 | 0.00 | 0.35 |
| SNRPF | P62306 | 7 | – | No | 0.22 | 0.00 | 0.00 | 0.22 |
| SNW1 | Q13573 | 7 | – | No | 0.21 | 0.00 | 0.00 | 0.21 |
| XAB2 | Q9HCS7 | 7 | GDM | No | 0.18 | 0.00 | 0.06 | 0.20 |
| ROBO1 | Q9Y6N7 | 7 | – | No | 0.13 | 0.00 | 0.11 | 0.19 |
| SF3B5 | Q9BWJ5 | 7 | – | No | 0.18 | 0.00 | 0.00 | 0.18 |
| ASCC3 | Q8N3C0 | 7 | – | No | 0.17 | 0.00 | 0.00 | 0.17 |
| MAP3K8 | P41279 | 7 | T2DM | No | 0.17 | 0.00 | 0.00 | 0.17 |
| CWC22 | Q9HCG8 | 7 | T1DM | No | 0.17 | 0.00 | 0.00 | 0.17 |
| CWC25 | Q9NXE8 | 7 | – | No | 0.17 | 0.00 | 0.00 | 0.17 |
| MRPL20 | Q9BYC9 | 7 | – | No | 0.17 | 0.00 | 0.00 | 0.17 |
| PPP1CA | P62136 | 7 | – | No | 0.16 | 0.00 | 0.05 | 0.16 |
| LSM3 | P62310 | 7 | – | No | 0.16 | 0.00 | 0.00 | 0.16 |
| PPEF1 | O14829 | 7 | – | No | 0.16 | 0.00 | 0.05 | 0.16 |
| CNTN1 | Q12860 | 7 | T1DM | No | 0.08 | 0.00 | 0.12 | 0.15 |
| LSM5 | Q9Y4Y9 | 7 | – | No | 0.15 | 0.00 | 0.00 | 0.15 |
| EGFR | P00533 | 7 | T1DM, T2DM, GDM, Other | No | 0.07 | 0.00 | 0.12 | 0.15 |
| SNRPD3 | P62318 | 7 | – | No | 0.15 | 0.00 | 0.00 | 0.15 |
| MSRB1 | Q9NZV6 | 7 | – | No | 0.15 | 0.00 | 0.00 | 0.15 |
| RTN4 | Q9NQC3 | 6 | T2DM | No | 0.24 | 0.90 | 0.90 | 0.99 |
| KIAA0319L | Q8IZA0 | 6 | T1DM | No | 0.27 | 0.00 | 0.47 | 0.59 |
| TNFSF13B | Q9Y275 | 6 | T1DM, T2DM, GDM | No | 0.00 | 0.00 | 0.44 | 0.44 |
| PRPF19 | Q9UMS4 | 6 | – | No | 0.22 | 0.00 | 0.00 | 0.22 |
| SF3A3 | Q12874 | 6 | T1DM | No | 0.20 | 0.00 | 0.00 | 0.20 |
| HEATR6 | Q6AI08 | 6 | – | No | 0.18 | 0.00 | 0.06 | 0.20 |
| NTNG1 | Q9Y2I2 | 6 | – | No | 0.20 | 0.00 | 0.00 | 0.20 |
| TFIP11 | Q9UBB9 | 6 | – | No | 0.18 | 0.00 | 0.00 | 0.18 |
| PTPRU | Q92729 | 6 | – | No | 0.18 | 0.00 | 0.00 | 0.18 |
| NRXN2 | Q9P2S2 | 6 | – | No | 0.18 | 0.00 | 0.00 | 0.18 |
| NTNG2 | Q96CW9 | 6 | T2DM | No | 0.17 | 0.00 | 0.00 | 0.17 |
| RPS2 | P15880 | 6 | – | No | 0.17 | 0.00 | 0.00 | 0.17 |
| NTN3 | O00634 | 6 | – | No | 0.17 | 0.00 | 0.00 | 0.17 |
| EFTUD2 | Q15029 | 6 | – | No | 0.17 | 0.00 | 0.00 | 0.17 |
| MRPS9 | P82933 | 6 | T2DM | No | 0.17 | 0.00 | 0.00 | 0.17 |
| PRPF6 | O94906 | 6 | T1DM | No | 0.17 | 0.00 | 0.00 | 0.17 |
| RPL4 | P36578 | 6 | – | No | 0.16 | 0.00 | 0.00 | 0.16 |
| DMTF1 | Q9Y222 | 6 | – | No | 0.16 | 0.00 | 0.00 | 0.16 |
| SLU7 | O95391 | 6 | T1DM | No | 0.15 | 0.00 | 0.00 | 0.15 |
| LINGO1 | Q96FE5 | 5 | T2DM | No | 0.48 | 0.90 | 0.90 | 0.99 |
| OMG | P23515 | 5 | T2DM | No | 0.30 | 0.90 | 0.90 | 0.99 |
| LGI1 | O95970 | 5 | Other | No | 0.00 | 0.00 | 0.29 | 0.29 |
| RTN4RL1 | Q86UN2 | 5 | – | No | 0.00 | 0.00 | 0.29 | 0.29 |
| MYCN | P04198 | 5 | T2DM | No | 0.00 | 0.00 | 0.26 | 0.26 |
| PRPF40B | Q6NWY9 | 5 | – | No | 0.24 | 0.00 | 0.00 | 0.24 |
| SF3B2 | Q13435 | 5 | – | No | 0.21 | 0.00 | 0.06 | 0.23 |
| RBM22 | Q9NW64 | 5 | – | No | 0.21 | 0.00 | 0.00 | 0.21 |
| CCDC130 | P13994 | 5 | – | No | 0.18 | 0.00 | 0.00 | 0.18 |
| PTPRM | P28827 | 5 | T2DM | No | 0.18 | 0.00 | 0.00 | 0.18 |
| HFM1 | A2PYH4 | 5 | T1DM, T2DM | No | 0.17 | 0.00 | 0.00 | 0.17 |
| PPT1 | P50897 | 5 | – | No | 0.16 | 0.00 | 0.00 | 0.16 |
| RPL3 | P39023 | 5 | – | No | 0.17 | 0.00 | 0.00 | 0.16 |
| MYBL2 | P10244 | 5 | T2DM | No | 0.16 | 0.00 | 0.00 | 0.16 |
| MRPL3 | P09001 | 5 | T2DM | No | 0.17 | 0.00 | 0.00 | 0.16 |
| CDC5L | Q99459 | 5 | – | No | 0.16 | 0.00 | 0.00 | 0.16 |
| RPS16 | P62249 | 5 | – | No | 0.16 | 0.00 | 0.00 | 0.16 |
| SNRPE | P62304 | 5 | – | No | 0.15 | 0.00 | 0.00 | 0.15 |
| POTEE | Q6S8J3 | 5 | – | No | 0.10 | 0.00 | 0.10 | 0.15 |
| LSM10 | Q969L4 | 5 | – | No | 0.15 | 0.00 | 0.00 | 0.15 |
| PRPF8 | Q6P2Q9 | 4 | – | No | 0.21 | 0.00 | 0.00 | 0.21 |
| CDC40 | O60508 | 4 | – | No | 0.18 | 0.00 | 0.00 | 0.18 |
| PTPRF | P10586 | 4 | T2DM | No | 0.18 | 0.00 | 0.00 | 0.18 |
| SF3A1 | Q15459 | 4 | – | No | 0.18 | 0.00 | 0.00 | 0.18 |
| LSM4 | Q9Y4Z0 | 4 | – | No | 0.17 | 0.00 | 0.00 | 0.17 |
| AQR | O60306 | 4 | T2DM | No | 0.17 | 0.00 | 0.00 | 0.17 |
| SNRPC | P09234 | 4 | – | No | 0.17 | 0.00 | 0.00 | 0.17 |
| RBMX2 | Q9Y388 | 4 | – | No | 0.15 | 0.00 | 0.00 | 0.15 |
| TNFRSF19 | Q9NS68 | 3 | – | No | 0.00 | 0.00 | 0.88 | 0.88 |
| RHOH | Q15669 | 3 | – | No | 0.10 | 0.00 | 0.61 | 0.64 |
| RTN4RL2 | Q86UN3 | 3 | – | No | 0.00 | 0.00 | 0.27 | 0.27 |
| ROBO3 | Q96MS0 | 3 | – | No | 0.13 | 0.00 | 0.14 | 0.22 |
| CRNKL1 | Q9BZJ0 | 3 | – | No | 0.21 | 0.00 | 0.00 | 0.21 |
| SYF2 | O95926 | 3 | – | No | 0.21 | 0.00 | 0.00 | 0.21 |
| BCAS2 | O75934 | 3 | – | No | 0.18 | 0.00 | 0.00 | 0.18 |
| SF3A2 | Q15428 | 3 | – | No | 0.18 | 0.00 | 0.00 | 0.18 |
| SNRNP200 | O75643 | 3 | – | No | 0.17 | 0.00 | 0.00 | 0.17 |
| PPP1R42 | Q7Z4L9 | 3 | – | No | 0.16 | 0.00 | 0.00 | 0.16 |
| SNRNP40 | Q96DI7 | 3 | – | No | 0.16 | 0.00 | 0.00 | 0.16 |
| DAW1 | Q8N136 | 3 | – | No | 0.16 | 0.00 | 0.00 | 0.16 |
| SF3B1 | O75533 | 2 | – | No | 0.21 | 0.00 | 0.00 | 0.21 |
| WDR25 | Q64LD2 | - | – | No | 0.18 | 0.00 | 0.00 | 0.18 |
| GPATCH8 | Q9UKJ3 | - | – | No | 0.18 | 0.00 | 0.00 | 0.18 |
| ISY1 | Q9ULR0 | - | – | No | 0.18 | 0.00 | 0.00 | 0.18 |
| YJU2 | Q9BW85 | - | – | No | 0.18 | 0.00 | 0.00 | 0.18 |
| ZMAT2 | Q96NC0 | - | – | No | 0.17 | 0.00 | 0.00 | 0.17 |
| PRPF4 | O43172 | - | – | No | 0.16 | 0.00 | 0.00 | 0.16 |
| PPP5D1 | E7EU14 | - | – | No | 0.16 | 0.00 | 0.05 | 0.16 |
| POTEJ | P0CG39 | - | – | No | 0.10 | 0.00 | 0.10 | 0.15 |
| TNF | P01375 | - | T1DM, T2DM, GDM, Other | No | 0.00 | 0.00 | 0.88 | 0.88 |
| MOG | Q16653 | - | T1DM, Other | No | 0.00 | 0.00 | 0.34 | 0.34 |
| PPP1R11 | O60927 | - | T1DM | No | 0.18 | 0.00 | 0.08 | 0.22 |
| CWC15 | Q9P013 | - | – | No | 0.21 | 0.00 | 0.00 | 0.21 |
| SF3B4 | Q15427 | - | T2DM | No | 0.18 | 0.00 | 0.00 | 0.18 |
| NRXN1 | Q9ULB1 | - | T1DM | No | 0.18 | 0.00 | 0.00 | 0.18 |
| SRRM3 | A6NNA2 | - | – | No | 0.17 | 0.00 | 0.00 | 0.17 |
| PPT2 | Q9UMR5 | - | T2DM | No | 0.16 | 0.00 | 0.00 | 0.16 |
| PRPF3 | O43395 | - | T1DM | No | 0.16 | 0.00 | 0.00 | 0.16 |
| LSM2 | Q9Y333 | - | – | No | 0.15 | 0.00 | 0.00 | 0.15 |

#### High-confidence interactions only

Protein-protein interaction partners

|  | | Diabetes association | | | Confidence scores for interaction | | | |
| --- | --- | --- | --- | --- | --- | --- | --- | --- |
| Interaction partner | UniProt ID | Diabetes textmining % | Diabetes associations | Suggested diabetes biomarker | Experimental score | Database score | Textmining score | Combined score |
| **CRP** | | | | | | | | |
| OLR1 | P78380 | 26 | T2DM, GDM | RHAPSODY partner suggestion | 0.00 | 0.00 | 0.88 | 0.88 |
| C1QA | P02745 | 13 | – | No | 0.57 | 0.60 | 0.26 | 0.86 |
| FCN2 | Q15485 | 11 | T1DM | Network expansion | 0.27 | 0.00 | 0.83 | 0.87 |
| C4A | P0C0L4 | 11 | T1DM, T2DM, GDM, Other | No | 0.00 | 0.00 | 0.72 | 0.72 |
| CFH | P08603 | 10 | T1DM, T2DM, GDM | Network expansion | 0.73 | 0.00 | 0.90 | 0.97 |
| FCGR2A | P12318 | 10 | T1DM, T2DM, Other | No | 0.46 | 0.00 | 0.90 | 0.94 |
| C3 | P01024 | 10 | T1DM, T2DM, GDM, Other | Abbasi et al, 2016 | 0.48 | 0.00 | 0.66 | 0.82 |
| FCGR2B | P31994 | 10 | T2DM, Other | No | 0.00 | 0.00 | 0.75 | 0.75 |
| C4BPA | P04003 | 9 | – | No | 0.42 | 0.00 | 0.54 | 0.72 |
| FCN1 | O00602 | 8 | T1DM | No | 0.30 | 0.00 | 0.86 | 0.90 |
| CFHR4 | Q92496 | 6 | – | No | 0.57 | 0.00 | 0.76 | 0.89 |
| FCGR1A | P12314 | - | – | No | 0.00 | 0.00 | 0.94 | 0.94 |
| **FAS** | | | | | | | | |
| TNFRSF10A | O00220 | 22 | T1DM, T2DM, GDM | No | 0.00 | 0.60 | 0.42 | 0.76 |
| MAP3K5 | Q99683 | 17 | T1DM, T2DM | No | 0.64 | 0.90 | 0.00 | 0.96 |
| CAV1 | Q03135 | 16 | T1DM, T2DM, GDM, Other | No | 0.48 | 0.00 | 0.46 | 0.71 |
| FASLG | P48023 | 12 | T1DM, T2DM, Other | No | 0.65 | 0.90 | 0.90 | 1.00 |
| FAF1 | Q9UNN5 | 12 | T2DM | No | 0.50 | 0.90 | 0.45 | 0.97 |
| TNFRSF10B | O14763 | 11 | T1DM, T2DM | No | 0.29 | 0.60 | 0.00 | 0.70 |
| PLCG1 | P19174 | 11 | T2DM | No | 0.27 | 0.00 | 0.61 | 0.70 |
| RIPK1 | Q13546 | 10 | – | No | 0.44 | 0.90 | 0.47 | 0.97 |
| CASP10 | Q92851 | 10 | – | No | 0.52 | 0.90 | 0.47 | 0.97 |
| FAIM2 | Q9BWQ8 | 10 | T2DM, GDM | No | 0.27 | 0.90 | 0.00 | 0.92 |
| CFLAR | O15519 | 9 | T2DM | No | 0.49 | 0.90 | 0.45 | 0.97 |
| SRC | P12931 | 9 | T1DM, T2DM | No | 0.48 | 0.90 | 0.20 | 0.95 |
| BTK | Q06187 | 9 | – | No | 0.00 | 0.90 | 0.07 | 0.90 |
| SYK | P43405 | 9 | Other | No | 0.00 | 0.90 | 0.00 | 0.90 |
| SUMO1 | P63165 | 9 | T1DM, T2DM | No | 0.48 | 0.00 | 0.61 | 0.79 |
| CASP8 | Q14790 | 8 | T1DM, T2DM | No | 0.53 | 0.90 | 0.94 | 1.00 |
| FADD | Q13158 | 8 | T1DM | No | 0.95 | 0.90 | 0.91 | 1.00 |
| EGFR | P00533 | 7 | T1DM, T2DM, GDM, Other | No | 0.00 | 0.00 | 0.75 | 0.75 |
| DAXX | Q9UER7 | - | T1DM | No | 0.66 | 0.90 | 0.39 | 0.98 |
| TRADD | Q15628 | - | – | No | 0.46 | 0.90 | 0.28 | 0.96 |
| CALM1 | P0DP23 | - | T1DM, GDM | No | 0.80 | 0.00 | 0.00 | 0.80 |
| **GDF15** | | | | | | | | |
| RET | P07949 | 5 | T1DM, T2DM, Other | No | 0.80 | 0.00 | 0.27 | 0.85 |
| GFRAL | Q6UXV0 | 4 | T2DM | No | 0.80 | 0.00 | 0.90 | 0.98 |
| **IL18R1** | | | | | | | | |
| IL18 | Q14116 | 19 | T1DM, T2DM, GDM, Other | Abbasi et al, 2016 | 0.93 | 0.90 | 0.91 | 1.00 |
| IL37 | Q9NZH6 | 13 | T1DM, T2DM, GDM | No | 0.28 | 0.60 | 0.78 | 0.93 |
| IL18RAP | O95256 | 12 | T1DM | No | 0.80 | 0.90 | 0.91 | 1.00 |
| SIGIRR | Q6IA17 | 12 | – | No | 0.00 | 0.60 | 0.60 | 0.84 |
| **RTN4R** | | | | | | | | |
| ANPEP | P15144 | 9 | T1DM, T2DM, GDM, Other | No | 0.00 | 0.00 | 0.90 | 0.90 |
| APP | P05067 | 9 | T1DM, T2DM, GDM, Other | No | 0.05 | 0.00 | 0.84 | 0.84 |
| NGFR | P08138 | 8 | T1DM, T2DM | No | 0.50 | 0.90 | 0.90 | 0.99 |
| MAG | P20916 | 8 | – | No | 0.24 | 0.90 | 0.73 | 0.98 |
| ARHGDIA | P52565 | 7 | T1DM, Other | No | 0.00 | 0.90 | 0.00 | 0.90 |
| RTN4 | Q9NQC3 | 6 | T2DM | No | 0.24 | 0.90 | 0.90 | 0.99 |
| LINGO1 | Q96FE5 | 5 | T2DM | No | 0.48 | 0.90 | 0.90 | 0.99 |
| OMG | P23515 | 5 | T2DM | No | 0.30 | 0.90 | 0.90 | 0.99 |
| TNFRSF19 | Q9NS68 | 3 | – | No | 0.00 | 0.00 | 0.88 | 0.88 |
| TNF | P01375 | - | T1DM, T2DM, GDM, Other | No | 0.00 | 0.00 | 0.88 | 0.88 |

#### About

Summary table showing all interaction partners for each target
together with additional information:

- **UniProt ID (UniProt)**: Link to UniProt for
  additional information on the target
- **Diabetes textmining % (in-house data)**:
  Percentage of co-mentions from textmining relating to diabetes vs. all
  other diseases. For more information on the data source, see Westergaard et
  al. PLoS Computational Biology 2018)
- **Diabetes associations (Open Targets)**:
  Gene-disease (diabetes mellitus) association through experiments or
  clinical trials. Direct associations with T1DM, T2DM and GDM are shown
  specifically, while all other diabetes mellitus diseases are included in
  “Other”.
- **Suggested biomarker**: Known and suggested
  biomarkers from Abbasi
  et al, 2016, RHAPSODY partners (collected by the RHAPSODY biomarker
  taskforce) and based on PPI-network analysis of known biomarkers
  (in-house Analysis).
- **Experimental score**: STRING experimental score
  derived from experimental data, such as, affinity chromatography. The
  experimental data is extracted from the databases BIND, DIP, GRID, HPRD,
  IntAct, MINT, and PID.
- **Database score**: STRING database score derived
  from curated data of Biocarta, BioCyc, GO, KEGG, and Reactome.
- **Textmining score**: STRING textmining score
  derived from co-occurrence of gene/protein names in abstracts
- **Combined score**: STRING combined score of
  interaction confidence. Interactions with a combined score > 0.7 is
  deemed a high-confidence interaction.

High-confidence interaction partners with a confidence score ≥ 0.156
are shown in blue,
while suggested interaction partners are shown in gray.

en-dash (-) indicates that no information was available for the
target.

## 4.2 Over Representation Analysis

### 4.2.1 Network features

Over-represented terms were found for:

Biological process GO-terms, Cellular component GO-terms, Molecular
function GO-terms, KEGG pathways, Reactome pathways, Wikipathways,
GLAD4U diseases, GLAD4U drugs

#### Biological process GO-terms

Biological process GO-term enrichment in PPI networks

#### Cellular component GO-terms

Cellular component GO-term enrichment in PPI networks

#### Molecular function GO-terms

Molecular function GO-term enrichment in PPI networks

#### KEGG pathways

KEGG pathways enrichment in PPI networks

#### Reactome pathways

Reactome pathways enrichment in PPI networks

#### Wikipathways

Wikipathways enrichment in PPI networks

#### GLAD4U diseases

GLAD4U disease enrichment in PPI networks

#### GLAD4U drugs

GLAD4U drug enrichment in PPI networks

#### Table

Shared enrichment terms

| Term | Source link | Targets | Enrichment ratio | FDR |
| --- | --- | --- | --- | --- |
| **Biological process GO-terms** | | | | |
| membrane lipid metabolic process | link | ENPP7 | 63 | 0.00e+00 |
| peptide catabolic process | link | GGT1 | 56 | 5.37e-05 |
| cellular modified amino acid catabolic process | link | GGT1 | 53 | 5.85e-05 |
| interferon-gamma production | link | IL18R1 | 53 | 5.08e-04 |
| protein activation cascade | link | CRP | 47 | 0.00e+00 |
| cellular modified amino acid biosynthetic process | link | GGT1 | 39 | 1.38e-05 |
| natural killer cell activation | link | IL18R1 | 38 | 0.060 |
| cofactor catabolic process | link | GGT1 | 36 | 1.51e-06 |
| liposaccharide metabolic process | link | ENPP7 | 36 | 5.55e-05 |
| cellular modified amino acid metabolic process | link | GGT1 | 34 | 0.00e+00 |
| NIK/NF-kappaB signaling | link | IL18R1 | 34 | 0.007 |
| production of molecular mediator of immune response | link | IL18R1 | 29 | 0.001 |
| ammonium ion metabolic process | link | ENPP7 | 27 | 1.22e-06 |
| fatty acid derivative metabolic process | link | GGT1 | 25 | 7.09e-10 |
| necrotic cell death | link | FAS | 25 | 8.41e-10 |
| acute inflammatory response | link | CRP | 24 | 0.00e+00 |
| sulfur compound metabolic process | link | GGT1 | 24 | 0.00e+00 |
| humoral immune response | link | CRP | 23 | 0.00e+00 |
| interleukin-8 production | link | CRP | 23 | 1.77e-06 |
| response to xenobiotic stimulus | link | GGT1 | 20 | 6.29e-14 |
| adaptive thermogenesis | link | IL18R1 | 19 | 0.196 |
| lymphocyte mediated immunity | link | IL18R1 | 18 | 0.028 |
| regulation of DNA-binding transcription factor activity | link | IL18R1 | 17 | 0.001 |
| lymphocyte activation involved in immune response | link | IL18R1 | 17 | 0.215 |
| positive regulation of cytokine production | link | IL18R1 | 16 | 0.001 |
| phagocytosis | link | CRP | 15 | 1.18e-12 |
| extrinsic apoptotic signaling pathway | link | FAS | 15 | 0.00e+00 |
| adaptive immune response | link | IL18R1 | 15 | 0.007 |
| lymphocyte mediated immunity | link | CRP | 14 | 1.33e-10 |
| regulation of immune effector process | link | IL18R1 | 14 | 0.008 |
| foam cell differentiation | link | CRP | 12 | 0.218 |
| activation of protein kinase activity | link | GDF15 | 12 | 4.72e-14 |
| response to transforming growth factor beta | link | GDF15 | 11 | 2.11e-08 |
| adaptive immune response | link | CRP | 10 | 2.16e-10 |
| fatty acid metabolic process | link | GGT1 | 10 | 2.49e-06 |
| dicarboxylic acid metabolic process | link | GGT1 | 10 | 0.153 |
| regulation of apoptotic signaling pathway | link | FAS | 10 | 0.00e+00 |
| positive regulation of proteolysis | link | FAS | 10 | 0.00e+00 |
| lipid catabolic process | link | ENPP7 | 10 | 0.061 |
| response to BMP | link | GDF15 | 10 | 3.21e-04 |
| cytokine secretion | link | CRP | 9 | 1.60e-04 |
| organic acid biosynthetic process | link | GGT1 | 9 | 7.82e-06 |
| response to estradiol | link | GGT1 | 9 | 0.187 |
| organic acid biosynthetic process | link | GPT | 9 | 5.54e-08 |
| regulation of peptidase activity | link | FAS | 9 | 0.00e+00 |
| transmembrane receptor protein serine/threonine kinase signaling pathway | link | GDF15 | 9 | 5.61e-08 |
| cofactor biosynthetic process | link | GGT1 | 8 | 0.007 |
| small molecule catabolic process | link | GPT | 8 | 1.27e-07 |
| phospholipid metabolic process | link | ENPP7 | 8 | 0.040 |
| regulation of protein serine/threonine kinase activity | link | GDF15 | 8 | 8.41e-11 |
| protein kinase B signaling | link | GDF15 | 8 | 1.44e-05 |
| feeding behavior | link | GDF15 | 8 | 0.048 |
| response to mechanical stimulus | link | FAS | 7 | 3.11e-06 |
| response to peptide | link | GDF15 | 7 | 2.76e-09 |
| response to axon injury | link | RTN4R | 7 | 0.009 |
| response to molecule of bacterial origin | link | GGT1 | 6 | 0.112 |
| cellular response to external stimulus | link | FAS | 6 | 1.10e-07 |
| stress-activated protein kinase signaling cascade | link | FAS | 6 | 1.40e-06 |
| T cell activation | link | IL18R1 | 6 | 0.630 |
| multicellular organismal homeostasis | link | IL18R1 | 6 | 0.640 |
| leukocyte differentiation | link | IL18R1 | 6 | 0.678 |
| regulation of developmental growth | link | GDF15 | 6 | 8.03e-04 |
| maintenance of location | link | CRP | 5 | 0.018 |
| regulation of inflammatory response | link | GGT1 | 5 | 0.148 |
| cellular amino acid metabolic process | link | GPT | 5 | 0.003 |
| cellular response to environmental stimulus | link | FAS | 5 | 2.62e-05 |
| negative regulation of growth | link | GDF15 | 5 | 0.004 |
| negative regulation of cell projection organization | link | RTN4R | 5 | 0.001 |
| regeneration | link | RTN4R | 5 | 0.001 |
| regulation of peptide secretion | link | CRP | 4 | 0.028 |
| lipid localization | link | CRP | 4 | 0.032 |
| defense response to other organism | link | CRP | 4 | 0.057 |
| reactive oxygen species metabolic process | link | CRP | 4 | 0.354 |
| cellular amino acid metabolic process | link | GGT1 | 4 | 0.431 |
| response to nutrient levels | link | FAS | 4 | 1.40e-04 |
| response to oxygen levels | link | FAS | 4 | 0.001 |
| multicellular organism growth | link | GDF15 | 4 | 0.150 |
| cell-cell fusion | link | GDF15 | 4 | 0.510 |
| syncytium formation | link | GDF15 | 4 | 0.516 |
| axon development | link | RTN4R | 4 | 1.74e-08 |
| negative regulation of nervous system development | link | RTN4R | 4 | 3.16e-04 |
| negative regulation of cell development | link | RTN4R | 4 | 0.002 |
| response to nutrient levels | link | GDF15 | 3 | 0.063 |
| regulation of neuron projection development | link | RTN4R | 3 | 9.25e-05 |
| regulation of cell morphogenesis | link | RTN4R | 3 | 0.006 |
| developmental growth involved in morphogenesis | link | RTN4R | 3 | 0.023 |
| negative regulation of growth | link | RTN4R | 3 | 0.045 |
| forebrain development | link | RTN4R | 3 | 0.095 |
| muscle cell differentiation | link | GDF15 | 2 | 0.269 |
| cell growth | link | RTN4R | 2 | 0.039 |
| regulation of developmental growth | link | RTN4R | 2 | 0.295 |
| negative regulation of response to external stimulus | link | RTN4R | 2 | 0.474 |
| regulation of response to wounding | link | RTN4R | 2 | 0.522 |
| regulation of anatomical structure size | link | RTN4R | 1 | 0.956 |
| **Cellular component GO-terms** | | | | |
| membrane region | link | FAS | 8 | 1.15e-12 |
| dendritic shaft | link | RTN4R | 4 | 0.669 |
| membrane region | link | RTN4R | 3 | 0.002 |
| glutamatergic synapse | link | RTN4R | 3 | 0.005 |
| anchored component of membrane | link | RTN4R | 3 | 0.254 |
| presynapse | link | RTN4R | 2 | 0.060 |
| neuronal cell body | link | RTN4R | 2 | 0.062 |
| **GLAD4U diseases** | | | | |
| Parotitis | link | IL18R1 | 70 | 0.298 |
| Purpura, Thrombocytopenic | link | IL18R1 | 53 | 0.033 |
| SRS-A | link | GGT1 | 51 | 0.002 |
| Purpura, Thrombocytopenic, Idiopathic | link | IL18R1 | 49 | 0.037 |
| Hepatitis, Alcoholic | link | GGT1 | 45 | 2.44e-04 |
| Purpura | link | IL18R1 | 43 | 0.043 |
| Bacteremia | link | CRP | 40 | 3.38e-09 |
| Microvascular Angina | link | CRP | 40 | 9.59e-04 |
| Liver Cirrhosis, Alcoholic | link | GGT1 | 39 | 3.64e-04 |
| Selective immunoglobulin G deficiency | link | CRP | 38 | 4.76e-07 |
| febrile neutropenia | link | CRP | 38 | 8.80e-05 |
| Actinomycetales Infections | link | IL18R1 | 38 | 6.83e-04 |
| Mycobacterial infection | link | IL18R1 | 37 | 6.83e-04 |
| Mycobacterium Infections | link | IL18R1 | 37 | 6.83e-04 |
| Acute necrotizing pancreatitis | link | CRP | 36 | 0.014 |
| Environmental allergy | link | IL18R1 | 36 | 0.005 |
| Blood Loss, Surgical | link | CRP | 34 | 8.97e-09 |
| Periventricular leucomalacia | link | FAS | 34 | 0.005 |
| Streptococcal Infections | link | CRP | 33 | 1.07e-08 |
| Vitamin A Deficiency | link | CRP | 33 | 0.016 |
| Liver Diseases, Alcoholic | link | GGT1 | 31 | 4.71e-06 |
| Psoriasis | link | IL18R1 | 31 | 0.001 |
| Arteritis | link | CRP | 30 | 1.97e-05 |
| Hypoalbuminaemia | link | CRP | 30 | 0.018 |
| Postoperative Hemorrhage | link | CRP | 30 | 0.018 |
| Graves Disease | link | IL18R1 | 30 | 0.008 |
| Osteoarthritis, Knee | link | IL18R1 | 30 | 0.069 |
| Ischemic Attack, Transient | link | CRP | 28 | 2.89e-05 |
| Kidney Tubular Necrosis, Acute | link | CRP | 28 | 0.002 |
| Retinal Drusen | link | CRP | 28 | 0.002 |
| Alcoholic fatty liver | link | GGT1 | 28 | 4.57e-05 |
| Angina Pectoris | link | CRP | 27 | 6.42e-09 |
| Community-Acquired Infections | link | CRP | 27 | 2.74e-04 |
| Escherichia coli Infections | link | CRP | 26 | 4.46e-07 |
| Mumps | link | IL18R1 | 26 | 0.492 |
| Intracranial Arteriosclerosis | link | CRP | 25 | 0.003 |
| major adverse cardiac events (mace) | link | CRP | 25 | 0.003 |
| Osteoarthritis | link | IL18R1 | 25 | 0.013 |
| Malabsorption Syndromes | link | IL18R1 | 24 | 0.099 |
| Smallpox | link | IL18R1 | 24 | 0.099 |
| renal transplant failure | link | CRP | 23 | 1.41e-07 |
| Staphylococcal Infections | link | CRP | 23 | 5.11e-04 |
| Enterocolitis | link | CRP | 23 | 0.004 |
| Microvascular Angina | link | GGT1 | 23 | 0.630 |
| Gastroenteritis | link | IL18R1 | 23 | 6.37e-04 |
| Asthma | link | IL18R1 | 23 | 6.37e-04 |
| Crohn Disease | link | IL18R1 | 23 | 0.002 |
| Occupational Diseases | link | IL18R1 | 23 | 0.100 |
| Acute coronary syndrome | link | CRP | 22 | 1.99e-07 |
| Pregnancy Third Trimester | link | CRP | 22 | 1.15e-05 |
| Acute-Phase Reaction | link | CRP | 22 | 7.94e-05 |
| Arteriovenous Fistula | link | CRP | 22 | 0.031 |
| Respiratory Hypersensitivity | link | IL18R1 | 22 | 0.002 |
| Gram-Positive Bacterial Infections | link | IL18R1 | 22 | 0.017 |
| Urinary Tract Infections | link | CRP | 21 | 0.005 |
| Leprosy | link | IL18R1 | 21 | 0.572 |
| Coronary Stenosis | link | CRP | 20 | 2.08e-06 |
| Cross Infection | link | CRP | 20 | 2.45e-06 |
| disease activity score 28 joint in rheumatoid arthritis | link | CRP | 20 | 0.005 |
| Pulmonary Embolism | link | CRP | 20 | 0.006 |
| Hyperoxia | link | FAS | 20 | 4.52e-04 |
| Lupus Erythematosus, Systemic | link | CRP | 19 | 0.00e+00 |
| Lupus erythematosus | link | CRP | 19 | 0.00e+00 |
| Chronic pericementitis | link | CRP | 19 | 1.95e-05 |
| Arthritis, Reactive | link | CRP | 19 | 2.50e-05 |
| Peripheral Vascular Diseases | link | CRP | 19 | 2.50e-05 |
| Pneumonia, Bacterial | link | CRP | 19 | 9.47e-04 |
| Carotid Stenosis | link | CRP | 19 | 0.001 |
| Congenital choledochal cyst | link | GGT1 | 19 | 0.690 |
| hepatic necrosis | link | FAS | 19 | 8.87e-06 |
| Rubella | link | IL18R1 | 19 | 0.601 |
| Organ Transplantation | link | CRP | 18 | 3.38e-09 |
| Systemic Inflammatory Response Syndrome | link | CRP | 18 | 1.41e-07 |
| Angina, Unstable | link | CRP | 18 | 1.82e-04 |
| Mucocutaneous Lymph Node Syndrome | link | CRP | 18 | 0.001 |
| Embolism | link | CRP | 18 | 0.007 |
| creatinine clearance | link | CRP | 18 | 0.007 |
| Paroxysmal tachycardia NOS | link | CRP | 18 | 0.284 |
| Tachycardia, Paroxysmal | link | CRP | 18 | 0.284 |
| pain crisis | link | CRP | 18 | 0.284 |
| cessation | link | GGT1 | 18 | 0.151 |
| Inflammatory Bowel Diseases | link | IL18R1 | 18 | 0.004 |
| Carotid Artery Diseases | link | CRP | 17 | 1.70e-07 |
| Periodontitis | link | CRP | 17 | 4.64e-05 |
| Pneumococcal Infections | link | CRP | 17 | 2.28e-04 |
| Shock, Septic | link | CRP | 17 | 2.28e-04 |
| adverse cardiovascular events | link | CRP | 17 | 0.002 |
| Sleep Apnea Syndromes | link | CRP | 17 | 0.008 |
| Respiratory Sounds | link | CRP | 17 | 0.008 |
| Sleep Apnea, Obstructive | link | CRP | 17 | 0.008 |
| Hematoma | link | CRP | 17 | 0.304 |
| Bronchial Diseases | link | IL18R1 | 17 | 0.005 |
| Hypersensitivity | link | IL18R1 | 17 | 0.005 |
| Preterm Infant | link | IL18R1 | 17 | 0.033 |
| Respiratory Syncytial Virus Infections | link | IL18R1 | 17 | 0.033 |
| Celiac Disease | link | IL18R1 | 17 | 0.161 |
| glomerular disease | link | CRP | 16 | 9.27e-09 |
| Proteinuria | link | CRP | 16 | 5.25e-08 |
| Periodontal Diseases | link | CRP | 16 | 4.98e-05 |
| Hip Injuries | link | CRP | 16 | 0.053 |
| Lymphopenia | link | FAS | 16 | 8.00e-07 |
| Lymphocytosis | link | FAS | 16 | 0.006 |
| Bronchitis | link | IL18R1 | 16 | 0.038 |
| Sepsis | link | CRP | 15 | 4.20e-07 |
| Kidney Transplantation | link | CRP | 15 | 4.27e-07 |
| Septicemia | link | CRP | 15 | 4.46e-07 |
| Vasculitis | link | CRP | 15 | 4.46e-07 |
| Diabetic Angiopathies | link | CRP | 15 | 7.18e-05 |
| Reperfusion Injury | link | CRP | 15 | 7.54e-05 |
| Lung Diseases, Fungal | link | CRP | 15 | 7.94e-05 |
| Coronary Thrombosis | link | CRP | 15 | 0.002 |
| Fibromyalgia | link | CRP | 15 | 0.056 |
| Tachycardia, Sinus | link | CRP | 15 | 0.320 |
| Bacterial Infections | link | CRP | 14 | 5.22e-11 |
| visceral obesity | link | CRP | 14 | 1.82e-05 |
| Urination Disorders | link | CRP | 14 | 1.06e-04 |
| Periodontal Pocket | link | CRP | 14 | 1.26e-04 |
| Hypertriglyceridemia | link | CRP | 14 | 5.29e-04 |
| Myocardial Reperfusion Injury | link | CRP | 14 | 0.013 |
| Poland anomaly | link | CRP | 14 | 0.014 |
| Intraoperative Complications | link | CRP | 14 | 0.334 |
| visceral obesity | link | GGT1 | 14 | 0.011 |
| Hepatitis, Alcoholic | link | GPT | 14 | 0.622 |
| Inflammation | link | IL18R1 | 14 | 6.83e-04 |
| Respiratory Tract Infections | link | IL18R1 | 14 | 0.047 |
| Traumatic optic neuropathy | link | RTN4R | 14 | 0.181 |
| Gram-Positive Bacterial Infections | link | CRP | 13 | 2.50e-07 |
| Kidney Failure | link | CRP | 13 | 3.33e-07 |
| Hyperlipidemias | link | CRP | 13 | 1.58e-04 |
| Urological Manifestations | link | CRP | 13 | 1.67e-04 |
| Pneumonia | link | CRP | 13 | 1.74e-04 |
| Pneumonia, Pneumococcal | link | CRP | 13 | 1.74e-04 |
| pneumonitis | link | CRP | 13 | 1.82e-04 |
| Aortic Diseases | link | CRP | 13 | 8.81e-04 |
| Chest Pain | link | CRP | 13 | 0.004 |
| Coronary Restenosis | link | CRP | 13 | 0.015 |
| Critical Illness | link | CRP | 13 | 0.015 |
| venous thromboembolism | link | CRP | 13 | 0.016 |
| Anovulation | link | CRP | 13 | 0.350 |
| Fatty Liver | link | GGT1 | 13 | 1.42e-04 |
| hepatic steatosis | link | GGT1 | 13 | 1.64e-04 |
| Multiple Sclerosis, Relapsing-Remitting | link | FAS | 13 | 6.07e-04 |
| Toxemia | link | FAS | 13 | 0.035 |
| Systemic sclerosis, diffuse | link | GDF15 | 13 | 0.056 |
| Drug interaction with food | link | GDF15 | 13 | 0.221 |
| Hemorrhage | link | CRP | 12 | 1.21e-05 |
| Kidney Failure, Chronic | link | CRP | 12 | 4.02e-05 |
| Spondylarthropathies | link | CRP | 12 | 9.59e-04 |
| Spondylarthritis | link | CRP | 12 | 0.001 |
| Macular Degeneration | link | CRP | 12 | 0.001 |
| Urticaria | link | CRP | 12 | 0.088 |
| Graft Occlusion, Vascular | link | CRP | 12 | 0.364 |
| Hyperuricemia | link | GPT | 12 | 0.657 |
| Erythema | link | FAS | 12 | 0.013 |
| Vaccinia | link | IL18R1 | 12 | 0.782 |
| Hypertension, Pulmonary | link | GDF15 | 12 | 3.49e-04 |
| Hypertrophy, Left Ventricular | link | GDF15 | 12 | 0.018 |
| Bronchopulmonary dysplasia of newborn | link | GDF15 | 12 | 0.069 |
| Connective Tissue Diseases | link | CRP | 11 | 1.13e-12 |
| Brain Ischemia | link | CRP | 11 | 9.39e-05 |
| Albuminuria | link | CRP | 11 | 0.006 |
| Cerebral Infarction | link | CRP | 11 | 0.021 |
| Diabetic Neuropathies | link | CRP | 11 | 0.091 |
| Peritonitis | link | CRP | 11 | 0.100 |
| Peripheral Vascular Diseases | link | GGT1 | 11 | 0.327 |
| Death | link | FAS | 11 | 0.00e+00 |
| Thyroiditis | link | FAS | 11 | 0.005 |
| Bronchiolitis | link | IL18R1 | 11 | 0.290 |
| Cachexia | link | GDF15 | 11 | 0.270 |
| Autoimmune Diseases | link | CRP | 10 | 0.00e+00 |
| Coronary Artery Disease | link | CRP | 10 | 2.80e-08 |
| Cerebrovascular Disorders | link | CRP | 10 | 1.77e-06 |
| Ischemia | link | CRP | 10 | 1.91e-06 |
| Rupture | link | CRP | 10 | 1.46e-04 |
| Dyslipidaemia | link | CRP | 10 | 5.29e-04 |
| treatment failure | link | CRP | 10 | 0.002 |
| Fever | link | CRP | 10 | 0.002 |
| Spondylitis | link | CRP | 10 | 0.003 |
| Diabetes, Gestational | link | CRP | 10 | 0.007 |
| Postoperative Complications | link | CRP | 10 | 0.008 |
| Thromboembolism | link | CRP | 10 | 0.026 |
| time in therapeutic range | link | CRP | 10 | 0.109 |
| Prinzmetal angina | link | CRP | 10 | 0.112 |
| Hyperinsulinism | link | GGT1 | 10 | 0.009 |
| Liver Cirrhosis | link | GGT1 | 10 | 0.033 |
| Carotid Artery Diseases | link | GGT1 | 10 | 0.110 |
| Hyperuricemia | link | GGT1 | 10 | 0.944 |
| Drug interaction with drug | link | FAS | 10 | 0.00e+00 |
| Hodgkin Disease | link | FAS | 10 | 6.46e-07 |
| Lung Diseases | link | IL18R1 | 10 | 0.094 |
| Respiratory Tract Diseases | link | IL18R1 | 10 | 0.100 |
| Bacterial Infections | link | IL18R1 | 10 | 0.321 |
| Growth Disorders | link | GDF15 | 10 | 3.48e-06 |
| Bone Neoplasms | link | GDF15 | 10 | 7.77e-05 |
| Arteriosclerosis | link | CRP | 9 | 1.03e-07 |
| Arterial Occlusive Diseases | link | CRP | 9 | 1.03e-07 |
| Kidney Diseases | link | CRP | 9 | 2.04e-07 |
| Rheumatic Diseases | link | CRP | 9 | 4.15e-07 |
| Coronary Disease | link | CRP | 9 | 5.17e-07 |
| Respiratory Tract Infections | link | CRP | 9 | 1.21e-06 |
| Myocardial Infarction | link | CRP | 9 | 2.65e-05 |
| Thrombosis | link | CRP | 9 | 8.20e-04 |
| Gestational hypertension | link | CRP | 9 | 9.11e-04 |
| Preterm rupture of membranes | link | CRP | 9 | 0.001 |
| Brain Infarction | link | CRP | 9 | 0.033 |
| Aortic Aneurysm | link | CRP | 9 | 0.037 |
| Fistula | link | CRP | 9 | 0.120 |
| Hip Fractures | link | CRP | 9 | 0.134 |
| Apnea | link | CRP | 9 | 0.138 |
| Current Non-smoker | link | CRP | 9 | 0.138 |
| Abscess | link | CRP | 9 | 0.436 |
| Metabolic Syndrome | link | GGT1 | 9 | 0.011 |
| Lymphoma, B-Cell | link | FAS | 9 | 1.80e-08 |
| Brain Death | link | FAS | 9 | 2.03e-06 |
| Tumor Virus Infections | link | FAS | 9 | 4.36e-06 |
| Lymphoma, T-Cell, Peripheral | link | FAS | 9 | 0.021 |
| Xerostomia | link | FAS | 9 | 0.023 |
| dry mouth | link | FAS | 9 | 0.023 |
| Pathologic Processes | link | IL18R1 | 9 | 0.035 |
| Anorexia | link | GDF15 | 9 | 0.139 |
| beta-Thalassemia | link | GDF15 | 9 | 0.788 |
| Vascular Diseases | link | CRP | 8 | 9.27e-09 |
| Myocardial Ischemia | link | CRP | 8 | 9.45e-07 |
| Infarction | link | CRP | 8 | 3.11e-05 |
| Chorioamnionitis | link | CRP | 8 | 0.002 |
| Wounds and Injuries | link | CRP | 8 | 0.005 |
| Hypercholesterolemia | link | CRP | 8 | 0.014 |
| Glucose Intolerance | link | CRP | 8 | 0.045 |
| adverse events | link | CRP | 8 | 0.049 |
| Fatigue | link | CRP | 8 | 0.142 |
| Peritoneal Diseases | link | CRP | 8 | 0.142 |
| Kidney Failure, Acute | link | CRP | 8 | 0.161 |
| Ventricular Dysfunction, Left | link | CRP | 8 | 0.161 |
| Arrhythmia, Sinus | link | CRP | 8 | 0.454 |
| Wound Infection | link | CRP | 8 | 0.454 |
| Fatigue Syndrome, Chronic | link | CRP | 8 | 0.482 |
| Liver Diseases, Alcoholic | link | GPT | 8 | 0.507 |
| drug-induced liver injury | link | GPT | 8 | 0.884 |
| Necrosis | link | FAS | 8 | 1.00e-12 |
| Lymphoma, Low-Grade | link | FAS | 8 | 9.41e-08 |
| Leukopenia | link | FAS | 8 | 0.005 |
| Cross Infection | link | FAS | 8 | 0.013 |
| Goiter | link | FAS | 8 | 0.013 |
| Splenomegaly | link | FAS | 8 | 0.088 |
| Sezary Syndrome | link | FAS | 8 | 0.096 |
| Thyroiditis, Autoimmune | link | FAS | 8 | 0.096 |
| overall survival | link | GDF15 | 8 | 6.73e-10 |
| major adverse cardiac events (mace) | link | GDF15 | 8 | 0.837 |
| Spinal Cord Injuries | link | RTN4R | 8 | 0.325 |
| Chronic Disease | link | CRP | 7 | 2.43e-06 |
| Arthritis | link | CRP | 7 | 5.77e-06 |
| Urologic Diseases | link | CRP | 7 | 1.27e-05 |
| Joint Diseases | link | CRP | 7 | 7.18e-05 |
| Pregnancy Complications | link | CRP | 7 | 9.39e-05 |
| Hyperinsulinism | link | CRP | 7 | 0.003 |
| Metabolic Syndrome | link | CRP | 7 | 0.004 |
| glomerular filtration rate | link | CRP | 7 | 0.021 |
| Aneurysm | link | CRP | 7 | 0.061 |
| Body Weight Changes | link | CRP | 7 | 0.062 |
| Malnutrition NOS | link | CRP | 7 | 0.062 |
| Ovarian Cysts | link | CRP | 7 | 0.073 |
| Polycystic Ovary Syndrome | link | CRP | 7 | 0.075 |
| Acute abdomen | link | CRP | 7 | 0.517 |
| Liver Diseases | link | GGT1 | 7 | 0.010 |
| Hepatitis | link | GGT1 | 7 | 0.100 |
| diabetes mellitus type 2 and obesity | link | GGT1 | 7 | 0.217 |
| Lymphoma | link | FAS | 7 | 1.47e-08 |
| Lupus erythematosus | link | FAS | 7 | 3.60e-05 |
| Lupus Erythematosus, Systemic | link | FAS | 7 | 3.60e-05 |
| Lymphoma, Non-Hodgkin | link | FAS | 7 | 1.80e-04 |
| Neoplasms, Radiation-Induced | link | FAS | 7 | 0.001 |
| Neutropenia | link | FAS | 7 | 0.011 |
| Hepatitis C, Chronic | link | FAS | 7 | 0.019 |
| Lung Diseases, Interstitial | link | FAS | 7 | 0.021 |
| Dry Eye Syndromes | link | FAS | 7 | 0.041 |
| Brain Death | link | GDF15 | 7 | 0.012 |
| Hyperoxia | link | GDF15 | 7 | 0.869 |
| Inflammation | link | CRP | 6 | 1.46e-07 |
| Cardiovascular Diseases | link | CRP | 6 | 5.36e-07 |
| Hypertension | link | CRP | 6 | 2.06e-04 |
| Inflammatory Bowel Diseases | link | CRP | 6 | 5.93e-04 |
| Nutrition Disorders | link | CRP | 6 | 7.13e-04 |
| Arthritis, Rheumatoid | link | CRP | 6 | 7.27e-04 |
| Stroke | link | CRP | 6 | 0.002 |
| Stroke NOS | link | CRP | 6 | 0.002 |
| Crohn Disease | link | CRP | 6 | 0.005 |
| Premature Birth | link | CRP | 6 | 0.019 |
| Renal diabetes | link | CRP | 6 | 0.030 |
| Lung Diseases, Obstructive | link | CRP | 6 | 0.040 |
| heart rate | link | CRP | 6 | 0.044 |
| diabetes mellitus type 2 and obesity | link | CRP | 6 | 0.046 |
| Pancreatitis | link | CRP | 6 | 0.081 |
| Vascular Resistance | link | CRP | 6 | 0.087 |
| Ventricular Fibrillation | link | CRP | 6 | 0.220 |
| Ventricular Dysfunction | link | CRP | 6 | 0.270 |
| Diabetes Mellitus | link | GGT1 | 6 | 0.024 |
| Liver Neoplasms | link | GGT1 | 6 | 0.047 |
| Diabetes Mellitus, Type 2 | link | GGT1 | 6 | 0.047 |
| Arteriosclerosis | link | GGT1 | 6 | 0.052 |
| Arterial Occlusive Diseases | link | GGT1 | 6 | 0.052 |
| Insulin Resistance | link | GGT1 | 6 | 0.126 |
| diastolic blood pressure | link | GGT1 | 6 | 0.149 |
| systolic blood pressure | link | GGT1 | 6 | 0.149 |
| Coronary Artery Disease | link | GGT1 | 6 | 0.165 |
| glomerular filtration rate | link | GGT1 | 6 | 0.618 |
| Metabolic Syndrome | link | GPT | 6 | 0.249 |
| visceral obesity | link | GPT | 6 | 0.657 |
| overall survival | link | FAS | 6 | 1.68e-11 |
| Lymphoproliferative Disorders | link | FAS | 6 | 2.10e-07 |
| Lymphatic Diseases | link | FAS | 6 | 4.36e-06 |
| autoimmunity | link | FAS | 6 | 5.46e-04 |
| Leukemia, B-Cell | link | FAS | 6 | 0.003 |
| Lymphoma, T-Cell | link | FAS | 6 | 0.005 |
| Hepatitis, Chronic | link | FAS | 6 | 0.010 |
| Hepatitis B | link | FAS | 6 | 0.011 |
| Lymphoma, T-Cell, Cutaneous | link | FAS | 6 | 0.156 |
| Hepatitis, Autoimmune | link | FAS | 6 | 0.291 |
| Endotoxemia | link | FAS | 6 | 0.310 |
| Autoimmune hemolytic anemia NOS | link | FAS | 6 | 0.635 |
| Intestinal Diseases | link | IL18R1 | 6 | 0.489 |
| Colorectal Neoplasms | link | GDF15 | 6 | 5.77e-04 |
| Intestinal Neoplasms | link | GDF15 | 6 | 6.88e-04 |
| Eating Disorders | link | GDF15 | 6 | 0.046 |
| Scleroderma, Systemic | link | GDF15 | 6 | 0.229 |
| Hemolysis | link | GDF15 | 6 | 0.493 |
| Anemia, Hypochromic | link | GDF15 | 6 | 0.952 |
| Embolism | link | GDF15 | 6 | 0.952 |
| Adolescent | link | CRP | 5 | 2.01e-04 |
| Female Urogenital Diseases and Pregnancy Complications | link | CRP | 5 | 5.29e-04 |
| Obesity | link | CRP | 5 | 6.95e-04 |
| Diabetes Mellitus | link | CRP | 5 | 9.09e-04 |
| Insulin Resistance | link | CRP | 5 | 0.010 |
| Gastroenteritis | link | CRP | 5 | 0.013 |
| diastolic blood pressure | link | CRP | 5 | 0.013 |
| systolic blood pressure | link | CRP | 5 | 0.013 |
| Preterm Infant | link | CRP | 5 | 0.027 |
| Pain | link | CRP | 5 | 0.062 |
| Menopause | link | CRP | 5 | 0.141 |
| Hyperglycemia | link | CRP | 5 | 0.161 |
| Cysts | link | CRP | 5 | 0.164 |
| Osteoarthritis, Knee | link | CRP | 5 | 0.284 |
| Femoral Fractures | link | CRP | 5 | 0.322 |
| Aortic Aneurysm, Abdominal | link | CRP | 5 | 0.628 |
| Coronary Disease | link | GGT1 | 5 | 0.214 |
| Hepatitis, Chronic | link | GGT1 | 5 | 0.770 |
| heart rate | link | GGT1 | 5 | 0.778 |
| Leukemia | link | FAS | 5 | 4.41e-07 |
| Acquired Immunodeficiency Syndrome | link | FAS | 5 | 0.020 |
| Occupational Diseases | link | FAS | 5 | 0.128 |
| Cervical Intraepithelial Neoplasia | link | FAS | 5 | 0.407 |
| Death | link | GDF15 | 5 | 0.004 |
| Glioblastoma | link | GDF15 | 5 | 0.110 |
| Polyps | link | GDF15 | 5 | 0.631 |
| Ectopic pregnancy NOS | link | GDF15 | 5 | 0.969 |
| Thalassemia | link | GDF15 | 5 | 0.969 |
| Anemia, Iron-Deficiency | link | GDF15 | 5 | 0.974 |
| Hemochromatosis | link | GDF15 | 5 | 0.974 |
| Spinal Cord Diseases | link | RTN4R | 5 | 0.012 |
| Spinal Injuries | link | RTN4R | 5 | 0.832 |
| Heart Diseases | link | CRP | 4 | 0.003 |
| Male Urogenital Diseases | link | CRP | 4 | 0.007 |
| Pathologic Processes | link | CRP | 4 | 0.009 |
| Necrosis | link | CRP | 4 | 0.014 |
| Diabetes Mellitus, Type 2 | link | CRP | 4 | 0.022 |
| Pregnancy | link | CRP | 4 | 0.029 |
| Obstetric Labor Complications | link | CRP | 4 | 0.112 |
| Pulmonary Disease, Chronic Obstructive | link | CRP | 4 | 0.227 |
| Heart Failure | link | CRP | 4 | 0.234 |
| Weight gain | link | CRP | 4 | 0.358 |
| creatine kinase | link | CRP | 4 | 0.358 |
| Sleep Disorders | link | CRP | 4 | 0.364 |
| Communicable Diseases | link | CRP | 4 | 0.372 |
| Cerebral Hemorrhage | link | CRP | 4 | 0.692 |
| Tachycardia | link | CRP | 4 | 0.765 |
| Cardiovascular Diseases | link | GGT1 | 4 | 0.290 |
| Obesity | link | GGT1 | 4 | 0.327 |
| Myocardial Ischemia | link | GGT1 | 4 | 0.705 |
| hepatic steatosis | link | GPT | 4 | 0.661 |
| Autoimmune Diseases | link | FAS | 4 | 0.002 |
| Immunologic Deficiency Syndromes | link | FAS | 4 | 0.002 |
| Connective Tissue Diseases | link | FAS | 4 | 0.003 |
| HIV Infections | link | FAS | 4 | 0.005 |
| Arthritis | link | FAS | 4 | 0.011 |
| Arthritis, Rheumatoid | link | FAS | 4 | 0.013 |
| Leukemia, Lymphoid | link | FAS | 4 | 0.015 |
| Lymphoid leukemia NOS | link | FAS | 4 | 0.015 |
| Leukemia, T-Cell | link | FAS | 4 | 0.023 |
| Multiple Sclerosis | link | FAS | 4 | 0.028 |
| Hepatitis | link | FAS | 4 | 0.044 |
| Uterine Cervical Neoplasms | link | FAS | 4 | 0.088 |
| Rupture | link | FAS | 4 | 0.106 |
| transplant rejection | link | FAS | 4 | 0.154 |
| Sepsis | link | FAS | 4 | 0.199 |
| Septicemia | link | FAS | 4 | 0.204 |
| Autoimmune Thyroid Disease | link | FAS | 4 | 0.251 |
| Pregnancy First Trimester | link | FAS | 4 | 0.360 |
| Gastrointestinal Diseases | link | GDF15 | 4 | 0.016 |
| Neoplasm of unspecified nature of digestive system | link | GDF15 | 4 | 0.036 |
| Gastrointestinal Neoplasms | link | GDF15 | 4 | 0.043 |
| Urogenital Neoplasms | link | GDF15 | 4 | 0.068 |
| Hypertension | link | GDF15 | 4 | 0.117 |
| Disease Progression | link | GDF15 | 4 | 0.139 |
| Recurrence | link | GDF15 | 4 | 0.296 |
| Hypertrophy | link | GDF15 | 4 | 0.497 |
| Hemostatic Disorders | link | GDF15 | 4 | 0.550 |
| Pulmonary Heart Disease | link | GDF15 | 4 | 0.665 |
| Body Weight Changes | link | GDF15 | 4 | 0.671 |
| Multiple Sclerosis | link | RTN4R | 4 | 0.139 |
| Signs and Symptoms | link | CRP | 3 | 0.037 |
| event-free survival | link | CRP | 3 | 0.374 |
| Disease Progression | link | CRP | 3 | 0.381 |
| progression-free survival | link | CRP | 3 | 0.388 |
| Cough | link | CRP | 3 | 0.795 |
| Hypertrophy, Left Ventricular | link | CRP | 3 | 0.814 |
| Intracranial Hemorrhages | link | CRP | 3 | 0.860 |
| Subarachnoid Hemorrhage | link | CRP | 3 | 0.860 |
| Vascular Diseases | link | GGT1 | 3 | 0.504 |
| Endocrine System Diseases | link | GGT1 | 3 | 0.504 |
| Endocrine disorder NOS | link | GGT1 | 3 | 0.504 |
| Endocrine disturbance NOS | link | GGT1 | 3 | 0.504 |
| Retroviridae Infections | link | FAS | 3 | 0.011 |
| Sexually Transmitted Diseases | link | FAS | 3 | 0.011 |
| Skin and Connective Tissue Diseases | link | FAS | 3 | 0.011 |
| Lentivirus Infections | link | FAS | 3 | 0.011 |
| Rheumatic Diseases | link | FAS | 3 | 0.044 |
| Joint Diseases | link | FAS | 3 | 0.083 |
| Obstetric Labor Complications | link | FAS | 3 | 0.107 |
| Premature Birth | link | FAS | 3 | 0.201 |
| Demyelinating Diseases | link | FAS | 3 | 0.203 |
| Gestational hypertension | link | FAS | 3 | 0.246 |
| Hepatitis C | link | FAS | 3 | 0.370 |
| Disorder of uterus NOS | link | FAS | 3 | 0.401 |
| Uterine Neoplasms | link | FAS | 3 | 0.401 |
| Organ Transplantation | link | FAS | 3 | 0.502 |
| Pre-Eclampsia | link | FAS | 3 | 0.505 |
| Eclampsia | link | FAS | 3 | 0.510 |
| Mycosis Fungoides | link | FAS | 3 | 0.832 |
| Inflammation | link | GDF15 | 3 | 0.296 |
| Pregnancy | link | GDF15 | 3 | 0.667 |
| Ischemia | link | GDF15 | 3 | 0.718 |
| Renal diabetes | link | GDF15 | 3 | 0.888 |
| Respiratory Tract Diseases | link | CRP | 2 | 0.427 |
| Endocrine System Diseases | link | CRP | 2 | 0.524 |
| Endocrine disorder NOS | link | CRP | 2 | 0.524 |
| Endocrine disturbance NOS | link | CRP | 2 | 0.524 |
| Recurrence | link | CRP | 2 | 0.743 |
| treatment related mortality | link | CRP | 2 | 0.992 |
| Pregnancy Complications | link | FAS | 2 | 0.656 |
| Male Urogenital Diseases | link | GDF15 | 2 | 0.579 |
| Hematologic Diseases | link | GDF15 | 2 | 0.767 |
| Nutrition Disorders | link | GDF15 | 2 | 0.934 |
| Vascular Diseases | link | GDF15 | 2 | 0.954 |
| Central Nervous System Diseases | link | RTN4R | 2 | 0.792 |
| **GLAD4U drugs** | | | | |
| Allergen extracts | link | IL18R1 | 77 | 0.946 |
| Polymyxins | link | CRP | 54 | 1.18e-07 |
| polymyxin b sulfate | link | CRP | 53 | 2.69e-06 |
| nystatin | link | FAS | 33 | 1.63e-06 |
| Inflammation And Infection Detection | link | CRP | 30 | 5.59e-04 |
| acetone | link | GGT1 | 28 | 0.064 |
| serum albumin | link | CRP | 27 | 3.60e-12 |
| Interleukins | link | IL18R1 | 27 | 5.25e-08 |
| interferons | link | IL18R1 | 25 | 1.19e-06 |
| bilirubin | link | GGT1 | 22 | 0.002 |
| Fibrinogen | link | CRP | 21 | 1.07e-12 |
| Viral Vaccines | link | IL18R1 | 19 | 0.543 |
| aminocaproic acid | link | GGT1 | 18 | 0.609 |
| antithymocyte globulin | link | FAS | 18 | 0.075 |
| apixaban | link | GDF15 | 18 | 0.961 |
| human serum albumin | link | CRP | 17 | 2.62e-06 |
| Lipid Modifying Agents | link | CRP | 17 | 0.105 |
| antivirals | link | IL18R1 | 17 | 1.90e-04 |
| Androgens And Female Sex Hormones In Combination | link | CRP | 16 | 0.524 |
| Ultrasound Contrast Media | link | CRP | 16 | 0.524 |
| Glucarpidase | link | GGT1 | 16 | 0.624 |
| trifluoperazine | link | FAS | 16 | 0.003 |
| Immunostimulants | link | IL18R1 | 16 | 0.122 |
| Tests for renal function | link | CRP | 15 | 2.22e-04 |
| Tumor necrosis factor alpha (TNF-alpha) inhibitors | link | FAS | 15 | 0.00e+00 |
| Antiinfectives | link | IL18R1 | 15 | 3.34e-04 |
| Vaccines | link | IL18R1 | 15 | 0.733 |
| atorvastatin | link | CRP | 14 | 0.001 |
| Antibacterials For Systemic Use | link | CRP | 14 | 0.547 |
| vancomycin | link | CRP | 14 | 0.547 |
| Antiseptics And Disinfectants | link | FAS | 13 | 0.028 |
| epipodophyllotoxin | link | FAS | 12 | 7.22e-08 |
| podofilox | link | FAS | 12 | 7.22e-08 |
| Bone morphogenetic proteins | link | GDF15 | 12 | 5.26e-06 |
| streptomycin | link | CRP | 11 | 0.603 |
| bilirubin | link | GPT | 11 | 0.116 |
| etoposide | link | FAS | 11 | 4.83e-08 |
| phospholipids | link | ENPP7 | 11 | 1.23e-04 |
| ginseng | link | RTN4R | 11 | 0.469 |
| aspirin | link | CRP | 10 | 0.020 |
| cellulose | link | GGT1 | 10 | 0.814 |
| hmg coa reductase inhibitors | link | CRP | 9 | 0.007 |
| Fibrates | link | CRP | 9 | 0.080 |
| Tests for diabetes | link | CRP | 8 | 0.004 |
| metformin | link | CRP | 8 | 0.098 |
| simvastatin | link | CRP | 8 | 0.106 |
| lovastatin | link | CRP | 8 | 0.109 |
| omega-3 polyunsaturated fatty acids | link | CRP | 8 | 0.310 |
| pravastatin | link | CRP | 8 | 0.318 |
| protein supplements | link | CRP | 8 | 0.325 |
| l-alanine | link | GGT1 | 8 | 0.048 |
| Drugs used in alcohol dependence | link | GPT | 8 | 0.434 |
| doxorubicin | link | FAS | 8 | 5.72e-05 |
| Drugs For Functional Gastrointestinal Disorders | link | GDF15 | 8 | 0.216 |
| Antifibrinolytics | link | CRP | 7 | 0.345 |
| Corticosteroids For Systemic Use | link | CRP | 7 | 0.692 |
| glucose | link | GGT1 | 7 | 0.001 |
| oxygen | link | GGT1 | 7 | 0.003 |
| papain | link | GGT1 | 7 | 0.904 |
| starch | link | GPT | 7 | 0.500 |
| Proteinase inhibitors | link | FAS | 7 | 2.46e-10 |
| highly active antiretroviral therapy (haart) | link | FAS | 7 | 0.011 |
| Immunoglobulins | link | IL18R1 | 7 | 0.731 |
| immune globulin | link | IL18R1 | 7 | 0.731 |
| Cardiac Therapy | link | CRP | 6 | 0.005 |
| Antiinflammatory Agents | link | CRP | 6 | 0.017 |
| Antihemorrhagics | link | CRP | 6 | 0.097 |
| creatine | link | CRP | 6 | 0.162 |
| triglycerides | link | GGT1 | 6 | 0.112 |
| protease inhibitors | link | FAS | 6 | 1.88e-07 |
| Alkylating Agents | link | FAS | 6 | 0.001 |
| docetaxel | link | GDF15 | 6 | 0.834 |
| Blood coagulation factors | link | CRP | 5 | 5.62e-04 |
| triglycerides | link | CRP | 5 | 0.034 |
| rosuvastatin | link | CRP | 5 | 0.524 |
| alteplase | link | CRP | 5 | 0.547 |
| All Other Therapeutic Products | link | CRP | 5 | 0.821 |
| Other therapeutic products | link | CRP | 5 | 0.821 |
| bnp-32 | link | CRP | 5 | 0.839 |
| nesiritide | link | CRP | 5 | 0.839 |
| sugar | link | GGT1 | 5 | 0.036 |
| glucose | link | GPT | 5 | 0.010 |
| Xanthines | link | GPT | 5 | 0.683 |
| Immunoglobulins | link | FAS | 5 | 6.33e-07 |
| immune globulin | link | FAS | 5 | 6.33e-07 |
| Antiinfectives | link | FAS | 5 | 2.00e-06 |
| antivirals | link | FAS | 5 | 3.80e-05 |
| glucose | link | CRP | 4 | 0.023 |
| sugar | link | CRP | 4 | 0.023 |
| Diagnostic Agents | link | CRP | 4 | 0.433 |
| Other Diagnostic Agents | link | CRP | 4 | 0.620 |
| Tests for pancreatic function | link | CRP | 4 | 0.856 |
| icosapent | link | CRP | 4 | 0.856 |
| l-glutamine | link | GGT1 | 4 | 0.719 |
| Liver therapy | link | GPT | 4 | 0.514 |
| Monoclonal antibodies | link | FAS | 4 | 7.18e-04 |
| interferons | link | FAS | 4 | 0.005 |
| Immunosuppressants | link | FAS | 4 | 0.010 |
| biotin | link | FAS | 4 | 0.057 |
| oxaliplatin | link | FAS | 4 | 0.634 |
| trichostatin A | link | GDF15 | 4 | 0.891 |
| Interleukins | link | CRP | 3 | 0.308 |
| antimetabolites | link | CRP | 3 | 0.551 |
| Vasodilators Used In Cardiac Diseases | link | CRP | 3 | 0.998 |
| bilirubin | link | CRP | 3 | 0.998 |
| sugar | link | GPT | 3 | 0.195 |
| Tumour Detection | link | GDF15 | 3 | 0.977 |
| Other hormones | link | CRP | 2 | 0.727 |
| l-leucine | link | RTN4R | 2 | 0.370 |
| **KEGG pathways** | | | | |
| Sphingolipid metabolism | link | ENPP7 | 136 | 0.00e+00 |
| Glutathione metabolism | link | GGT1 | 85 | 0.00e+00 |
| Taurine and hypotaurine metabolism | link | GGT1 | 67 | 6.33e-06 |
| Inflammatory bowel disease (IBD) | link | IL18R1 | 40 | 0.007 |
| 2-Oxocarboxylic acid metabolism | link | GPT | 34 | 1.37e-05 |
| Arachidonic acid metabolism | link | GGT1 | 32 | 1.38e-10 |
| Biosynthesis of amino acids | link | GPT | 15 | 4.17e-07 |
| Cytokine-cytokine receptor interaction | link | IL18R1 | 15 | 0.002 |
| Carbon metabolism | link | GPT | 13 | 2.20e-08 |
| Apoptosis | link | FAS | 12 | 0.00e+00 |
| TNF signaling pathway | link | FAS | 11 | 1.49e-11 |
| Natural killer cell mediated cytotoxicity | link | FAS | 10 | 4.69e-10 |
| Necroptosis | link | FAS | 9 | 4.90e-11 |
| Chagas disease (American trypanosomiasis) | link | FAS | 9 | 2.54e-07 |
| Platinum drug resistance | link | FAS | 9 | 9.39e-06 |
| African trypanosomiasis | link | FAS | 9 | 0.006 |
| Kaposi sarcoma-associated herpesvirus infection | link | FAS | 8 | 4.90e-11 |
| Allograft rejection | link | FAS | 8 | 0.007 |
| Alanine, aspartate and glutamate metabolism | link | GPT | 7 | 0.261 |
| Human cytomegalovirus infection | link | FAS | 7 | 1.90e-10 |
| Epstein-Barr virus infection | link | FAS | 7 | 2.77e-10 |
| Human immunodeficiency virus 1 infection | link | FAS | 7 | 3.67e-09 |
| Hepatitis B | link | FAS | 7 | 6.57e-07 |
| Measles | link | FAS | 7 | 1.35e-06 |
| Type I diabetes mellitus | link | FAS | 7 | 0.012 |
| Arginine biosynthesis | link | GPT | 6 | 0.882 |
| Proteoglycans in cancer | link | FAS | 6 | 1.47e-08 |
| Herpes simplex infection | link | FAS | 6 | 2.54e-07 |
| Influenza A | link | FAS | 6 | 6.92e-07 |
| Non-alcoholic fatty liver disease (NAFLD) | link | FAS | 6 | 9.39e-06 |
| p53 signaling pathway | link | FAS | 6 | 0.003 |
| Graft-versus-host disease | link | FAS | 6 | 0.046 |
| Alzheimer disease | link | FAS | 5 | 1.36e-04 |
| Autoimmune thyroid disease | link | FAS | 5 | 0.063 |
| MAPK signaling pathway | link | FAS | 4 | 3.11e-04 |
| Cytokine-cytokine receptor interaction | link | GDF15 | 4 | 0.019 |
| Pathways in cancer | link | FAS | 3 | 1.13e-04 |
| Human papillomavirus infection | link | FAS | 3 | 0.002 |
| Cytokine-cytokine receptor interaction | link | FAS | 3 | 0.005 |
| **Molecular function GO-terms** | | | | |
| transferase activity, transferring nitrogenous groups | link | GPT | 33 | 4.04e-04 |
| cytokine receptor activity | link | IL18R1 | 29 | 0.274 |
| cytokine binding | link | IL18R1 | 21 | 0.361 |
| exopeptidase activity | link | GGT1 | 16 | 8.20e-05 |
| lipoprotein particle receptor binding | link | CRP | 15 | 0.152 |
| protein-lipid complex binding | link | CRP | 14 | 0.152 |
| phosphoric ester hydrolase activity | link | ENPP7 | 10 | 0.032 |
| sphingolipid binding | link | RTN4R | 9 | 0.328 |
| glycolipid binding | link | RTN4R | 9 | 0.328 |
| cofactor binding | link | GPT | 7 | 1.33e-10 |
| vitamin binding | link | GPT | 7 | 0.021 |
| transferase activity, transferring acyl groups | link | GGT1 | 5 | 0.286 |
| cytokine receptor binding | link | GDF15 | 4 | 0.076 |
| glycosaminoglycan binding | link | RTN4R | 3 | 0.214 |
| sulfur compound binding | link | RTN4R | 3 | 0.318 |
| **Reactome pathways** | | | | |
| Glutathione conjugation | link | GGT1 | 150 | 0.00e+00 |
| Interleukin-37 signaling | link | IL18R1 | 124 | 6.56e-04 |
| Aflatoxin activation and detoxification | link | GGT1 | 115 | 2.10e-11 |
| Sphingolipid metabolism | link | ENPP7 | 103 | 0.00e+00 |
| CASP8 activity is inhibited | link | FAS | 94 | 0.00e+00 |
| Regulation by c-FLIP | link | FAS | 94 | 0.00e+00 |
| Dimerization of procaspase-8 | link | FAS | 94 | 0.00e+00 |
| Creation of C4 and C2 activators | link | CRP | 85 | 2.44e-12 |
| Initial triggering of complement | link | CRP | 84 | 0.00e+00 |
| Glutathione synthesis and recycling | link | GGT1 | 82 | 2.48e-05 |
| Caspase activation via Death Receptors in the presence of ligand | link | FAS | 65 | 0.00e+00 |
| Glycosphingolipid metabolism | link | ENPP7 | 64 | 4.30e-06 |
| Regulation of necroptotic cell death | link | FAS | 57 | 0.00e+00 |
| Phase II - Conjugation of compounds | link | GGT1 | 56 | 0.00e+00 |
| Complement cascade | link | CRP | 52 | 0.00e+00 |
| RIPK1-mediated regulated necrosis | link | FAS | 52 | 0.00e+00 |
| Regulated Necrosis | link | FAS | 52 | 0.00e+00 |
| Interleukin-1 family signaling | link | IL18R1 | 50 | 3.34e-10 |
| Caspase activation via extrinsic apoptotic signalling pathway | link | FAS | 47 | 0.00e+00 |
| Synthesis of Leukotrienes (LT) and Eoxins (EX) | link | GGT1 | 35 | 0.198 |
| TP53 Regulates Transcription of Death Receptors and Ligands | link | FAS | 31 | 1.87e-04 |
| Biological oxidations | link | GGT1 | 30 | 0.00e+00 |
| Arachidonic acid metabolism | link | GGT1 | 26 | 2.16e-04 |
| TP53 Regulates Transcription of Cell Death Genes | link | FAS | 15 | 1.45e-05 |
| Signaling by Interleukins | link | IL18R1 | 15 | 2.60e-06 |
| Death Receptor Signalling | link | FAS | 13 | 3.29e-14 |
| Amino acid synthesis and interconversion (transamination) | link | GPT | 10 | 0.526 |
| Apoptosis | link | FAS | 10 | 1.02e-11 |
| Programmed Cell Death | link | FAS | 10 | 1.27e-11 |
| Fatty acid metabolism | link | GGT1 | 8 | 0.064 |
| p75 NTR receptor-mediated signalling | link | RTN4R | 4 | 0.066 |
| Metabolism of amino acids and derivatives | link | GPT | 3 | 0.233 |
| Transcriptional Regulation by TP53 | link | FAS | 3 | 0.020 |
| Death Receptor Signalling | link | RTN4R | 3 | 0.094 |
| **Wikipathways** | | | | |
| Glutathione metabolism | link | GGT1 | 82 | 0.00e+00 |
| Cori Cycle | link | GPT | 33 | 0.002 |
| Apoptosis Modulation by HSP70 | link | FAS | 33 | 1.42e-10 |
| Nanomaterial induced apoptosis | link | FAS | 28 | 7.34e-09 |
| Selective expression of chemokine receptors during T-cell polarization | link | IL18R1 | 27 | 0.926 |
| NRF2 pathway | link | GGT1 | 24 | 0.00e+00 |
| Human Complement System | link | CRP | 23 | 0.00e+00 |
| Alanine and aspartate metabolism | link | GPT | 22 | 0.091 |
| Fas Ligand (FasL) pathway and Stress induction of Heat Shock Proteins (HSP) regulation | link | FAS | 20 | 3.57e-11 |
| Apoptosis Modulation and Signaling | link | FAS | 17 | 0.00e+00 |
| Apoptosis | link | FAS | 17 | 0.00e+00 |
| Eicosanoid Synthesis | link | GGT1 | 16 | 0.285 |
| Photodynamic therapy-induced AP-1 survival signaling. | link | FAS | 16 | 3.46e-09 |
| IL-18 signaling pathway | link | IL18R1 | 15 | 2.21e-04 |
| Selenium Micronutrient Network | link | GGT1 | 13 | 5.06e-04 |
| Urea cycle and associated pathways | link | GPT | 13 | 0.198 |
| Nuclear Receptors Meta-Pathway | link | GGT1 | 11 | 6.27e-14 |
| Vitamin D-sensitive calcium signaling in depression | link | GGT1 | 10 | 0.622 |
| Alzheimers Disease | link | FAS | 10 | 2.50e-07 |
| Hepatitis B infection | link | FAS | 8 | 3.25e-08 |
| Spinal Cord Injury | link | RTN4R | 8 | 5.00e-07 |
| Allograft Rejection | link | FAS | 7 | 3.03e-04 |
| DNA Damage Response | link | FAS | 7 | 4.27e-04 |
| miRNA Regulation of DNA Damage Response | link | FAS | 7 | 4.94e-04 |
| Nonalcoholic fatty liver disease | link | FAS | 6 | 2.06e-05 |
| T-Cell antigen Receptor (TCR) Signaling Pathway | link | FAS | 6 | 0.002 |
| Control of immune tolerance by vasoactive intestinal peptide | link | FAS | 6 | 0.425 |
| IL-18 signaling pathway | link | FAS | 4 | 2.12e-04 |
| MAPK Signaling Pathway | link | FAS | 4 | 2.45e-04 |
| VEGFA-VEGFR2 Signaling Pathway | link | FAS | 3 | 0.002 |
| Adipogenesis | link | FAS | 3 | 0.111 |
| Genes involved in male infertility | link | FAS | 2 | 0.526 |
| Epithelial to mesenchymal transition in colorectal cancer | link | GDF15 | 2 | 0.700 |
| 22q11.2 Deletion Syndrome | link | RTN4R | 2 | 0.939 |
| Circadian rhythm related genes | link | FAS | 1 | 0.896 |

#### About

Over-representation analysis (ORA) conducted using WebGestaltR (see
tool-publication here).
Enriched terms are calculated based on the supplied targets as a
collected group against all transcripts/proteins measured in
RHAPSODY.  
Significant terms with a FDR-adjusted p-value < 0.05 are shown in
blue,
while non-significant terms are shown in gray. The
genes/proteins affiliated with the term are shown to the right of the
barplot.

Unfortunately, the analysis will not always have an output. In these
cases the following error message will be shown:  
`Unfortunately, no enriched GO-terms were found for the selected targets.`

### 4.2.2 Shared features

Over-represented terms were found for:

#### Biological process GO-terms

```
## [1] "Unfortunately, no enriched terms were found for the selected targets."
```

#### Cellular component GO-terms

```
## [1] "Unfortunately, no enriched terms were found for the selected targets."
```

#### Molecular function GO-terms

```
## [1] "Unfortunately, no enriched terms were found for the selected targets."
```

#### KEGG pathways

```
## [1] "Unfortunately, no enriched terms were found for the selected targets."
```

#### Reactome pathways

```
## [1] "Unfortunately, no enriched terms were found for the selected targets."
```

#### Wikipathways

```
## [1] "Unfortunately, no enriched terms were found for the selected targets."
```

#### GLAD4U diseases

```
## [1] "Unfortunately, no enriched terms were found for the selected targets."
```

#### GLAD4U drugs

```
## [1] "Unfortunately, no enriched terms were found for the selected targets."
```

#### Table

```
## Error in table_WGR_shared$kbl_table[[1]]: subscript out of bounds
```

```
## Error in table_WGR_shared$kbl_table[[1]]: subscript out of bounds
```

```
## Error in table_WGR_shared$prep_format[[1]]: subscript out of bounds
```

#### About

Over-represtation analysis (ORA) conducted using WebGestaltR (see
tool-publication here).
Enriched terms are calculated based on the supplied targets as a
collected group against all transcripts/proteins measured in
RHAPSODY.  
Significant terms with a FDR-adjusted p-value < 0.05 are shown in
blue,
while non-significant terms are shown in gray. The
genes/proteins affiliated with the term are shown to the right of the
barplot.

Unfortunately, the analysis will not always have an output. In these
cases the following error message will be shown: 
`Unfortunately, no enriched GO-terms were found for the selected targets.`

# 5 Biomarker potential

## 5.1 Drug interactions

### Table

Drug interactions

|  | ATC codes | Drug groups | Target type | Pharmacological action | Evidence for interaction |
| --- | --- | --- | --- | --- | --- |
| **CRELD1** | | | | | |
| – | – | – | – | – | – |
| **CRP** | | | | | |
| CRx-139 | – | Investigational | Target | Unknown | link |
| N,N,N-Trimethyl-2-(phosphonooxy)ethanaminium | – | Experimental | Target | Unknown | link |
| **ENPP7** | | | | | |
| – | – | – | – | – | – |
| **FAS** | | | | | |
| – | – | – | – | – | – |
| **GDF15** | | | | | |
| – | – | – | – | – | – |
| **GGT1** | | | | | |
| Glutathione | V03AB32 | Approved, Investigational, Nutraceutical | Target | Unknown | link |
| **GPT** | | | | | |
| Alanine | – | Nutraceutical | Target | Unknown | link |
| Glutamic acid | A09AB01 | Approved, Nutraceutical | Target | Unknown | link |
| Phenelzine | N06AF03 | Approved | Target | Unknown | link |
| Pyridoxal phosphate | A11HA06 | Approved, Investigational, Nutraceutical | Target | Unknown | link |
| **IL18R1** | | | | | |
| – | – | – | – | – | – |
| **RTN4R** | | | | | |
| – | – | – | – | – | – |

### About

Table of target-drug interactions from DrugBank:

- **ATC codes**: Associated ATC code(s). For further
  information on the ATC system, see the WHO Collaborating Centre for Drug
  Statistics’ website.
- **Drug groups**: Drugs are categorized by group
  determined by their drug development status.:

  - Approved: A drug that has been approved in at least one
    jurisdiction, at some point in time. This does not mean the drug is
    currently approved or available, just that it has been approved and
    marketed at some point, somewhere. Different jurisdictions also have a
    different concept for “approval”. For example drugs that are available
    over-the-counter in the U.S. may not be technically approved, whereas
    over-the-counter drugs in Canada are considered approved.
  - Vet Approved: A drug that has been approved in at least one
    jurisdiction, at some point in time for the treatment of animals.
  - Nutraceutical: A drug that is a pharmaceutical-grade and
    standardized nutrient (with confirmed or unconfirmed health
    benefits).
  - Illicit: A drug that is scheduled in at least one jurisdiction, at
    some point in time.
  - Withdrawn: A previously approved drug that has been withdrawn from
    the market in at least one jurisdiction, at some point in time. Note
    that because a drug can be approved in one jurisdiction, and withdrawn
    in another, it’s possible for a drug to be in both groups. If a drug has
    been approved at some point in time, and then withdrawn, it would also
    be in both drug groups.
  - Investigational: A drug that is in some phase of the drug approval
    process in at least one jurisdiction.
  - Experimental: A compound that has been shown experimentally to bind
    specific proteins in mammals, bacteria, viruses, fungi, or parasites.
    This includes compounds that are Pre-Investigational New Drug
    Applications (Pre-IND, or Discovery Phase compounds).
- **Target type**: Protein targets of drug action,
  enzymes that are inhibited/induced or involved in metabolism, and
  carrier or transporter proteins involved in movement of the drug across
  biological membranes.  Colored according to type using color scheme from
  DrugBank.
- **Pharmacological action**:

  - Yes: A interaction is marked as ‘Yes’ if the drug interacts directly
    with the target as part of the drug’s mechanism of action.
  - No: A interaction is marked as ‘No’ if the interaction between
    target and drug is not directly involved in the drug’s mechanism of
    action.
  - Unknown: A interaction is marked as ‘Unknown’ if currently there is
    no formal research or evidence to elucidate whether interaction between
    the target and drug is associated with the drug’s mechanism of action at
    all.  Colored according to action using color scheme from
    DrugBank.
- **Evidence for interaction**: Link to the
  target-drug interaction information on the DrugBank website with
  references to primary literature on the interaction.

## 5.2 Antibody count

### Plot

Antibody availability

### About

Antibody count from PHAROS (data originates from antibodypedia).

- **Total antibody count**: Total number of commercially
  available antibodies of any type.
- **Monoclonal antibodies**: Number of commercially
  available monoclonal antibodies.
- **Polyclonal antibodies**: Derived number of
  commercially available polyclonal antibodies. Calculated from total and
  monoclonal antibodies. This should only be considered an estimate, as a
  small minority of antibodies fall outside of the two categories of
  monoclonal and polyclonal antibodies, e.g. recombinant antibodies.

## 5.3 Novelty

### Plot

Novelty

### About

The novelty scores shown here is pulled from PHAROS. This score is
calculated from text mining results using the formula \(N\_{i} = \frac{1}{\sum \frac{1}{T\_{k}}}\),
where \(N\_{i}\) is the novelty score
for the target \(i\) and \(T\_{k}\) is the number of targets in
abstracts \(k\). The \(\log\_{10}\) value of \(N\_{i}\) is reported to avoid too high
skewness in the data. A more negative value should be interpreted as
lower novelty, i.e. the target has been studied/reported more and the
target is less novel.

# About the report

This report is intended to provide objective measures that can be
used to perform informed biomarker prioritization.  
It incorporates experimental RHAPSODY data, public data from multiple
databases, data from incorporated R-libraries, and in-house generated
text-mining data.

## Data source overview

**RHAPSODY data**

- Selected targets chosen through the RHAPSODY Biomarker
  Prioritization interface.
- Reference experimental data from all RHAPSODY experiments.

**Public data**

- UniProt website
- The Human Protein Atlas (HPA) website
- Open Targets website
- PHAROS website
- DrugBank website
- STRING website
- WebGestalt website

**In-house generated data**

- Text-mining of 15 million full-text scientific articles Westergaard et
  al. PLoS Computational Biology 2018

## Technical information

Database version information

| Database | Version | Version release date | Download date |
| --- | --- | --- | --- |
| DrugBank | 5.1.9 | 2022-01-04 | 2022-07-18 |
| HPA | 21.1 | 2022-05-31 | 2022-08-04 |
| Open Targets | 22.06 | 2022-06-24 | 2022-08-04 |
| PHAROS | TCRD v6.12.4 | 2021-10-29 | 2022-01-28 |
| UniProt | 2022\_03 | 2022-08-03 | 2022-08-04 |

```
## R version 4.2.1 (2022-06-23)
## Platform: x86_64-apple-darwin17.0 (64-bit)
## Running under: macOS Big Sur ... 10.16
## 
## Matrix products: default
## BLAS:   /Library/Frameworks/R.framework/Versions/4.2/Resources/lib/libRblas.0.dylib
## LAPACK: /Library/Frameworks/R.framework/Versions/4.2/Resources/lib/libRlapack.dylib
## 
## locale:
## [1] en_US.UTF-8/en_US.UTF-8/en_US.UTF-8/C/en_US.UTF-8/en_US.UTF-8
## 
## attached base packages:
## [1] stats     graphics  grDevices utils     datasets  methods   base     
## 
## other attached packages:
##  [1] WebGestaltR_0.4.4  EnvStats_2.7.0     tidygraph_1.2.2    ggraph_2.1.0      
##  [5] Cairo_1.6-0        patchwork_1.1.2    ggnewscale_0.4.8   RColorBrewer_1.1-3
##  [9] ggrepel_0.9.1      formattable_0.2.1  kableExtra_1.3.4   writexl_1.4.1     
## [13] readxl_1.4.2       knitr_1.40         janitor_2.1.0      forcats_1.0.0     
## [17] stringr_1.5.0      dplyr_1.1.0        purrr_1.0.1        readr_2.1.4       
## [21] tidyr_1.3.0        tibble_3.2.0       ggplot2_3.4.1      tidyverse_1.3.2   
## 
## loaded via a namespace (and not attached):
##  [1] googledrive_2.0.0   colorspace_2.0-3    ellipsis_0.3.2     
##  [4] rprojroot_2.0.3     snakecase_0.11.0    fs_1.5.2           
##  [7] rstudioapi_0.14     farver_2.1.1        graphlayouts_0.8.3 
## [10] bit64_4.0.5         fansi_1.0.3         lubridate_1.9.2    
## [13] xml2_1.3.3          codetools_0.2-18    doParallel_1.0.17  
## [16] cachem_1.0.6        polyclip_1.10-4     jsonlite_1.8.4     
## [19] apcluster_1.4.10    broom_1.0.4         dbplyr_2.3.1       
## [22] ggforce_0.4.1       compiler_4.2.1      httr_1.4.5         
## [25] backports_1.4.1     Matrix_1.5-1        fastmap_1.1.0      
## [28] gargle_1.2.1        cli_3.4.1           tweenr_2.0.2       
## [31] htmltools_0.5.3     tools_4.2.1         igraph_1.3.5       
## [34] gtable_0.3.1        glue_1.6.2          doRNG_1.8.2        
## [37] Rcpp_1.0.9          cellranger_1.1.0    jquerylib_0.1.4    
## [40] vctrs_0.5.2         svglite_2.1.0       iterators_1.0.14   
## [43] xfun_0.34           ps_1.7.1            rvest_1.0.3        
## [46] timechange_0.2.0    lifecycle_1.0.3     rngtools_1.5.2     
## [49] googlesheets4_1.0.1 MASS_7.3-58.1       scales_1.2.1       
## [52] vroom_1.6.0         hms_1.1.2           parallel_4.2.1     
## [55] curl_4.3.3          yaml_2.3.6          gridExtra_2.3      
## [58] sass_0.4.2          stringi_1.7.8       highr_0.9          
## [61] foreach_1.5.2       rlang_1.0.6         pkgconfig_2.0.3    
## [64] systemfonts_1.0.4   evaluate_0.17       lattice_0.20-45    
## [67] htmlwidgets_1.5.4   labeling_0.4.2      bit_4.0.4          
## [70] tidyselect_1.2.0    processx_3.7.0      here_1.0.1         
## [73] plyr_1.8.7          magrittr_2.0.3      R6_2.5.1           
## [76] generics_0.1.3      DBI_1.1.3           pillar_1.8.1       
## [79] haven_2.5.2         whisker_0.4         withr_2.5.0        
## [82] modelr_0.1.10       crayon_1.5.2        utf8_1.2.2         
## [85] tzdb_0.3.0          rmarkdown_2.17      viridis_0.6.2      
## [88] grid_4.2.1          callr_3.7.2         reprex_2.0.2       
## [91] digest_0.6.30       webshot_0.5.4       munsell_0.5.0      
## [94] viridisLite_0.4.1   bslib_0.4.0
```
